# Role of torsional strain in the ring-opening polymerisation of low strain [*n*]nickelocenophanes†
†Electronic supplementary information (ESI) available: Computational methods, additional schemes, NMR and MALDI-TOF spectra, DLS and DSC data, crystallographic data. CCDC 1919308–1919311. For ESI and crystallographic data in CIF or other electronic format see DOI: 10.1039/c9sc02624j


**DOI:** 10.1039/c9sc02624j

**Published:** 2019-09-10

**Authors:** Rebecca A. Musgrave, Rebekah L. N. Hailes, Vincent T. Annibale, Ian Manners

**Affiliations:** a School of Chemistry , University of Bristol , Bristol BS8 1TS , UK . Email: imanners@uvic.ca; b Department of Chemistry , University of Victoria , Victoria , BC V8W 3V6 , Canada

## Abstract

Ring-opening polymerisation (ROP) of strained [1]- and [2]metallocenophanes and related species is well-established, and the monomer ring-strain is manifest in a substantial tilting of the cyclopentadienyl ligands, giving *α* angles of ∼14–32°. Surprisingly, tetracarba[4]nickelocenophane [Ni(η^5^-C_5_H_4_)_2_(CH_2_)_4_] (**2**) undergoes ROP (pyridine, 20 °C, 5 days) to give primarily insoluble poly(nickelocenylbutylene) [Ni(η^5^-C_5_H_4_)_2_(CH_2_)_4_]_*n*_ (**12**), despite the lack of significant ring-tilt. The exoenthalpic nature of the ROP was confirmed by DFT calculations involving the cyclic precursor and model oligomers (Δ*H*0ROP = –14 ± 2 kJ mol^–1^), and is proposed to be a consequence of torsional strain present in the *ansa* bridge of **2**. The similarly untilted disila-2-oxa[3]nickelocenophanes [Ni(η^5^-C_5_H_4_)_2_(SiMe_2_)_2_O] (**13**) and [Ni(η^5^-C_5_H_4_)_2_(SiMePh)_2_O] (**14**) were found to lack similar torsional strain and to be resistant to ROP under the same conditions. In contrast, 1-methyltricarba[3]nickelocenophane {Ni(η^5^-C_5_H_4_)_2_(CH_2_)_2_[CH(CH_3_)]} (**15**) with a significant tilt angle (*α* ∼ 16°) was found to undergo ROP to give soluble polymer {Ni(η^5^-C_5_H_4_)_2_(CH_2_)_2_[CH(CH_3_)]}_*n*_ (**18**). The reversibility of the process in this case allowed for the effects of temperature and reaction concentration on the monomer–polymer equilibrium to be explored and thereby thermodynamic data to be elucidated (Δ*H*0ROP = –8.9 kJ mol^–1^, Δ*G*0ROP = –3.1 kJ mol^–1^). Compared to the previously described ROP of the unsubstituted analogue [Ni(η^5^-C_5_H_4_)_2_(CH_2_)_3_] (**1**) (Δ*H*0ROP = –10 kJ mol^–1^, Δ*G*0ROP = –4.0 kJ mol^–1^), the presence of the additional methyl substituent in the *ansa* bridge appears to marginally disfavour ROP and leads to a corresponding decrease in the equilibrium polymer yield.

## Abstract 2

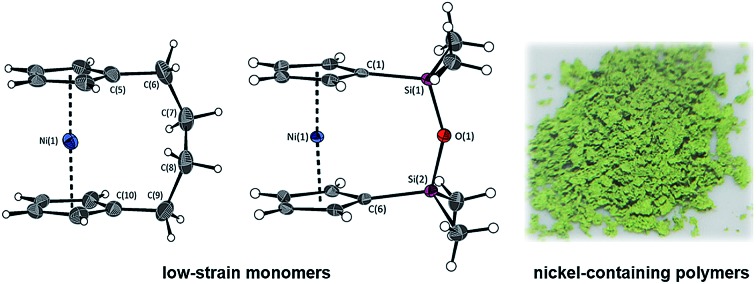
We investigate types of ring strain responsible for the ring-opening polymerisation of [*n*]nickelocenophanes with carbon and silicon-based *ansa* bridges.

## Introduction

Metal-containing polymers (metallopolymers) are of widespread interest due to the large variety of properties resulting from incorporation of a diverse range of metal centres.^1^–^17^ The metals can be located either in the main-chain of the polymer or in side groups, and the interactions between the metal ions and ligands can be strong, leading to essentially static binding, or weak, which can allow for a reversible, dynamic nature.^18^ Metallopolymers have proven crucial in a variety of applications, including data storage,^6^ antibacterial activity,^7^ artificial metalloenzymes,^8^,^9^ emissive materials,^10^,^11^ nanopatterning,^12^–^14^ stimuli-responsive behaviour,^15^ and sensors.^16^,^17^

**Fig. 1:**
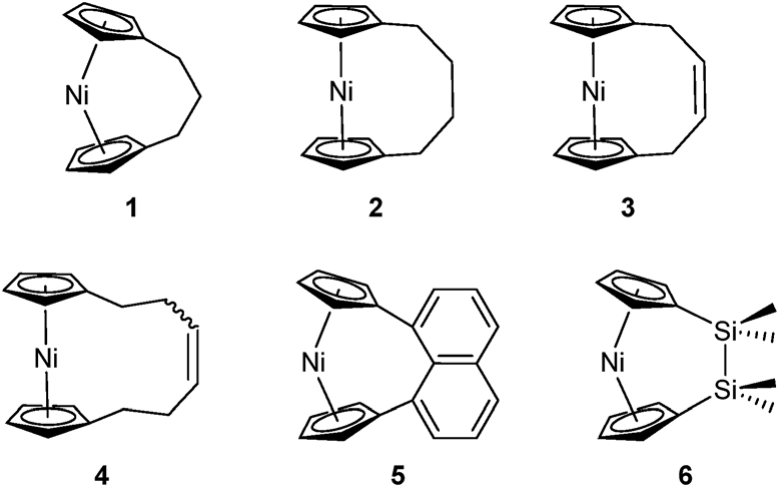
Currently structurally characterised [*n*]nickelocenophanes.

Since the first report of the ring-opening polymerisation (ROP) of an [*n*]ferrocenophane in 1992,^19^ this method has become a well-established pathway to main-chain iron-containing polymers with a variety of bridging elements. The resulting polyferrocenes have attracted interest as a result of their redox responsivity,^20^–^22^ self-assembly behaviour,^23^–^27^ and preceramic properties^28^,^29^ amongst others.^30^ In contrast, polynickelocenes, synthesised *via* the ROP of [*n*]nickelocenophanes, are limited in number. For the 20 valence electron (VE) [*n*]nickelocenophanes, the two extra electrons (compared to 18 VE [*n*]ferrocenophanes) are accommodated in molecular orbitals with antibonding character, which results in a weaker and elongated Ni–Cp bond.^31^ This bond elongation causes a concomitant increase in the angle between the Cp ring planes, *α*, compared to analogous iron and cobalt species,^32^–^34^ and the low bond strength helps to explain the small number of reported [*n*]nickelocenophanes (all structurally characterised examples, **1–6**, are displayed in Fig. 1).^34^–^37^

**Scheme 1:**
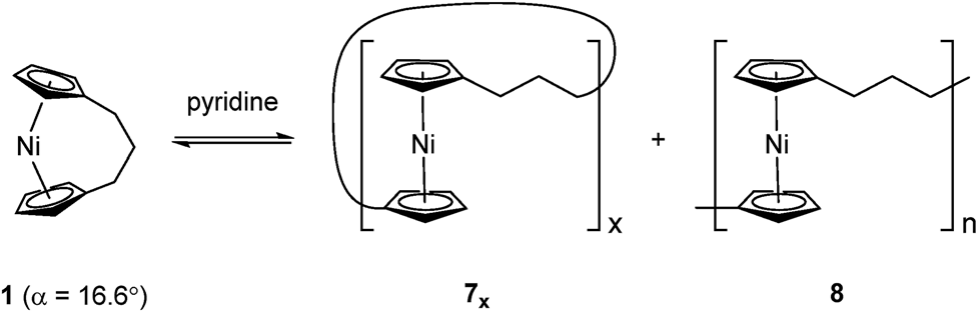
Reversible ROP of tricarba[3]nickelocenophane, **1**.

Recently the ROP of tricarba[3]nickelocenophane **1** to yield polynickelocene **7_x_**/**8** was reported.^35^,^38^ It was demonstrated that **7_x_**/**8** exists in a labile state in which a dynamic equilibrium with **1** is evident in polar organic solvents (Scheme 1). This was postulated to be a consequence of both the weak Ni–Cp bonds (the presumably homolytic M–Cp dissociation energy based in combustion experiments is 250 kJ mol^–1^ for nickelocene *vs.* 305 kJ mol^–1^ for ferrocene), and the reduced energy penalty upon tilting for nickelocene *vs.* ferrocene.^38^,^39^

**Fig. 2:**
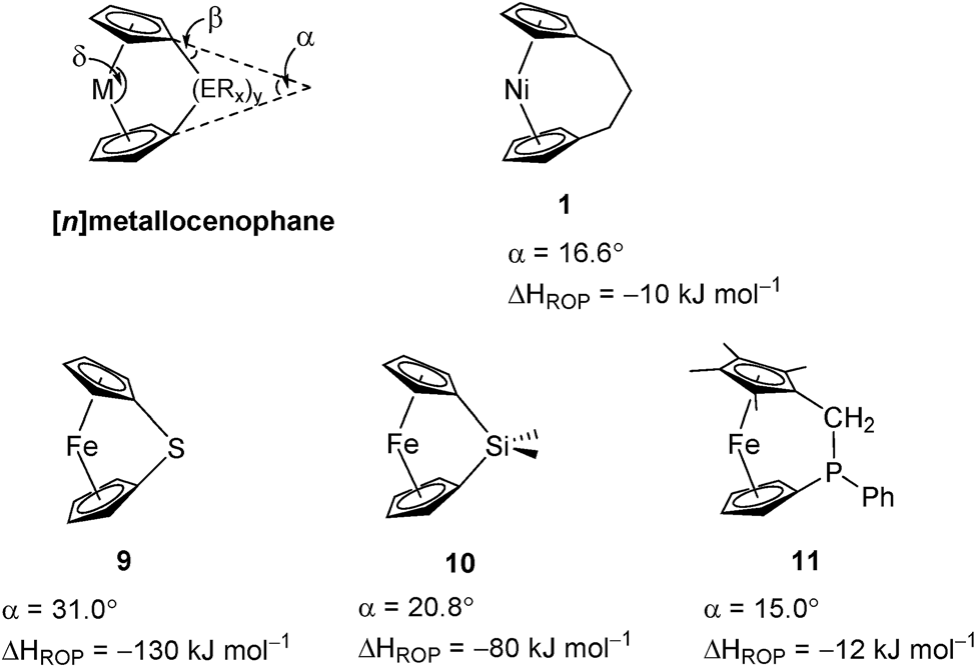
Geometric parameters characterising structural distortions in [*n*]metallocenophanes, and species **1**, **9**, **10**, **11** with their respective tilt angles.

Variable temperature ^1^H NMR spectroscopy allowed for the elucidation of the enthalpic and entropic parameters characterising this ROP process: a small, favorable value of Δ*H* (–10 kJ mol^–1^), and a very small, unfavorable value for Δ*S* (–20 J K^–1^ mol^–1^).^38^ In general, the thermodynamic driving force for the ROP of [*n*]metallocenophanes is ascribed to the strain in these precursors,^40^–^44^ which is generally quantified by the tilt angle, *α*, the Cp_cent_–Cp_ipso_–E angle, *β* (Cp_cent_ = Cp centroid, Cp_ipso_ = Cp ipso carbon, E = bridging element), and the angle *δ*, which is defined as the Cp_cent_–M–Cp_cent_ angle (Fig. 2). Estimations of the value for the ROP enthalpy, Δ*H*_ROP_, for [*n*]ferrocenophanes were previously made on the basis of differential scanning calorimetry (DSC) analyses of the exotherms associated with ROP. These values range from ∼–12 kJ mol^–1^ for carbaphospha[2]ferrocenophane **11** (*α* = 14.9°)^47^ to ∼–130 kJ mol^–1^ for thia[1]ferrocenophane **9** (*α* = 31.0°),^48^ with the Δ*H*_ROP_ value for the well-known dimethylsila[1]ferrocenophane **10** being –72 kJ mol^–1^ (*α* = 20.8°) (Fig. 2).^32^,^43^ Further variations in ROP exotherms have also been reported as a result of the alteration of sterics at the *ansa* bridge position.^45^,^46^ The value for the ROP of tricarba[3]nickelocenophane is comparable to that of **11**, with which it has a comparable tilt angle (**1**: *α* = 16.6°).^38^,^47^

As it has been shown that the tilting of ferrocene invokes a higher energy penalty than nickelocene, the observed Δ*H*_ROP_ value for **11** is smaller than expected based on tilt angle alone, but this is presumably due to the large phenyl substituent in the *ansa* bridge of **11** (steric bulk in the bridge has been previously shown to reduce Δ*H*_ROP_).^38^,^45^ The Δ*H*_ROP_ value for **1** is similar to those for moderately strained rings: *e.g.* THF (Δ*H*0ROP = –19 kJ mol^–1^) and hexamethylcyclotrisiloxane (Δ*H*0ROP = –23 kJ mol^–1^).^49^

Whilst the polymerisation of **1** is a highly unusual example of [*n*]metallocenophane ROP due the presence of a dynamic equilibrium, these types of reversible ROP are more common for organic and inorganic cyclic species, *e.g.* THF in the presence of Lewis acids;^50^ cyclopentene in the presence of various transition metal alkylidene complexes;^51^,^52^ and substituted cyclic six-membered carbonates in the presence of DBU.^53^

**Fig. 3:**
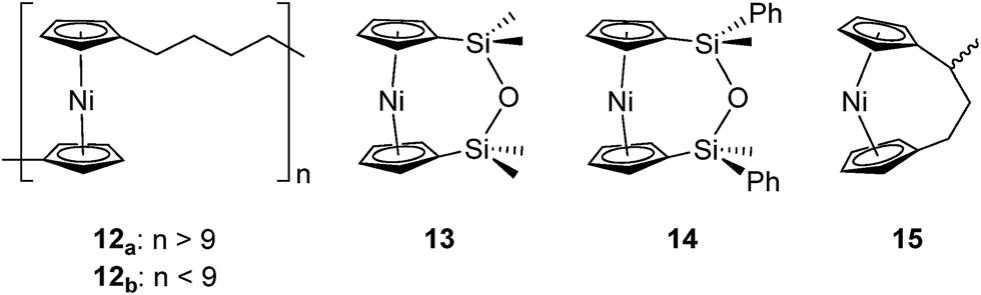
Nickelocene-containing species discussed herein.

Based on the small value for the Δ*H*0ROP of **1**,^38^ which has a tilt angle of 16.6°, we were recently surprised to find that tetracarba[4]nickelocenophane **2**, which possesses a negligible tilt angle (*α* = 1.0(3)°; *δ* = 178.63(11)°), also undergoes ROP (pyridine, 0.74 M, 5 days, 20 °C).^54^ The resulting polynickelocene **12** (Fig. 3) was isolated as a predominantly insoluble material. Herein we examine the nature of the thermodynamic driving force for the ROP of **2** using DFT calculations. We also describe and attempt to explain the ROP behaviour of several new [3]nickelocenophanes **13**, **14**, and **15** (Fig. 3).

## Results and discussion

### Comparative structural data for tricarba[3]nickelophane **1** and tetracarba[4]nickelophane **2**

**Fig. 4:**
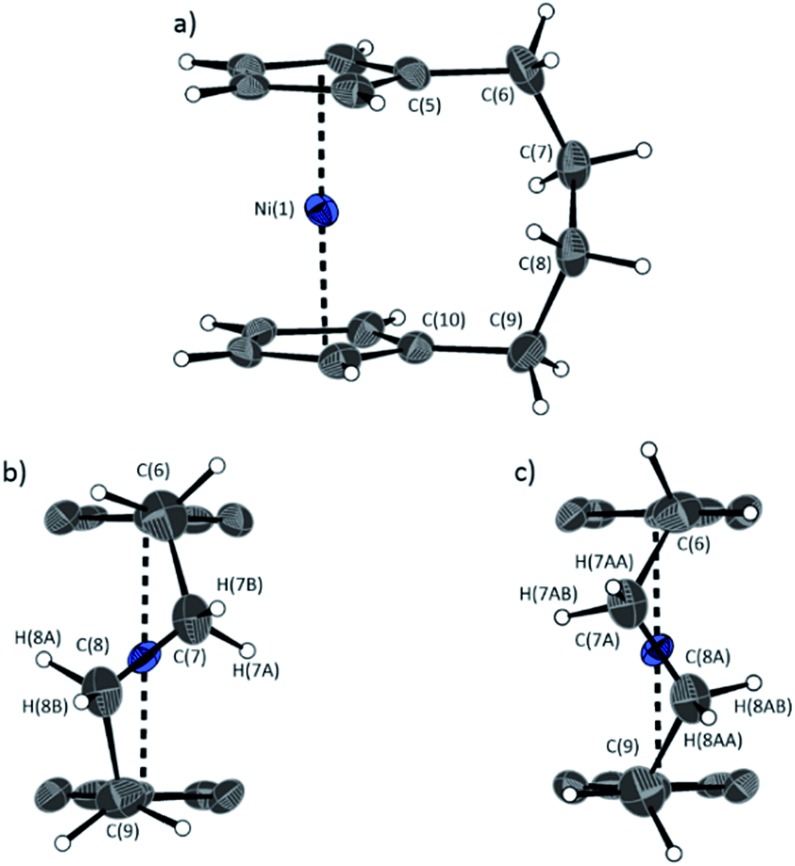
Three views of the molecular structure of **2**.^54^ Thermal ellipsoids displayed at the 50% probability level. Hydrogen atoms are pictured as spheres of arbitrary radii (and some have been omitted for clarity). (a) The *ansa* bridge is disordered at two positions: C7/7A and C8/8A (those with highest relative occupancy (62%) are displayed). Alternate view of **2** displaying (b) major (62%) and (c) minor (38%) component of disordered bridge. Selected distances (Å) and angles (°) for major component (62%): Ni(1)–Cp_cent_ 1.813(3)/1.817(3), *α* = 1.0(3), *δ* = 178.63(11).

**Fig. 5:**
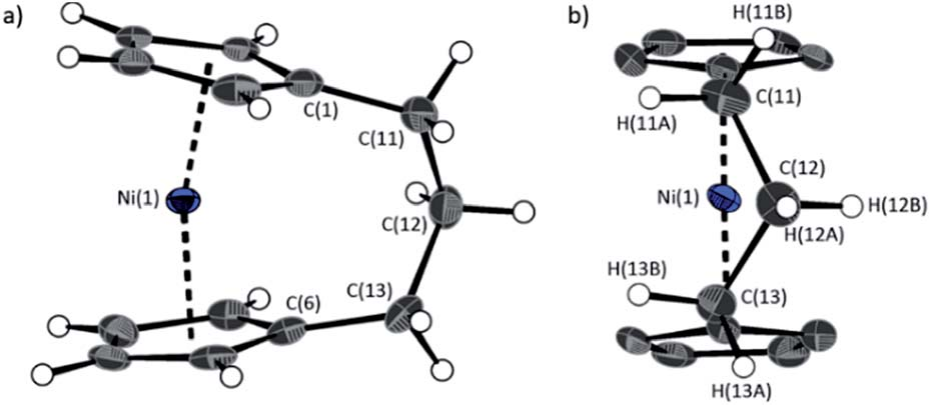
Two views (a and b) of the molecular structure of **1**.^35^ Thermal ellipsoids displayed at the 50% probability level. Hydrogen atoms are pictured as spheres of arbitrary radii (and some have been omitted for clarity). Selected distances (Å) and angles (°): Ni(1)–Cp_cent_ = 1.8039(14)/1.8035(14), *α* = 16.64(13), *β* = 4.2(3), *δ* = 166.33(5).

The lack of ring-tilt exhibited by **2** demonstrates that the thermodynamic driving force for this polymerisation is clearly not manifested in the reduction of tilt upon ring-opening. Whilst the ROP of **2** may still be exoenthalpic, it is not feasible to prove this experimentally as in the case of **1**, due to the insolubility of polymeric **12** at 20 °C and the resulting lack of dynamic equilibrium.^54^ It should be noted that the ROP of **2** is reversible at higher temperatures (above 50 °C), presumably as heating is necessary to solubilise the high molar mass component of **12**. Ring-strain energy in cyclic monomers is manifested in the enthalpic component of the free energy of ring-opening, which can also comprise other contributions to strain in addition to that resulting from ring tilt about the metal centre. These include: angle strain, caused by deviation of bond angles from the ideal; torsional strain, due to repulsion occurring when ring substituents separated by three bonds appear in an eclipsed conformation instead of the more stable staggered conformation; and transannular strain, generated by non-bonding interactions between ring substituents on non-adjacent atoms. Thus, these aspects of strain must also be considered when analysing the ROP of **2**. The structures of **2** and **1**,^35^,^54^ shown in Fig. 4 and 5, respectively, possess different degrees of these types of strain.

Firstly, whilst both **1** and **2** appear to exhibit angle strain, as the bond angles within the *ansa* bridge deviate from the ideal tetrahedral geometry (109.5°), these deviations are smaller in **2** (C(5)–C(6)–C(7): 113.9(6)°, C(6)–C(7)–C(8): 112.4(7)°, C(7)–C(8)–C(9): 115.3(7)°, C(8)–C(9)–C(10): 114.6(6)°) than in **1** (C(1)–C(11)–C(12): 115.8(3)°, C(11)–C(12)–C(13): 115.0(2)°) (Table 1). While this does not eliminate angle strain in **2** as a contributor to its ROP propensity, there appears to be a far more significant factor: torsional strain, which appears to be appreciably greater in **2** than in **1**. This is apparent in Fig. 4 and 5: protons at the bridging carbons of **1** adopt a fairly staggered conformation (H(11B)–C(11)–C(12)–H(12A): 47.6(3)°, H(12A)–C(12)–C(13)–H(13A): 41.3(4)°); whereas two protons flanking the central C–C bond in the *ansa* bridge of **2** are almost eclipsed in conformation (H(7B)–C(7)–C(8)–H(8B): 10.2(8)°, H(7AA)–C(7A)–C(8A)–H(8AA): 5.6(12)°). Torsional strain in **2** is expected to favour an exothermic ROP process, as for that of **1**. Whilst it is also possible that ROP of **2** is entropically favoured, (*i.e.* driven by greater conformational freedom in the polymer *vs.* monomer), the ROP of **1** is known to be significantly endoentropic^38^ (as is typical of most ROP processes)^49^ and the Δ*S*_ROP_ value is unlikely to be so different as to become exoentropic in the case of **2**.

**Table 1:** Selected distances (Å) and angles (°) in the *ansa* bridges of both **1** and **2**. (*α* = angle between the plane of each Cp ring, *β* = [180° – (Cp_cent_–Cp_ipso_–C_bridge_)] angle, *δ* = Cp_cent_–Ni–Cp′_cent_ angle).^35^,^54^ For a more extensive data set, please see Table S1

	Distances (Å)		Angles (°)	
Compound **2**			C(5)–C(6)–C(7)	113.9(6)
	C(6)–C(7)	1.531(11)	C(6)–C(7)–C(8)	112.4(7)
	C(7)–C(8)	1.515(13)	C(7)–C(8)–C(9)	115.3(7)
	C(8)–C(9)	1.548(11)	C(8)–C(9)–C(10)	114.6(6)
	H(7B)···H(8B)	2.15906(8)	H(7B)–C(7)–C(8)–H(8B)	10.2(8)
	C(6)–C(7A)	1.556(12)	C(5)–C(6)–C(7A)	112.3(8)
	C(7A)–C(8A)	1.51(2)	C(6)–C(7A)–C(8A)	116.2(10)
	C(8A)–C(9)	1.543(15)	C(7A)–C(8A)–C(9)	113.3(11)
	H(7AA)···H(8AA)	2.1464(1)	C(8A)–C(9)–C(10)	112.3(7)
			H(7AA)–C(7A)–C(8A)–H(8AA)	5.6(12)
Compound **1**			C(1)–C(11)–C(12)	115.8(3)
	H(12A)···H(13A)	2.26006(8)	C(11)–C(12)–C(13)	115.0(2)
	H(12A)···H(11B)	2.28442(8)	C(12)–C(13)–C(6)	115.28(19)
	C(11)–C(12)	1.529(5)	H(11B)–C(11)–C(12)–H(12A)	47.6(3)
	C(12)–C(13)	1.533(3)	H(12A)–C(12)–C(13)–H(13A)	41.3(4)

### DFT calculations of the enthalpy of ring-opening for **1** and **2**

**Scheme 2:**
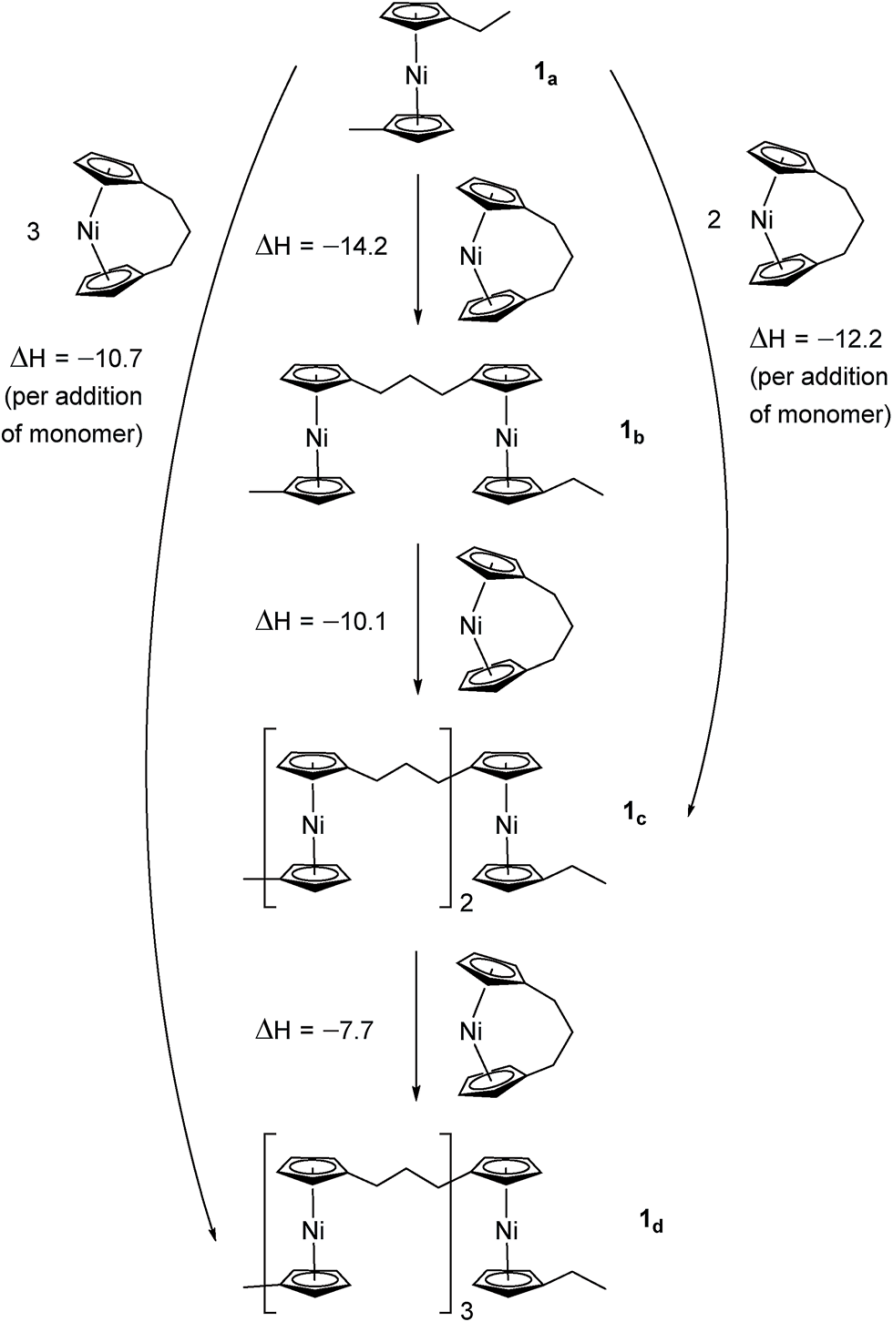
DFT model of ring-opening of monomer **1** to linear oligomers and values of enthalpic ring-opening (kJ mol^–1^).

**Scheme 3:**
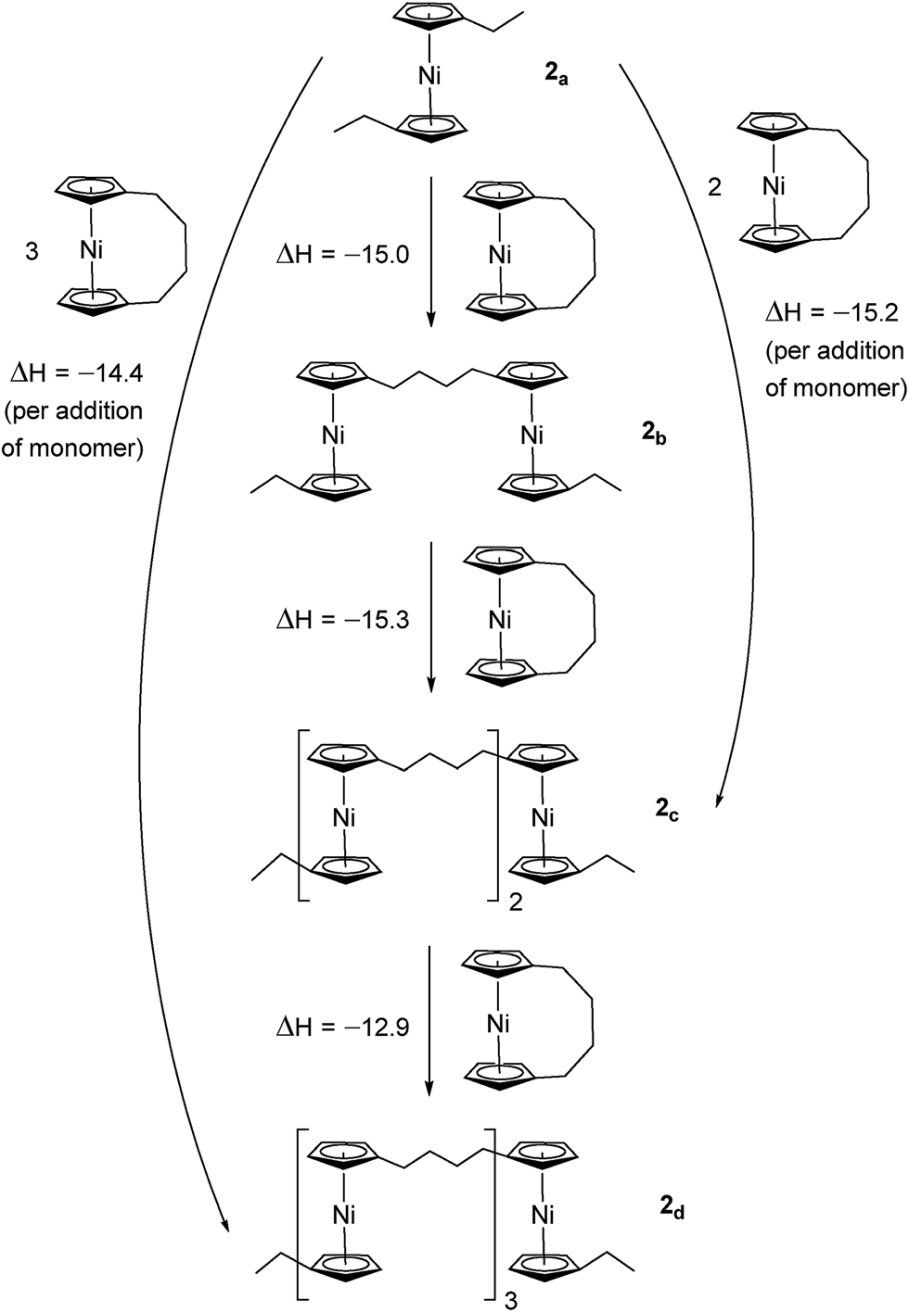
DFT model of ring-opening of monomer **2** to linear oligomers and values of enthalpic ring-opening (kJ mol^–1^).

Due to the lack of feasibility of investigating the enthalpic contribution to Δ*G*0ROP of monomer **2** experimentally, we explored the ROP of both **2** and **1** using DFT. Calculations were performed by modelling cyclic monomers and a series of linear oligomers (Scheme 2). Species **1_a_** is related to **1** by the addition of dihydrogen across the carbon backbone and serves as a model for polymer **8**. Addition of one monomer (**1**) to yield the linear dimer (**1_b_**) provided the enthalpic change upon ring-opening of **1**. Further addition of two successive monomers produced the linear trimer **1_c_**, then the linear tetramer **1_d_**. The enthalpy change upon ring-opening was estimated in each case and averaged over all linear oligomer models. As the calculated enthalpy of ring-opening, Δ*H*_RO_, for **1** (–11 ± 3 kJ mol^–1^) compares well to the experimental Δ*H*_ROP_ value (–10 kJ mol^–1^),^38^ the computational model was deemed appropriate. Thus, the model was applied in the same manner to ring-opened monomer **2_a_** (Scheme 3). The Δ*H*_RO_ value for **2** (–14 ± 2 kJ mol^–1^) is both negative and within error of that calculated for **1**. This suggests that the ROP of **2** is also exoenthalpic, driven by the release of ring-strain. Clearly this ring-strain is manifest not in the ring-tilt, but instead presumably in the torsional strain present in the *ansa* bridge. All computational details regarding Schemes 2 and 3 are provided in the ESI.†

In addition to polynickelocenes mentioned above, we recently reported the formation of poly(tetramethyl-disilylnickelocene) [Ni(η^5^-C_5_H_4_)_2_(SiMe_2_)_2_]_*n*_**16** from the disila-bridged [2]nickelocenophane **6** (Fig. 1).^54^ This monomer exhibits decreased tilt (*α* = 9.4(8)°)^34^*vs.***1** (*α* = 16.6(1)°). Despite this, ROP of **6** proceeds in pyridine to give predominantly insoluble polynickelocene, reminiscent of **12**, with no reversibility at 20 °C. We therefore applied a similar DFT model to **6** and the corresponding linear oligomers to determine the enthalpy of ring opening (Δ*H*_RO_ = –12 ± 3 kJ mol^–1^), which compares well to those determined for **1** and **2** (Scheme S1†).

As discussed, the insolubility of poly(nickelocenylbutylene) did not allow for the experimental determination of values of the entropy and enthalpy for ROP. Thus, other untilted monomers were targeted, that might undergo polymerisation to afford a soluble polymeric product. We considered the inclusion of a siloxane-based bridging element as it would be expected to significantly increase the solubility of a polynickelocene (syntheses of iron^55^ and titanium^56^ [*n*]metallocenophanes and [*n*]metalloarenophanes featuring siloxane-based bridges have been previously reported).

**Fig. 6:**
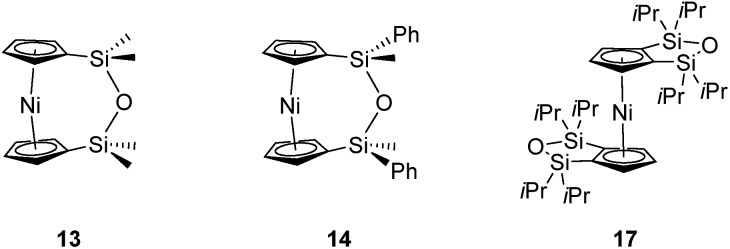
[*n*]Nickelocenophanes **13** and **14**, and substituted nickelocene **17**.

The synthesis of 1,1,3,3-tetramethyldisila-2-oxa[3]nickelocenophane **13** (Fig. 6) was conducted *via* a fly-trap procedure analogous to that previously employed for [*n*]nickelocenophanes **1**,^35^**2**,^54^ and **6**,^34^ involving reaction of the lithiated ligand Li_2_[(C_5_H_4_)_2_(SiMe_2_)_2_O] and nickel(ii) chloride.

**Fig. 7:**
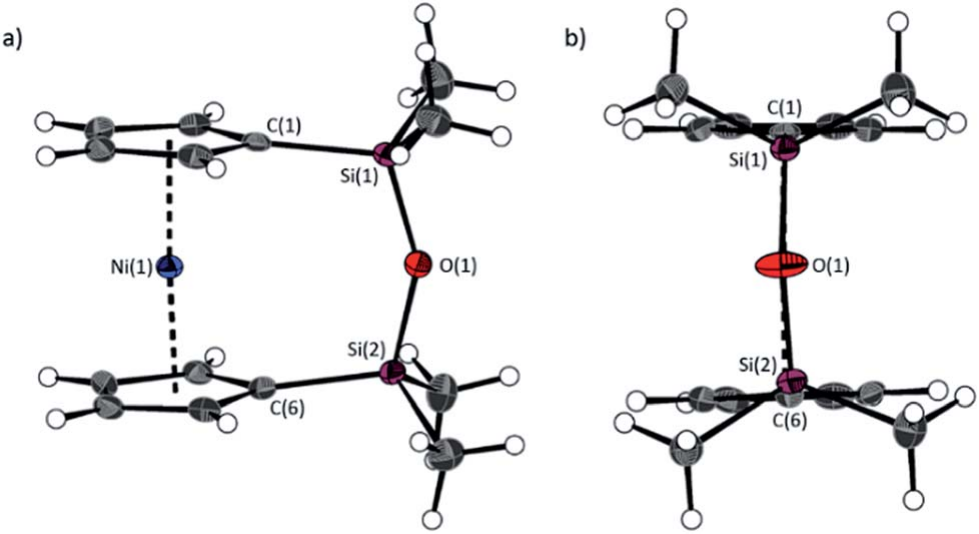
Two views (a and b) of the molecular structure of 1,1,3,3-tetramethyldisila-2-oxa[3]nickelocenophane **13**. Hydrogen atoms are pictured as spheres of arbitrary radii. Thermal ellipsoids displayed at the 50% probability level. Selected distances (Å) and angles (°): Ni(1)–Cp_cent_ = 1.8180(12)/1.8172(3), Si(1)–O(1) = 1.635(2), O(1)–Si(2) = 1.635(2), Ni(1)···O(1) distance = 3.5217(18), *α* = 3.76(10), *β* = 4.3(2)/5.0(2), *δ* = 177.20(2).

The fly-trap reaction yielded **13** as a green crystalline solid in moderate yield (43%). ^1^H NMR spectroscopy (C_6_D_6_) revealed resonances at –239 and –248 ppm which were assigned to the *α*- and *β*-Cp proton environments respectively, and at 8.58 ppm which was assigned to the SiMe_2_ protons in the *ansa* bridge (Fig. S1†). Crystallisation from hexanes yielded green crystals that allowed for full characterisation by X-ray diffraction. As expected, **13** exhibits a very small tilt angle, *α*, of 3.76(10)° (Fig. 7).

**Fig. 8:**
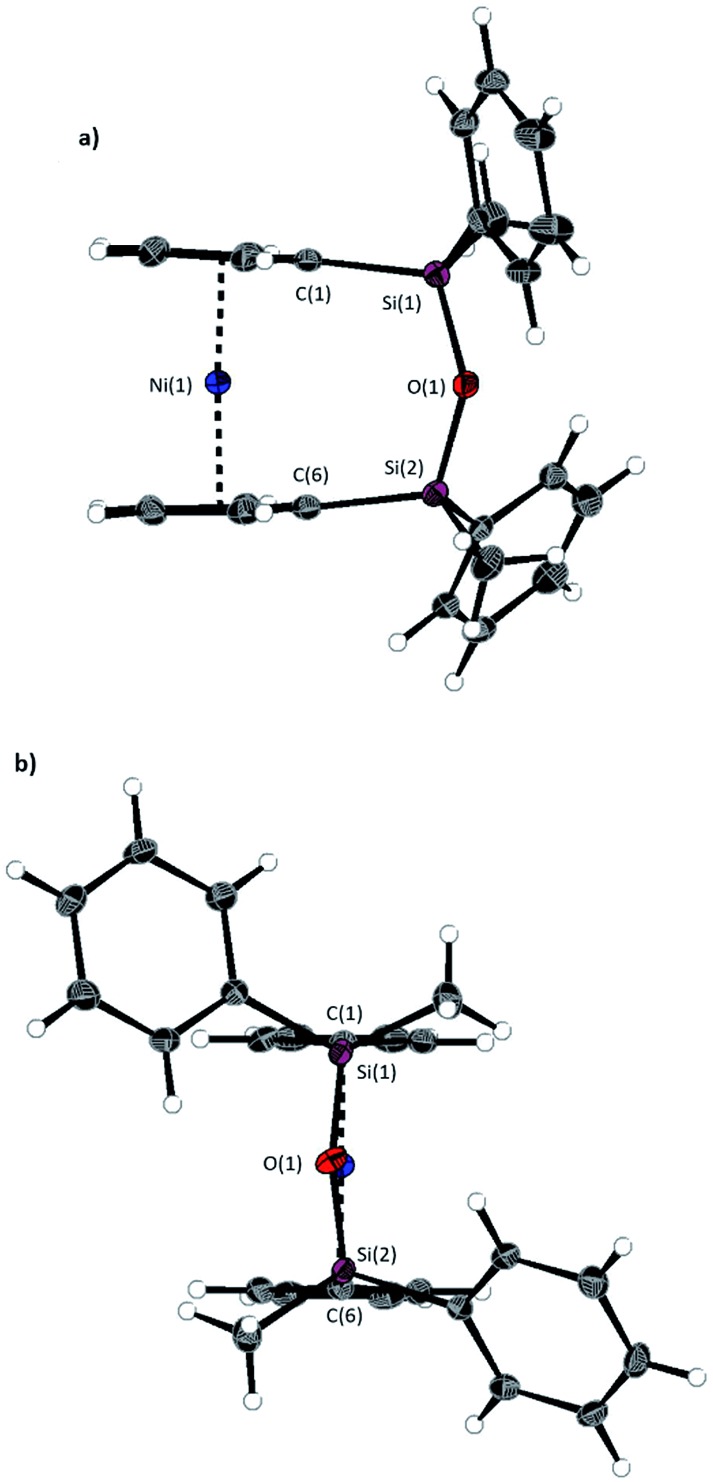
Two views (a and b) of the molecular structure of 1,3-dimethyl-1,3-diphenyldisila-2-oxa[3]nickelocenophane **14**. Hydrogen atoms are pictured as spheres of arbitrary radii. Thermal ellipsoids displayed at the 50% probability level. Selected distances (Å) and angles (°): Ni(1)–Cp_cent_ = 1.8188(9)/1.8165(9), Si(1)–O(1) = 1.6366(14), O(1)–Si(2) = 1.656(14), Ni(1)···O(1) distance = 3.5605(14), *α* = 4.18(8)°, *β* = 3.11(10)/5.04(10), *δ* = 177.07(4).

A second siloxane-bridged [3]nickelocenophane with sterically demanding groups at silicon was also synthesised. 1,3-Dimethyl-1,3-diphenyldisila-2-oxa[3]nickelocenophane **14** (Fig. 6) was similarly prepared by the reaction of the lithiated fly-trap ligand Li_2_[(C_5_H_4_)_2_(SiMePh)_2_O] with nickel dichloride. Unlike other [*n*]nickelocenophanes, the green solid produced did not sublime, presumably due to its increased molar mass. Subsequent recrystallisations from hexanes and *n*-pentane yielded green single crystals suitable for X-ray diffraction. Two diastereomers of **14** are feasible given the diastereomeric nature of the ligand, but all isolated crystalline material proved to be the *C*_2_-symmetric (*trans*) isomer (Fig. 8). Compound **14** displays a small tilt angle of 4.18(8)°, marginally larger than that of **13**. ^1^H NMR spectroscopy of the crystalline material revealed resonances at –237, –239, and –247 ppm assigned to the Cp proton environments, at 0.40 ppm assigned to the methyl protons in the *ansa* bridge, and between 7.58–8.54 ppm, attributed to the phenyl proton environments (Fig. S2†).

Interestingly, attempts to produce 1,1,3,3-tetraisopropyldisila-2-oxa[3]nickelocenophane were foiled due to an unforeseen ring closure reaction in the ligand synthesis (presumably due to the steric bulk of the isopropyl groups), leading to isolation of species **17** (Fig. 6) (further details regarding this species can be found in the ESI†).

ROP conditions (*d*_5_-pyridine, 0.79 M, 48 h, 20 °C) employed successfully for **1**, **2**, and **6** were then applied to siloxane-bridged **13**, but in contrast to tetracarba[4]nickelocenophane **2** (which also features a small tilt angle), exposure to pyridine did not induce polymerisation. This was demonstrated by ^1^H NMR spectroscopy, which showed only the presence of the unreacted monomer **13**. There was no evidence of reactivity even after attempting ROP at higher concentration (1.31 M) for an extended period of time (4 weeks). Several further attempts were made to induce polymerisation; firstly, by reaction of Na[C_5_H_5_] (both as an equimolar reagent and a substoichiometric initiator) with **13** under photolytic conditions (THF, 5 °C), but again no reaction occurred as evidenced by ^1^H NMR spectroscopy. Additionally, **13** was treated with equimolar 1,2-bis(diphenylphosphino)ethane (dppe), but this resulted in complete expulsion of the ligand framework and formation of [Ni(dppe)_2_] (observed by ^31^P NMR spectroscopy at 44.8 ppm). Whilst similar reactivity has been observed for **1**,^35^ the driving force was suggested to be the release of ring strain derived from ring tilt in **1**, which cannot be the case for **13**, because *α* is close to zero. To determine whether **13** would undergo thermal ROP, preliminary differential scanning calorimetry (DSC) experiments were performed. These indicated melt and crystallisation processes for **13**, in addition to a small exotherm feature at ∼184 °C (Fig. S4†). No new transitions were observed even after repeated heating cycles, and thus we concluded that thermal ring-opening would also prove unsuccessful.

In a similar manner to **13**, exposure of **14** to ROP conditions (*d*_5_-pyridine, 0.79 M, 48 h, 20 °C) did not induce polymerisation. The reaction was monitored by ^1^H NMR spectroscopy, which displayed only the presence of the monomer after one week. Unreacted starting material was obtained quantitatively *via* removal of the solvent *in vacuo*.

The cause for the stability of **13** and **14** to ROP, where the similarly untilted **2** undergoes facile polymerisation, may be deduced from the structural parameters. The longer Si–O bonds in the *ansa* bridge of **13** and **14** (1.630(3)/1.640(3) and 1.6356(14)/1.6366(14) Å, respectively) compared to the C–C bonds in that of **2** (ranging from 1.51(2)–1.556(12) Å), and the lack of substituents at the central bridge atom (oxygen), ensure that the torsional strain present in **2** does not occur in the siloxane-bridged species. Without this strain, and the ring-tilt that occurs in [*n*]nickelocenophanes **1** and **6**, there is no thermodynamic propensity for polymerisation. In addition, in [3]nickelocenophane **14** there do not appear to be any interactions between the phenyl groups in the *ansa* bridge, which could have introduced torsional strain.

We tested our computational model for the ring-opening of [*n*]nickelocenophanes to form linear oligomers with the siloxane-bridged [3]nickelocenophane **13**, as this species was resistant to ROP, and thus the calculated Δ*H*_RO_ should prove unfavorable. As expected, the computational model predicted the enthalpy of ring-opening to be small and positive (Δ*H*_RO_ = 5 ± 1 kJ mol^–1^) (Scheme S2†).

### Synthesis and ROP of methyltricarba[3]nickelocenophane **15**

To accompany our study on the ROP behaviour of [*n*]nickelocenophanes, we investigated the ROP of a methylated derivative of **1**, namely methyltricarba[3]nickelocenophane **15** (Fig. 3). The incorporation of bulky substituents in [*n*]metallocenophanes has been shown to significantly reduce their propensity to ROP,^45^ and therefore the formal replacement of H in **1** by the larger substituent, Me, in the bridge would generally be expected to make ROP less favourable.

**Fig. 9:**
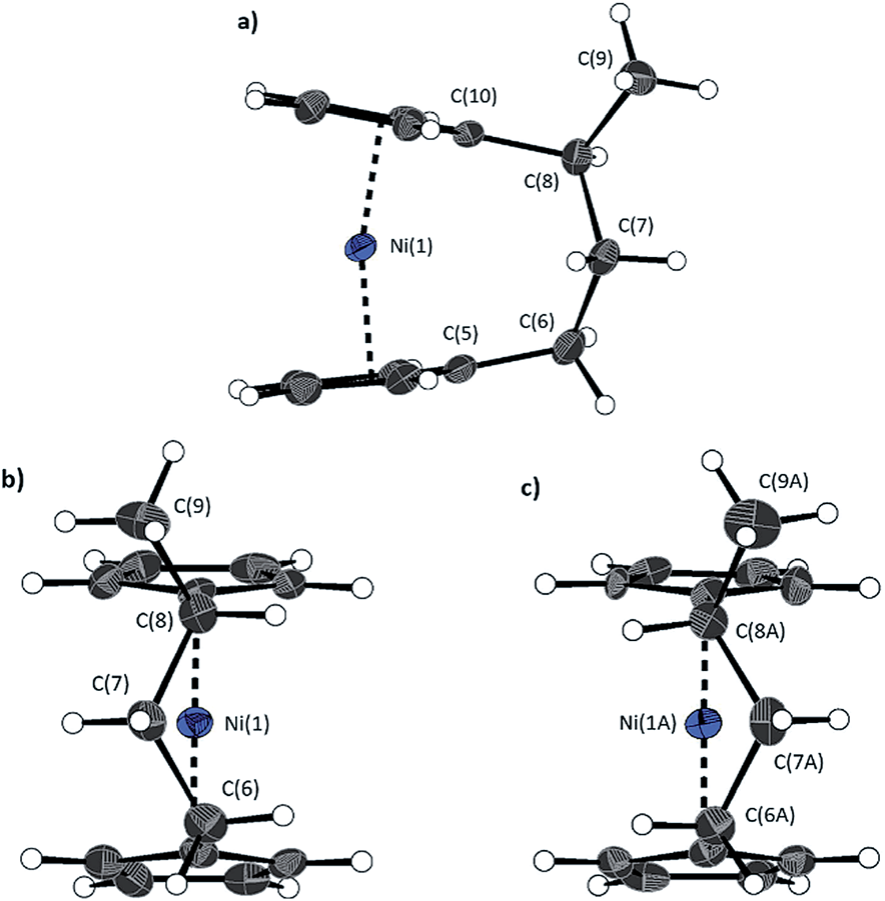
Three views of the molecular structure of **15**. Thermal ellipsoids displayed at the 50% probability level. Hydrogen atoms are pictured as spheres of arbitrary radii (and some have been omitted for clarity). (a) Compound **15** is disordered over two positions (highest relative occupancy structure (62%) is displayed). Alternate view of **15** displaying (b) major (62%) and (c) minor (38%) components. Selected distances (Å) and angles (°) for major component: Ni(1)–Cp_cent_ 1.795(6)/1.798(6), *α* = 16.3(2), *δ* = 166.0(3) (the angle *δ* is defined as the Cp_cent_–Ni–Cp_cent_ (Cp_cent_ = Cp centroid) angle).

[*n*]Nickelocenophane **15**, the first unsymmetrically substituted example, was synthesised as a racemate using the general method previously reported (reaction of lithiated ligand Li_2_{(C_5_H_4_)_2_(CH_2_)_2_[CH(CH_3_)]} with NiCl_2_).^35^,^54^ Sublimation and subsequent recrystallisation in hexanes afforded dark green crystals of **15** in 30% yield. ^1^H NMR spectroscopy of the crystalline material revealed overlapping resonances at –240, –243, and –247 ppm which were assigned to the four independent *α*-Cp protons, and overlapping resonances at –267 and –271 ppm which were assigned to the four independent *β*-Cp protons (see Fig. S5†), in line with previous assignments of Cp proton resonances in [*n*]nickelocenophanes where *β*-Cp protons appear at lower field.^34^,^38^ Resonances at –20.8 and –34.2 ppm were assigned to the *β*-H protons of the bridging carbons, the resonance at 14.1 ppm to protons in the *ansa* bridge methyl group, and as for species **1**, *α*-H protons could not be identified.^38^ X-ray crystallographic data confirmed the structure, but due to co-crystallisation of the two enantiomers (Fig. 9), the application of significant conformational restraints was necessary, so the following data should be treated with prudence: *α* was determined as 16.3(2)°, very similar to that of **1** (16.6(13)°).

**Scheme 4:**
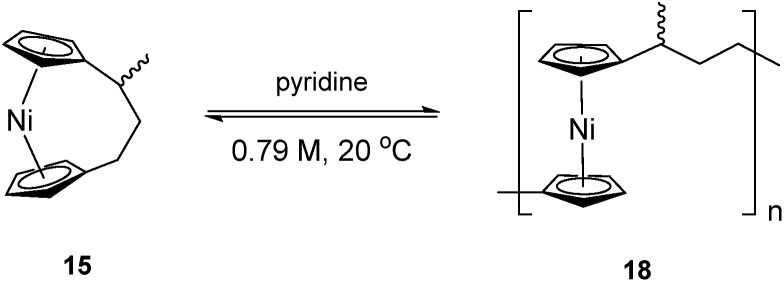
ROP of **15** to give **18** in pyridine (0.79 M).

As the ROP of **1** produces the soluble products **7_x_**/**8**, it was imagined that a polymeric product resulting from the ROP of **15** would exhibit similar solubility. The ROP of substituted [3]nickelocenophane **15** was investigated in *d*_5_-pyridine under the same conditions (0.79 M, 20 °C) as used for **1** (Scheme 4), and a gradual colour change from dark blue to green was observed. The reaction was followed by ^1^H NMR spectroscopy: after 24 h, alongside shifts assigned to Cp-ring protons in **15**, a broad singlet was observed at –251 ppm, which was assigned to Cp-ring protons in polymer **18**. Integration of Cp proton resonances in **15** and **18** indicated the presence of 41% polynickelocene **18**. After a further 24 h, a negligible increase in conversion to polymer (42%) was detected, which did not change significantly over 7 days. This suggests the presence of an equilibrium between monomer and polymer that is reached after 24 h. Attempted isolation of the resulting polymer *via* precipitation of the reaction solution (pyridine, 0.79 M, 48 h, 20 °C) into rapidly stirring hexanes at 20 °C was unsuccessful. However, when the reaction solution was precipitated into cold hexanes (–78 °C), a green solid was isolated. ^1^H NMR spectroscopy of polymeric **18** revealed resonances at 185, 180, and 147 ppm which were assigned to the *α*-protons in the carbon bridge, 22.1 ppm which was assigned to the methyl protons, and a broad resonance at –246 ppm which was assigned to the Cp protons (Fig. S6†). Analysis by MALDI-TOF mass spectrometry (of the reaction mixture prior to precipitation) revealed assignable peaks up to ∼8000 g mol^–1^, which corresponds to a degree of polymerisation (DP_*n*_) of ∼33 (Fig. S7†), but similar analysis of the isolated polymer was unsuccessful, presumably due to difficulties in ionising the higher molar mass fraction.

In order to prove the presence of a dynamic equilibrium, polynickelocene **18** was stirred in *d*_5_-pyridine (0.44 M) for 48 h at 20 °C. Resonances assigned to protons in both monomer **15** and polymer **18** were observable by ^1^H NMR spectroscopy, consistent with the reversible nature of the polymerisation, and the presence of a dynamic equilibrium. The percentage of polymer as a function of time was further investigated at a range of ROP concentrations (0.11–1.31 M, samples **18_a_–18_e_**, Table 2). ^1^H NMR spectroscopic analysis revealed an increase in the fraction of **18** with increased reaction concentration. Additionally, the yield of isolated polymer likely increases with concentration (isolation of polymer was only possible at 0.79 and 1.31 M). The discrepancy between the isolated polymer yield and that determined by integration of ^1^H NMR resonances is likely due to oligomeric species, which did not precipitate upon workup of the reaction solution.

**Table 2:** A comparison of yields of polymer **18** from ROP at various concentrations

Sample	Conc. (M)	% of **18** (^1^H NMR spectroscopy)			% Yield of **18** (20 °C)^*a*^	% Yield of **18** (–78 °C)^*a*^
		24 h	48 h	7 days		
**18_a_**	1.31	59.8	60.8	59.5	23	65
**18_b_**	0.79	41.1	41.8	42.2	^*b*^	29
**18_c_**	0.44	16.9	25.0	24.3	^*b*^	^*b*^
**18_d_**	0.22	3.17	14.3	14.3	^*b*^	^*b*^
**18_e_**	0.11	10.7	11.6	11.5	^*b*^	^*b*^

^*a*^Temperature at which the reaction work-up was performed.

^*b*^No polymer was isolated in this case.

The effect of reaction concentration on polymer molecular weight was investigated by dynamic light scattering (DLS). DLS experiments were performed in toluene, a marginal solvent for **18**, but one in which monomer/polymer equilibration is particularly slow.^38^ A small amount of insoluble material (presumably higher molar mass polymer) was observed during the preparation of DLS samples, which was greater in proportion for **18_a_** than for **18_b_**. As this material was removed *via* filtration before analysis by DLS, the molecular weights (estimated relative to a poly(ferrocenyldimethylsilane) calibrant)^57^ are likely lower than the real values (Table 3).

**Table 3:** DLS data for polymer **18** (isolated from ROP at various concentrations by precipitation into hexanes). All DLS samples were prepared in toluene at 1 mg mL^–1^

Sample	Conc. (M)	% Yield of **18**	*T* ^*b*^/(°C)	*R*_h_	Sigma	*M*_W_ ^*a*^
**18_a_**	1.31	23	20	7.42	1.69	82 700
		65	–78	9.35	3.04	126 000
**18_b_**	0.79	^*c*^	20	^*c*^	^*c*^	^*c*^
		29	–78	6.90	1.65	72 300

^*a*^Molecular weights are relative to calibration with poly(ferrocenyldimethylsilane) in toluene.^57^

^*b*^Temperature at which the reaction work-up was performed.

^*c*^No polymer was isolated in this case.

In comparison to the unsubstituted analogues **7_x_**/**8**, both isolated yields for polymer **18** and conversion determined by ^1^H NMR spectroscopy are significantly lower (Table 4). As these polymerisations exist as dynamic equilibria with oligomers and monomer, this suggests that the introduction of the methyl substituent to the *ansa* bridge causes the reaction equilibrium to favor [*n*]nickelocenophane relative to the case of **1**. Generally, ROP processes are sensitive to the size of any side groups and become thermodynamically unfavorable with sterically demanding substituents.^58^ However, studies of the ROP of sila[1]ferrocenophanes have shown that even those with bulky side groups form high molar mass polymer in very good yield.^45^,^59^ This is likely due to the high intrinsic strain present in the [1]ferrocenophanes (72 kJ mol^–1^ for dimethylsila[1]ferrocenophane).^19^,^43^ Due to the lower strain present in [*n*]nickelocenophanes the introduction of a substituent may cause a lower propensity to ROP due to non-bonding interactions with hydrogens in the polymer bridge. Interestingly, DLS results suggest that the isolated polynickelocene **18** is of higher molecular weight than the unsubstituted polynickelocene **7_x_**/**8** isolated in analogous conditions. This result, however, should be treated with caution as the presence of a methyl substituent may lead to improved polymer–solvent interactions which would give a concomitant increase in hydrodynamic radius.

**Table 4:** Comparison of yields of **7_x_**/**8**^38^ and **18**

Conc. (M)	% of **18** (^1^H NMR spectroscopy, 48 h)	% Yield of **18**^*a*^	% of **7_x_**/**8** (^1^H NMR spectroscopy, 48 h)	% Yield of **7_x_**/**8**^*a*^
1.31	60.8	23	78.9	62
0.79	41.8	^*b*^	69.0	30
0.44	25.0	^*b*^	48.5	21
0.22	14.3	^*b*^	20.6	^*b*^
0.11	11.6	^*b*^	4.3	^*b*^

^*a*^Isolated yield of polymer (precipitation into hexanes at 20 °C).

^*b*^No polymer isolated.

**Fig. 10:**
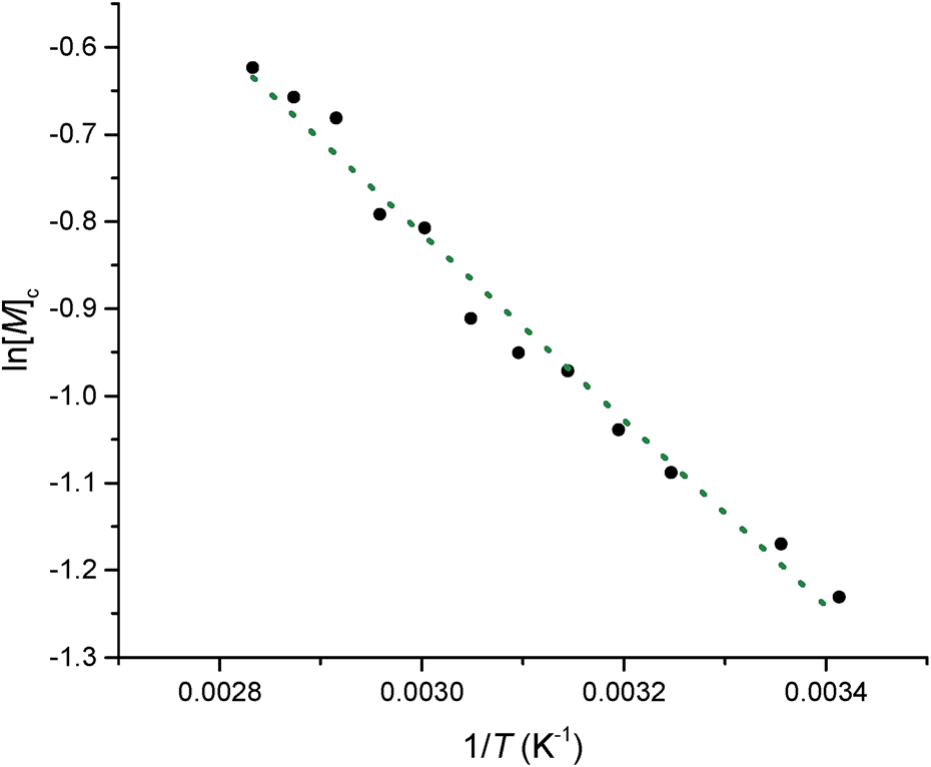
A Van't Hoff plot showing the relationship between log_e_ of the equilibrium monomer concentration and reciprocal temperature for the ROP of **15**.

Equilibrated monomer concentrations of **15** were fitted to eqn (1), an adaption of the Van't Hoff equation, to allow for determination of Δ*H*0ROP and Δ*S*0ROP (Fig. 10).1
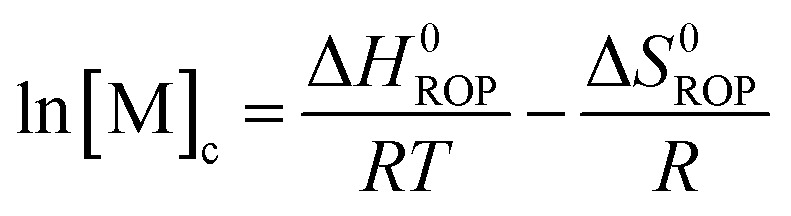
The magnitudes of Δ*H*0ROP (–8.9 kJ mol^–1^) and of Δ*S*0ROP (–20 J K^–1^ mol^–1^) for **15** were comparable to those determined for the polymerisation of **1** (Δ*H*0ROP = –10 kJ mol^–1^ and Δ*S*0ROP = –20 J K^–1^ mol^–1^).^38^ The marginally smaller value for Δ*H*0ROP of **15** compared to that for **1** is likely due to the introduction of the methyl substituent to the *ansa* bridge. The entropy of ROP in both cases is relatively small and negative; this presumably reflects the conformational flexibility of polynickelocenes, where the Cp rings exhibit free rotation about the Cp–Ni–Cp axis compared to the constrained [*n*]nickelocenophane structures, partly compensating for the loss of translational entropy associated with polymerisation. At room temperature (20 °C) the values of Δ*H*0ROP and *T*Δ*S*0ROP for the ROP of **15** are similar, resulting in a very small, favourable value for Δ*G*0ROP (–3.1 kJ mol^–1^), marginally reduced in magnitude in comparison to the ROP of **1** (Δ*G*0ROP = –4.0 kJ mol^–1^). The small value for Δ*G*0ROP explains the reversibility of the ROP of **15** to give **18**, and the reduced magnitude compared to that for **1** is consistent with the lower yields of **18** compared to **7_x_**/**8** (Table 4). By employing eqn (2),2
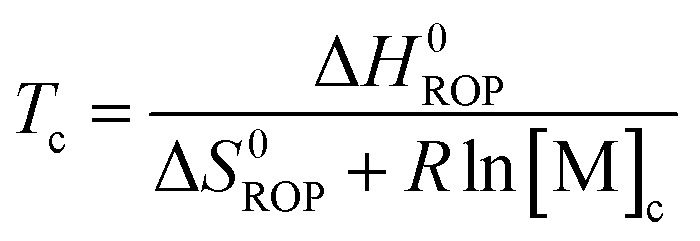

3
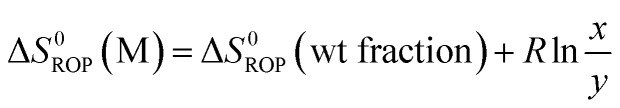
we were also able to determine a ‘ceiling temperature’ for the polymerisation of **15** at 0.79 M: *T*_c_ = 133 °C. The values of Δ*H*0ROP and Δ*S*0ROP, especially the latter, differ depending on the state of monomer and polymer, which in turn affects *T*_c_. Thus Δ*S*0ROP was recalculated using eqn (3), where *x* denotes a weight fraction (wt) corresponding to a monomer concentration of *y* mol L^–1^ (–6.8 J K^–1^ mol^–1^). This was applied to an adapted form of eqn (2), with molar concentration replaced by wt fraction, to determine a bulk *T*_c_, 1032 °C. This value is unsurprisingly similar to that reported for the ROP of **1** (1090 °C).^38^

## Conclusions

A series of low strain [*n*]nickelocenophanes have been studied with respect to their polymerisation behaviour. The recently reported, highly unusual ROP of untilted [Ni(η^5^-C_5_H_4_)_2_(CH_2_)_4_] (**2**) was investigated *via* DFT calculations. The exoenthalpic nature of the ROP is proposed to arise from the torsional strain present in the *ansa* bridge of **2**. Disiloxa-bridged [3]nickelocenophanes (**13** and **14**), which also exhibit negligible ring-tilt, were synthesised and found to be stable with respect to ROP under similar conditions. In these cases, DFT calculations revealed that ROP is unfavorable and no analogous torsional strain was evident from the molecular structures determined by single-crystal X-ray diffraction. Unsymmetrically substituted methyltricarba[3]nickelocenophane **15** was also synthesised and in this case the reversible ROP of this species to give **18** was studied in order to provide insight into the propensity of the monomer to undergo ROP. The addition of a methyl substituent to the *ansa* bridge appears to make only a small difference to enthalpy of polymerisation compared to that of the unsubstituted analogue (**15**: Δ*G*0ROP = –3.1 kJ mol^–1^*vs.***1**: Δ*G*0ROP = –4.0 kJ mol^–1^), but this is sufficient to significantly decrease the yields of **18** compared to **7_x_**/**8** under analogous conditions.

This study provides insight into a series of recent observations that have revealed that, although only low strain [*n*]nickelocenophanes are synthetically accessible due to the lability of the Ni–Cp bonds,^35^,^38^,^54^ the low kinetic barrier to ring-opening reactions can be used productively to allow ROP. The demonstration that torsional strain can drive ROP of metallocycles offers a useful design principle to exploit in future work on functional polynickelocenes and related materials.

## Experimental

### Materials and equipment

All reactions and product manipulations of molecular species were carried out under an inert atmosphere of dinitrogen or argon using standard Schlenk line or glovebox techniques (MBraun glovebox MB150G-B maintained at <0.1 ppm H_2_O and <0.1 ppm O_2_), unless otherwise stated. Dry hexanes, dichloromethane and toluene were obtained from a Grubbs-type solvent system employing alumina and supported copper columns.^60^ THF was distilled under dinitrogen from Na/benzophenone. Pyridine and *d*_5_-pyridine were purchased from Fluka and Sigma-Aldrich respectively and distilled from CaH_2_ prior to use. *d*_6_-Benzene was purchased from Sigma-Aldrich and stored over molecular sieves.^61^ Silica gel (for flash chromatography) was purchased from VWR and used as received. Celite 521 was obtained from Sigma-Aldrich and heated to 200 °C for 16 h prior to use. Sodium metal, dicyclopentadiene, anhydrous nickel(ii) chloride, 1,3-dichloro-1,1,3,3-tetramethyldisiloxane, 1,3-dichloro-1,3-dimethyl-1,3-diphenyldisiloxane, 1,3-dichloro-1,1,3,3-tetraisopropyldisiloxane, and 1,3-dibromobutane were used as supplied by Sigma-Aldrich. Anhydrous 1,3-dimethyl-3,4,5,6-tetrahydro-2(1*H*)-pyrimidinone (DMPU) was purchased from Sigma-Aldrich and degassed *via* three freeze–pump–thaw cycles. Na[C_5_H_5_]^62^ was prepared as described in the literature.

Electrospray ionisation (ESI) mass spectra were recorded using a cone potential of +150 V in a THF/toluene mixture on a Bruker Daltonics Apex IV Fourier transform ion cyclotron mass spectrometer.

Matrix assisted laser desorption/ionisation time of flight (MALDI-TOF) mass spectra were collected on a 4700 Proteomics Analyser (Applied Biosystems) equipped with a Nd:YAG laser, operating at 335 nm. Positive ion mass spectra were obtained in linear mode over a range of *m*/*z* values, and laser intensity was varied. Samples for analysis were prepared from a THF solution of the analyte (1 mg mL^–1^) and a THF solution of the matrix (10 mg mL^–1^), *trans*-2-[3-(4-tertbutylphenyl)-2-methyl-2-propenylidene]malononitrile (1 : 10 volume ratio) and drop-cast by micropipette into sample wells.

^1^H NMR spectra were recorded at ambient temperature on a VARIAN NMR 500 MHz spectrometer, and at variable temperature on a Bruker Advance III HD 500 Cryo spectrometer. All spectra are reported relative to external TMS and are referenced to the most downfield residual solvent resonance (*d*_5_-pyridine: *δ*_H_ 8.74 ppm, C_6_D_6_: *δ*_H_ 7.16 ppm). In all ^1^H NMR spectra of paramagnetic [*n*]nickelocenophane and polynickelocene species, backward linear prediction from 0 to 15 data points was employed to remove baseline distortion, phase correction was addressed manually, and a Bernstein polynomial fit was applied (polynomial order = 10). ^1^H NMR spectra were collected between +310 and –310 ppm to observe signals at both low and high field (number of scans = 2048, receiver gain = 50, relaxation delay = 0.10 s and acquisition time = 0.84 s).

Dynamic Light Scattering (DLS) experiments were performed to determine hydrodynamic radii of polymer solutions. Samples (2 mL) of different polymer concentrations (1 and 2 mg mL^–1^) in toluene were filtered through a 0.45 μm membrane filter into an optical glass cuvette (10.0 mm path length). The measurements were performed on a Malvern Instruments Zetasizer Nano S using a 5 mW He–Ne laser (633 nm) at 20 °C. The correlation function was acquired in real time and analysed with a function capable of modelling multiple exponentials. This process enabled the diffusion coefficients for the component particles to be extracted, and these were subsequently expressed as effective hydrodynamic radius, by volume, using the Stokes–Einstein relationship.

Photoirradiation experiments were carried out using Pyrex-glass-filtered emission (*λ* > 310 nm) from a 125 W high pressure Hg vapour lamp (Photochemical Reactors Ltd.).

Elemental analyses were carried out by Elemental Microanalysis Ltd using the Dumas combustion method.

Single crystal X-ray diffraction experiments were carried out at 100 K on a Bruker APEX II diffractometer using Mo Kα radiation (*λ* = 0.71073 Å). Data collections were performed using a CCD area detector from a single crystal mounted on a glass fibre. Intensities were integrated,^63^ from several series of exposures measuring 0.5° in *ω* or *φ*. Absorption corrections were based on equivalent reflections using SADABS.^64^ The structures were solved using SHELXS and refined against all *F*_o_^2^ data with hydrogen atoms located geometrically and refined using a riding model in SHELXL.^65^ Crystallographic details are provided in the ESI.†

For detailed computational methods and calculated molecular coordinates see the ESI.†

#### Synthesis of 1,1,3,3-tetramethyldisila-2-oxa[3]nickelocenophane (**13**)

Na[C_5_H_5_] (10.0 g, 0.114 mol) was dissolved in THF (100 mL) and cooled to –78 °C. 1,3-Dichloro-1,1,3,3-tetramethyldisiloxane (10.6 mL, 0.054 mol) was also dissolved in THF (25 mL) and added to the Na[C_5_H_5_] solution dropwise over 30 min. The mixture was stirred and allowed to warm to room temperature over 16 h. H_2_O (50 mL) was added to the pale pink suspension, resulting in a colour change to yellow/brown, and the organic phase was extracted with Et_2_O. The aqueous phase was washed with Et_2_O (3 × 20 mL) and the organic phase was washed with H_2_O (10 × 20 mL) to remove all remaining cyclopentadiene. The organic solution was dried with MgSO_4_ and all solvent removed to yield a yellow oil. This product was distilled (100 °C, 7.0 × 10^–2^ mbar) to yield 11.6 g (0.044 mol) of a colourless oil, (C_5_H_5_)_2_[(SiMe_2_)_2_O], which was dissolved in dry hexanes (250 mL) and cooled to –78 °C. *n*BuLi (1.60 M hexane solution, 70.0 mL, 0.114 mol) was added dropwise over 10 min, and the resulting solution was stirred and allowed to warm to room temperature over 16 h. The colourless suspension was filtered to collect the solid, which was washed with hexanes (8 × 20 mL) to remove excess *n*BuLi and dried under vacuum to yield Li_2_[(C_5_H_4_)_2_(SiMe_2_)_2_O] as a colourless, free-flowing solid (10.8 g, 0.039 mol) in 73% yield.

The fly-trap ligand Li_2_[(C_5_H_4_)_2_(SiMe_2_)_2_O] (3.00 g, 10.9 mmol) and NiCl_2_ (1.55 g, 12.0 mmol) were thoroughly mixed in the absence of solvent and cooled to –78 °C. Dry and degassed THF (200 mL) pre-cooled to –78 °C was then added rapidly *via* cannula. The reaction mixture was stirred and allowed to warm to room temperature over a period of 16 h. After evaporation of the solvent under reduced pressure, the green residue was extracted with hexanes to give a dark green solution which was filtered through Celite (1′′ × 4′′). Again, all volatiles were removed *in vacuo* and the resulting green solid was purified by sublimation (40 °C/–78 °C, 5.0 × 10^–2^ mbar) and subsequent recrystallisation from hexanes at –40 °C to afford dark green crystals of **13** suitable for X-ray crystallographic analysis. Yield: 1.50 g (4.70 mmol, 43%). ^1^H NMR (500 MHz, C_6_D_6_): *δ* [peak width at half height] (ppm) 8.58 [18 Hz] (br s, C_5_H_4_–Si(C*H*_3_)_2_), –239.4 [906 Hz] (br s, C_5_H_4_), –248.1 [969 Hz] (br s, C_5_H_4_). ESI-MS (positive ion mode, 1,2-difluorobenzene): *m*/*z* 318.0401 [Ni(η^5^-C_5_H_4_)_2_(SiMe_2_)_2_O]^+^. Anal. calcd for C_14_H_20_NiOSi_2_: C 52.68%, H 6.32% found: C 53.35%, H 6.35%.

#### Attempted ROP of **13** in pyridine

1,1,3,3-Tetramethyldisila-2-oxa[3]nickelocenophane **13** (126 mg, 0.395 mmol) was dissolved in *d*_5_-pyridine (0.50 mL) to afford a 0.79 M solution, and stirred at room temperature for 32 h. No colour change was observed. ^1^H NMR spectroscopy (500 MHz, *d*_5_-pyridine) revealed no change. The procedure was repeated at higher concentration (1.31 M) and again, no change was observed after 32 h by ^1^H NMR spectroscopy. After 1 month, no further change was observed by ^1^H NMR spectroscopy.

#### Synthesis of 1,3-dimethyl-1,3-diphenyldisila-2-oxa[3]nickelocenophane (**14**)

Na[C_5_H_5_] (5.7 g, 0.064 mol) was dissolved in THF (70 mL) and cooled to –78 °C. 1,3-Dichloro-1,3-dimethyl-1,3-diphenyldisiloxane (8.7 mL, 0.031 mol) was also dissolved in THF (15 mL) and added to the Na[C_5_H_5_] solution dropwise over 30 min. The mixture was stirred and allowed to warm to room temperature over 16 h. H_2_O (50 mL) was added to the pale pink suspension, resulting in a colour change to pale orange, and the organic phase was extracted with Et_2_O. The aqueous phase was washed with Et_2_O (3 × 20 mL) and the organic phase was washed with H_2_O (10 × 20 mL) to remove all remaining cyclopentadiene. The organic solution was dried with MgSO_4_ and all solvent removed to yield an orange oil. This product was distilled (140 °C, 7.0 × 10^–2^ mbar) to yield 6.49 g (0.017 mol) of a colourless oil, (C_5_H_5_)_2_[(SiMePh)_2_O], which was dissolved in dry hexanes (150 mL) and cooled to –78 °C. *n*BuLi (1.6 M hexane solution; 26 mL, 0.042 mol) was added dropwise over 10 min, and the resulting solution was stirred and allowed to warm to room temperature over 16 h. The colourless suspension was filtered to collect the solid, which was washed with hexanes (8 × 20 mL) to remove excess *n*BuLi and dried under vacuum to yield Li_2_[(C_5_H_4_)_2_(SiMePh)_2_O] as a colourless, free-flowing solid (5.2 g, 0.013 mol) in 42% yield.

The fly-trap ligand Li_2_[(C_5_H_4_)_2_(SiMePh)_2_O] (1.00 g, 2.51 mmol) and NiCl_2_ (0.340 g, 2.63 mmol) were thoroughly mixed in the absence of solvent and cooled to –78 °C. Dry and degassed THF (100 mL) pre-cooled to –78 °C was then added rapidly *via* cannula. The reaction mixture was stirred and allowed to warm to room temperature over a period of 16 h. After evaporation of the solvent under reduced pressure, the green residue was extracted with hexanes to give a dark green solution which was filtered through Celite (1′′ × 4′′). Again, all volatiles were removed *in vacuo* and the resulting green solid was recrystallised in hexanes at –40 °C to afford a green solid and a dark green solution. The solid was separated from the solution and then recrystallised in *n*-pentane at –40 °C to give green crystals of **14** suitable for X-ray crystallographic analysis. Yield: 0.18 g (0.41 mmol, 16%). ^1^H NMR (500 MHz, C_6_D_6_): *δ* [peak width at half height] (ppm) 8.54–7.58 (m, C_5_H_4_–Si(C_6_*H*_5_)_2_), 0.40 [3 Hz] (s, C_5_H_4_–Si(C*H*_3_)), –237.2 [887 Hz] (br s, C_5_H_4_), –239.3 [891 Hz] (br s, C_5_H_4_), and –247.2 [1008 Hz] (br s, C_5_H_4_). ESI-MS (positive ion mode, toluene): *m*/*z* 442.0718 [Ni(η^5^-C_5_H_4_)_2_(SiMePh)_2_O]^+^. Anal. calcd for C_24_H_24_NiOSi_2_: C 65.02%, H 5.46% found: C 64.09%, H 5.67%.

#### Attempted ROP of **14** in pyridine

1,3-Dimethyl-1,3-diphenyldisila-2-oxa[3]nickelocenophane **14** (126 mg, 0.390 mmol) was dissolved in *d*_5_-pyridine (0.50 mL) to afford a 1.31 M solution, and stirred at room temperature for 1 week. ^1^H NMR spectroscopy (500 MHz, *d*_5_-pyridine) revealed no change.

#### Synthesis of 1-methyltricarba[3]nickelocenophane (**15**)

Na[C_5_H_5_] (11.4 g, 0.129 mol) was dissolved in THF (125 mL) and cooled to –78 °C before 1,3-dimethyl-3,4,5,6-tetrahydro-2-pyrimidinone (40 mL) was added. 1,3-Dibromobutane (5.6 mL, 0.046 mol) was also dissolved in THF (50 mL) and added to the Na[C_5_H_5_] solution dropwise over 30 min. The mixture was stirred at –78 °C for 1 h, then at 0 °C for a further 2 h. H_2_O (100 mL) was added to the pale pink suspension, and the organic phase was extracted with Et_2_O. The aqueous phase was washed with Et_2_O (3 × 200 mL) and the organic phase was washed with H_2_O (10 × 100 mL) to remove all remaining cyclopentadiene. The organic solution was dried with MgSO_4_ and flushed through a silica column (1′′ × 6′′) with a DCM eluent. All solvent was removed to yield 6.8 g (0.036 mol) of (C_5_H_5_)_2_[(CH_2_)_2_CH(CH_3_)] as a pale yellow oil. This product was dissolved in dry hexanes (200 mL) and cooled to –78 °C. *n*BuLi (1.6 M hexane solution; 57 mL, 0.091 mol) was added dropwise over 10 min, and the resulting solution was stirred and allowed to warm to room temperature over 16 h. The suspension was filtered to collect the solid, which was washed with hexanes (5 × 50 mL) to remove excess *n*BuLi and dried under vacuum to yield Li_2_{(C_5_H_4_)_2_(CH_2_)_2_[CH(CH_3_)]} as a colorless, free-flowing solid (5.7 g, 0.029 mol) in 62% yield.

The fly-trap ligand Li_2_{(C_5_H_4_)_2_(CH_2_)_2_[CH(CH_3_)]} (2.00 g, 10.1 mmol) and NiCl_2_ (1.37 g, 10.6 mmol) were thoroughly mixed in the absence of solvent and cooled to –78 °C. Dry and degassed THF (250 mL) pre-cooled to –78 °C was then added rapidly *via* cannula. The reaction mixture was stirred and allowed to warm up to room temperature over a period of 16 h. After evaporation of the solvent under reduced pressure, the green residue was extracted with hexanes to give a dark green solution which was filtered through Celite (1′′ × 4′′). Again, all volatiles were removed *in vacuo* and the resulting green solid was purified by sublimation (40 °C/–78 °C, 1.0 × 10^–2^ mbar) and subsequent recrystallisation from hexanes at –40 °C to afford dark green crystals of **15**. Yield: 0.73 g (3.0 mmol, 30%). ^1^H NMR (500 MHz, C_6_D_6_): *δ* [peak width at half height] (ppm) 14.14 [95 Hz] (s, –C*H*_3_), –20.8 [232 Hz] (s, C_5_H_4_–CH_2_–C*H*_2_), –34.2 [194 Hz] (s, C_5_H_4_–CH_2_–C*H*_2_), –240.4 [900 Hz] (br s, *α*-C_5_H_4_), –242.9 [887 Hz] (br s, *α*-C_5_H_4_), –246.5 [1012 Hz] (br s, *α*-C_5_H_4_), –266.9 [1125 Hz] (br s, *β*-C_5_H_4_), –270.8 [1579 Hz] (br s, *β*-C_5_H_4_). The lack of a signal corresponding to the C_5_H_4_–C*H*_2_–CH_2_ protons is consistent with similar *ansa* [*n*]nickelocenophanes. ESI-MS (positive ion mode, toluene): *m*/*z* 242.0605 [Ni(η^5^-C_5_H_4_)_2_(CH_2_)_2_(CH(CH_3_))]^+^. Anal. calcd for C_14_H_16_Ni: C, 69.21; H, 6.64; found: C, 70.04; H, 6.56.

#### Concentration dependency of the ROP of **15**

All polymerisations were carried out in an analogous manner: **15** was dissolved in *d*_5_-pyridine (0.50 mL) to afford a dark blue solution. The solution was stirred at room temperature for 24 h, where at low concentrations, no colour change was observed, and at higher concentrations a colour change from dark blue to green was evident. ^1^H NMR spectra were recorded after 24 h, 48 h, and 7 days.

A green solid was isolated in the cases of **18_a_** and **18_b_***via* precipitation of the polymerisation solution into rapidly stirred –78 °C hexanes (30 mL). The precipitate was separated, dissolved in a minimum volume of THF (0.5 mL) and precipitated into –78 °C hexanes again (30 mL). The solid was isolated and dried *in vacuo* to yield green polymeric material, **18**. In the case of **18_a_** (1.31 M), polymeric material was also isolated from precipitation into 20 °C hexanes. ^1^H NMR (500 MHz, C_6_D_6_): *δ* [peak width at half height] (ppm) 185.4 [661 Hz] (br s, C_5_H_4_–C*H*(CH_3_)–CH_2_), 179.6 [1087 Hz] (br s, C_5_H_4_–C*H*_2_–CH_2_), 146.7 [985 Hz] (br s, C_5_H_4_–C*H*_2_–CH_2_), 22.1 (m, CH(C*H*_3_)), –246.3 [1790 Hz] (br s, C_5_H_4_). MALDI-TOF MS (linear, +mode): 2128 (*n* = 8), 2371 (*n* = 9), 2614 (*n* = 10), 2857 (*n* = 11), 3100 (*n* = 12), 3343 (*n* = 13), 3586 (*n* = 14), 3829 (*n* = 15), 4072 (*n* = 16), 4315 (*n* = 17), 4558 (*n* = 18), 4801 (*n* = 19), 5044 (*n* = 20), 5287 (*n* = 21), 5530 (*n* = 22), 5773 (*n* = 23), 6016 (*n* = 24), 6259 (*n* = 25), 6502 (*n* = 26), 6744 (*n* = 27), 6987 (*n* = 28), 7230 (*n* = 29), 7473 (*n* = 30), 7716 (*n* = 31), 7959 (*n* = 32) (for assignment of end groups see Fig. S7†). Further characterisation can be found for specific concentrations in Tables 2 and 3.

#### Temperature dependency of the ROP of **15**

All polymerisations were carried out in an analogous manner: **15** (96.0 mg) was dissolved in *d*_5_-pyridine (0.50 mL) at room temperature to afford a dark blue solution (0.79 M). The solution was equilibrated at constant temperature (ranging from –5 to 55 °C) for 48 h, and then analysed *in situ* by ^1^H NMR spectroscopy. At higher temperatures, no colour change was observed, and at lower temperatures a colour change from dark blue to green was evident.

#### Depolymerisation of **18**

Polynickelocene **18** (53 mg, 0.22 mmol) was stirred in *d*_5_-pyridine (0.50 mL) for 48 h at 20 °C to yield a mixture of monomer and polymer. ^1^H NMR spectroscopy displayed resonances in line with those reported above for **15** and **18**.

## Conflicts of interest

There are no conflicts to declare.

## Supplementary Material

Supplementary information

Crystal structure data
