# Supplementary material for: Role of torsional strain in the ring-opening polymerisation of low strain [n]nickelocenophanes
Source: Chem Sci. 2019 Sep 10;10(42):9841–52. doi: 10.1039/c9sc02624j (PMC6977548; doi:10.1039/c9sc02624j)
Supplement: Supplementary file 1 [file SC-010-C9SC02624J-s001.pdf]

Electronic Supplementary Material (ESI) for Chemical Science.
This journal is © The Royal Society of Chemistry 2019

Role of Torsional Strain in the Ring-Opening Polymerisation of Low Strain [*n*]Nickelocenophanes

Rebecca A. Musgrave,[†,1] Rebekah L. N. Hailes,[†,1] Vincent T. Annibale,[1] and Ian Manners*[1,2]

[†]These authors contributed equally.

[1]School of Chemistry, University of Bristol, Bristol BS8 1TS, United Kingdom

[2] Department of Chemistry, University of Victoria, Victoria, BC V8W 3V6, Canada

[*]e-mail: imanners@uvic.ca

Supporting Information

i. Computational Methods

DFT Calculations regarding the ring-opening propensity of monomers **1**, **2**, **6**, and **13** (Tables S1 and S2) were carried out using Gaussian 09,[1] using the B3LYP functional and the –D2 dispersion correction with keyword iop(3/124=3) of Grimme et al.[2-8] as implemented in Gaussian. For geometry optimisation, the standard Stuttgart/Dresden ECP and associated basis set were used on nickel,[9] and the 6-31G(d) basis set on carbon and hydrogen (with the 5 spherical harmonic d functions on C).[10-11] This basis set combination is denoted as BS1. Frequency calculations were performed at the same level of theory (B3LYP/BS1) and used to confirm stationary points as minima, compute zero-point energy and derive gas-phase values for thermal and entropic corrections at 298.15 K. Thermodynamic data are provided in Tables S2 and S3, and atomic coordinates are provided in Section v.

The computed Gibbs energies within Gaussian use standard conditions which correspond to an ideal gas with pressure of 1 atm at 298.15 K. These were converted to Gibbs energies which instead correspond to the solution phase standard state of 1 M, by adding a "free energy

correction term" equal to RTln(V_g/V_s) to the free energy of each species.[12] In this equation, R is the ideal gas constant (8.314 J K^{-1} mol^{-1}), T is the absolute temperature (K), V_g is the volume occupied by one mole of ideal gas at 298.15 K, and V_s is the volume occupied by one mole of species in a standard solution of concentration 1 M (i.e. 1 dm^3).

For the basis set correction, a single-point energy was computed, changing the 6-31G(d) basis set of only C and H to the larger 6-311G(d) basis set to obtain improved energy values (this basis set is denoted as BS2). For the solvation correction, the standard IEF-SCRF continuum solvent method was used in single-point calculations with the B3LYP-D2/SDD,6-31G(d) method used for geometry optimisation (BS1), with continuum parameters designed to describe pyridine solvent ($\varepsilon = 12.98$).[13-14]

The difference between the vacuum and continuum energy was added to the small basis vacuum free energy:

$$G(BS2) = G(BS1) + (E(BS2) - E(BS1))$$

and also added to the large basis single-point energy:

$$\text{“E(BS2)+SCRF”} = SCRF + (E(BS2) - E(BS1))$$

The difference between the vacuum free energy and vacuum electronic energy was further added to this to yield approximate solvent-phase accurate energies:

$$\text{“E(BS2)+SCRF+G(BS1)”} = SCRF + G(BS2) - E(BS1)$$

Although the continuum energy component cannot be resolved into specific enthalpic or entropic contributions, the small basis vacuum electronic energy (E(BS1) of all species of the tricarba-, tetracarba-, disila- and siloxane-bridged systems does not differ significantly from the continuum energy (SCRF), and we can therefore assume that SCRF − E(BS1) = 0. It is unlikely that the enthalpic component within the continuum electronic energy are large enough to alter H(BS2) and significantly, and therefore difference between the vacuum electronic

energies of the two basis sets can be added to the sum of electronic and thermal enthalpies to give a rough estimate of the solution phase accurate enthalpy:

$$H(BS2) = H(BS1) + E(BS2) - E(BS1)$$

Conformers of all complexes (**1**–**1d**, **2**–**2d**, **6**–**6c**, **13**–**13c**) were investigated via molecular mechanics GMMX conformer searches using the MMX forcefield as implemented in PCModel,[15] and where applicable, multiple conformers were optimised in Gaussian (as described above) to confirm the lowest energy structures.

ii. Synthesis, characterisation and attempted polymerisation of 17

Interestingly, attempts to produce 1,1,3,3-tetraisopropyldisila-2-oxa[3]nickelocenophane were foiled due to an unforeseen ring closure reaction in the ligand formation step (presumably due to the steric bulk of the isopropyl groups), leading to isolation of species **17**. The lithiated bulky Cp ligand was reacted with nickel dichloride to produce the unexpected species 1,1′,2,2′-bis(tetraisopropyldisiloxa)nickelocene, **17** (Figure 6), as a green crystalline solid in 41% yield. X-ray crystallography confirmed the presence of the substituted nickelocene (Figure S11). ^{1}H NMR resonances at −199.5 and −234.8 ppm were assigned to protons in Cp environments, and a series of resonances between 2.23 and 6.69 ppm to protons of the isopropyl groups (Figure S3). An attempt to produce a cross-linked polynickelocene from the substituted nickelocene, via addition of sBuLi and subsequent ring-opening of the Si_2OC_2 ring, were unsuccessful. A colour change from green to brown was observed but products were unidentifiable by ^{1}H NMR spectroscopy and ESI mass spectrometry. Although the synthesis of 1,1,3,3-tetraisopropyldisila-2-oxa[3]nickelocenophane was not successful, nor the ring-opening reaction of **17**, a facile route to lithiated, substituted Cp rings has been discovered, which may prove useful in other areas of metallocene chemistry.

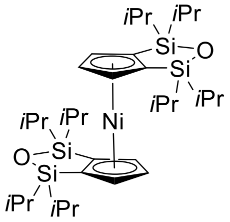

17

Synthesis of 1,1′,2,2′-Bis(tetraisopropyldisiloxa)nickelocene (17). Na[C_5H_5] (7.9 g, 0.089 mol) was dissolved in THF (100 mL) and cooled to −78 °C. 1,3-Dichloro-1,1,3,3-tetraisopropyldisiloxane (10 mL, 0.041 mol) was also dissolved in THF (20 mL) and added to the Na[C_5H_5] solution dropwise over 30 min. The mixture was stirred and allowed to warm to room temperature over 16 h. H_2O (50 mL) was added to the pale pink suspension, resulting in a colour change to pale orange, and the organic phase was extracted with Et_2O. The aqueous phase was washed with Et_2O (3 × 20 mL) and the organic phase was washed with H_2O (10 × 20 mL) to remove all remaining cyclopentadiene. The organic solution was dried with $MgSO_4$ and all solvent removed to yield an orange oil. This product was distilled (150 °C, 7.0 × 10^{-2} mbar) to yield 7.07 g (0.022 mol) of a colorless oil, (C_5H_4)[(Si*i*Pr_2)O(Si*i*Pr_2)], which was dissolved in dry hexanes (100 mL) and cooled to −78 °C. *n*BuLi (1.6 M hexane solution; 27

mL, 0.043 mol) was added dropwise over 10 min, and the resulting solution was stirred and allowed to warm to room temperature over 16 h. The colorless suspension was filtered to collect the solid, which was washed with hexanes (8 × 20 mL) to remove excess *n*BuLi and dried under vacuum to yield Li[(C_5H_3)(Si*i*Pr_2)$_2$O] as a colorless, free-flowing solid (5.78 g, 0.018 mol) in 45% yield.

The ligand Li[(C_5H_3)(Si*i*Pr_2)$_2$O] (1.0 g, 3.18 mmol) and $NiCl_2$ (0.21 g, 1.59 mmol) were thoroughly mixed in the absence of solvent and cooled to −78 °C. Dry and degassed THF (90 mL) pre-cooled to −78 °C was then added rapidly via cannula. The reaction mixture was stirred and allowed to warm to room temperature over a period of 16 h. After evaporation of the solvent under reduced pressure, the green/brown residue was extracted with hexanes to give a dark green/brown solution which was filtered through Celite (1" × 4"). Again, all volatiles were removed in vacuo and the resulting green solid was recrystallised from hexanes at −40 °C to afford light green crystals of **17** suitable for X-ray crystallographic analysis. Yield: 0.44 g (0.65 mmol, 41%). ^{1}H NMR (500 MHz, C_6D_6): δ [peak width at half height] (ppm) 6.68 (s, C_5H_4–SiC*H*), 5.43 (s, C_5H_4–SiC(C*H*$_3$)$_2$), 5.09 (s, C_5H_4–SiC(C*H*$_3$)$_2$), 12 (s, C_5H_4–SiC*H*), 2.59 (s, C_5H_4–SiC(C*H*$_3$)$_2$), 2.23 (s, C_5H_4–SiC(C*H*$_3$)$_2$), −199.5 [1593 Hz] (br s, C_5H_4), −234.8 [2557 Hz] (br s, C_5H_4). ESI-MS (positive ion mode, THF): *m/z* 672.3175 [Ni(η^5-C_5H_4)$_2$(Si*i*Pr_2)$_2$O]$^+$. Anal calcd. for $C_{34}H_{62}NiO_2Si_4$: C 60.60%, H 9.27% Found: C 61.25%, H 9.45%.

iii. Additional Figures and Schemes

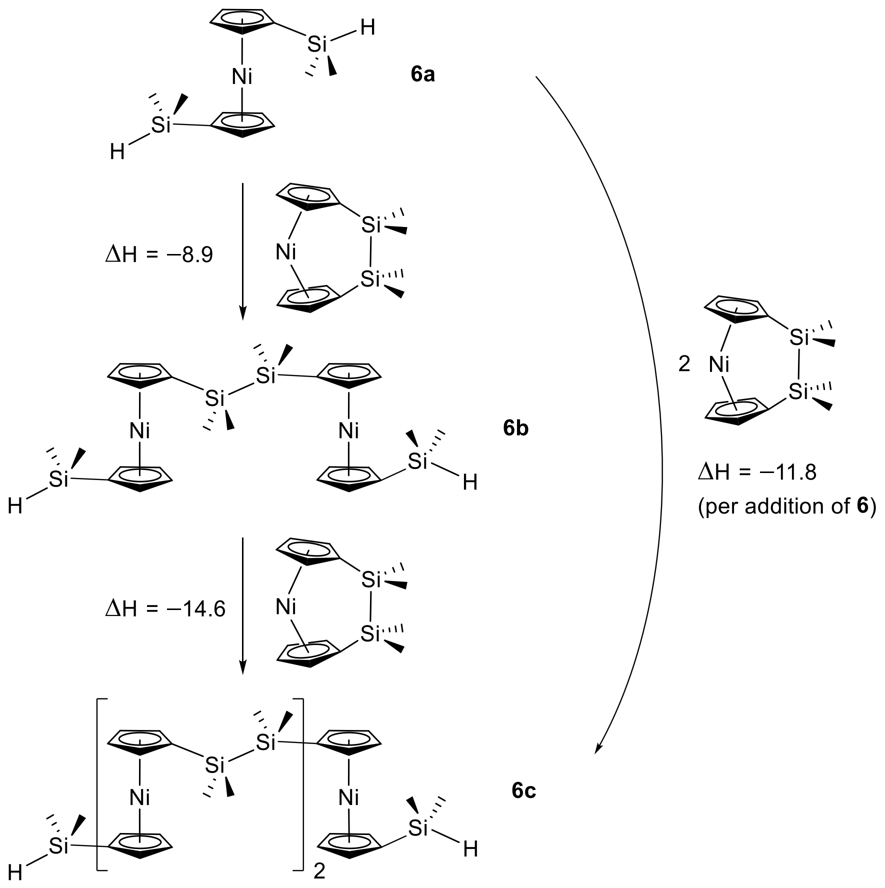

Scheme S1. Scheme describing DFT calculations of the ring-opening of monomer **6** to form linear oligomeric species and values of enthalpic ring-opening (kJ mol^{-1}).

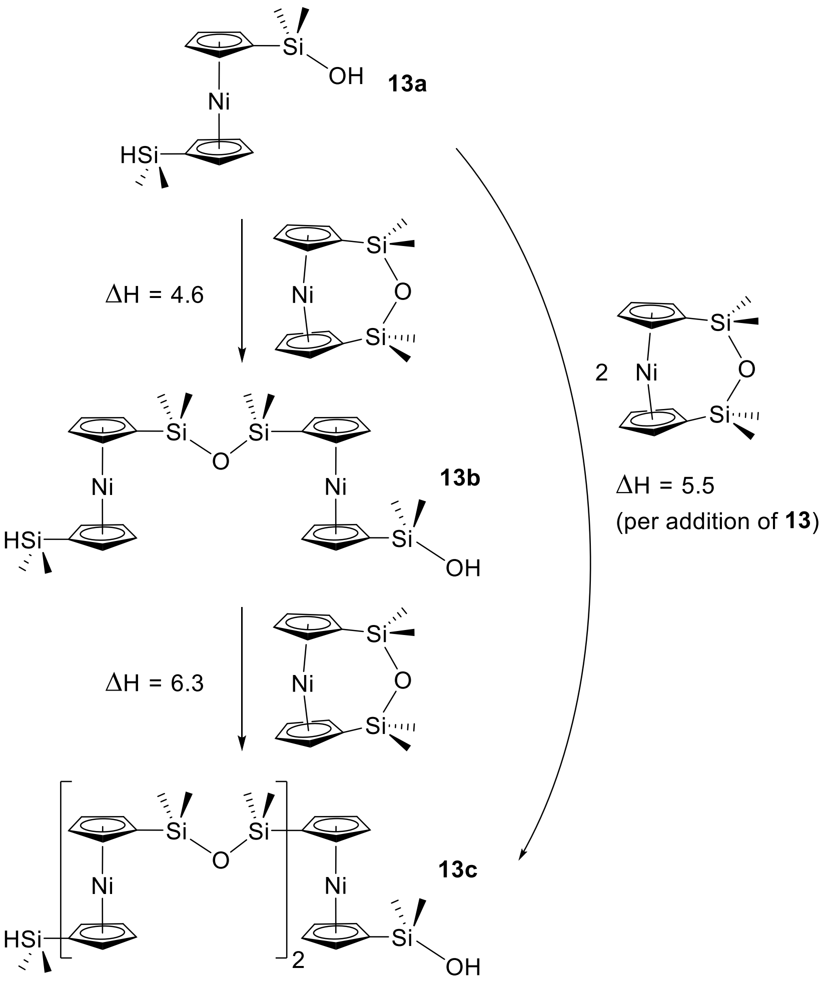

Scheme S2. Scheme describing DFT calculations of the ring-opening of monomer **13** to form linear oligomeric species and values of enthalpic ring-opening (kJ mol^{-1}).

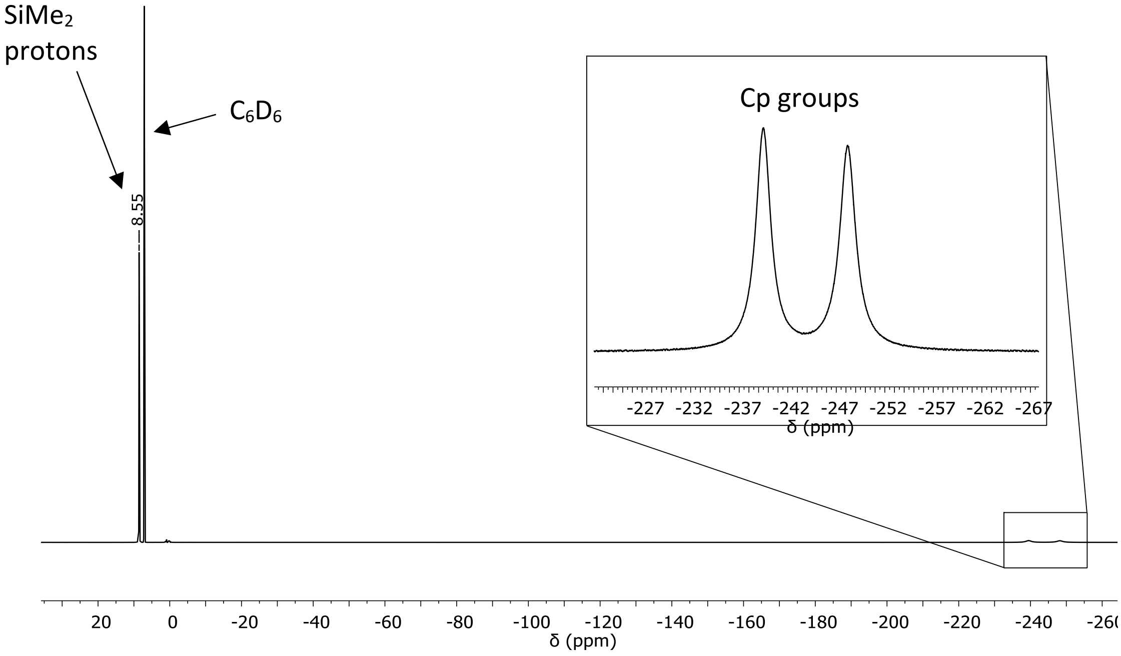

Figure S1. ^{1}H NMR spectrum (500 MHz, C_6D_6) of 1,1,3,3-tetramethyldisila-2-oxa[3]nickelocenophane **13**.

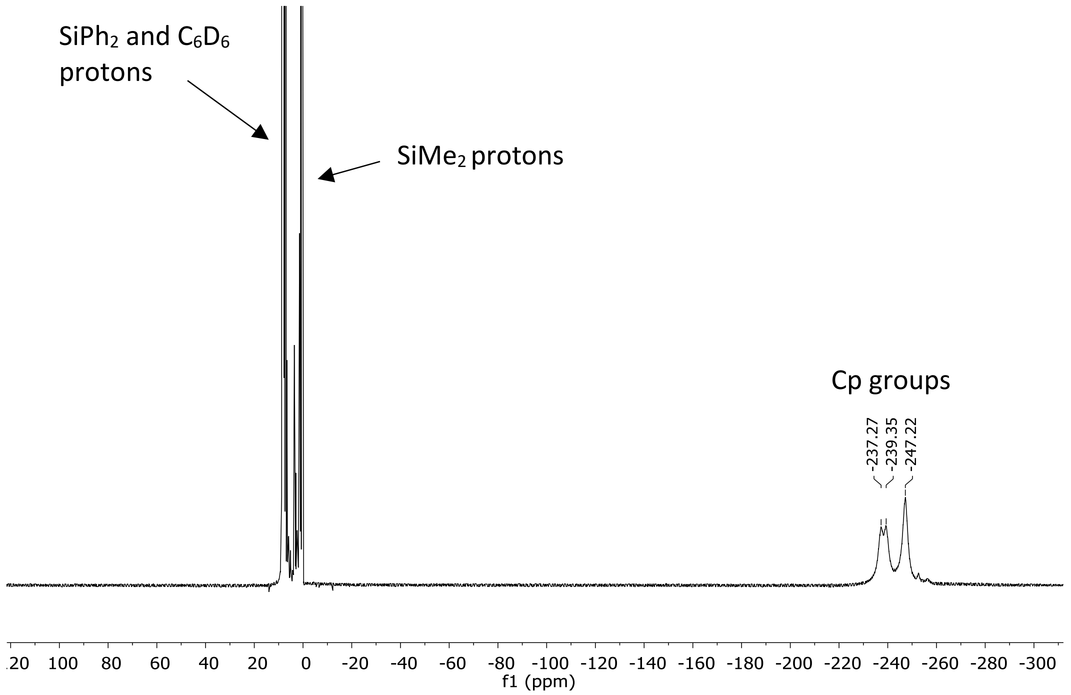

Figure S2. ^{1}H NMR spectrum (500 MHz, C_6D_6) of 1,3-dimethyl-1,3-diphenyldisila-2-oxane[3]nickelocenophane, **14**.

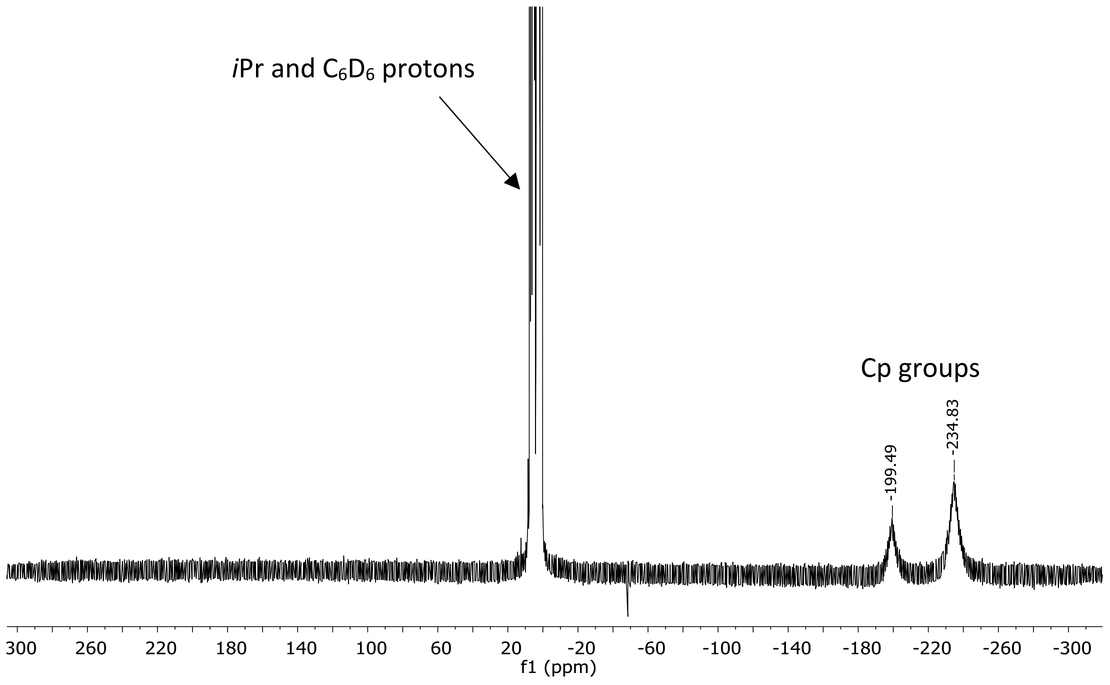

Figure S3. ^{1}H NMR spectrum (500 MHz, C_6D_6) of 1,1′,2,2′-bis(tetraisopropyldisiloxa)nickelocene, **17**.

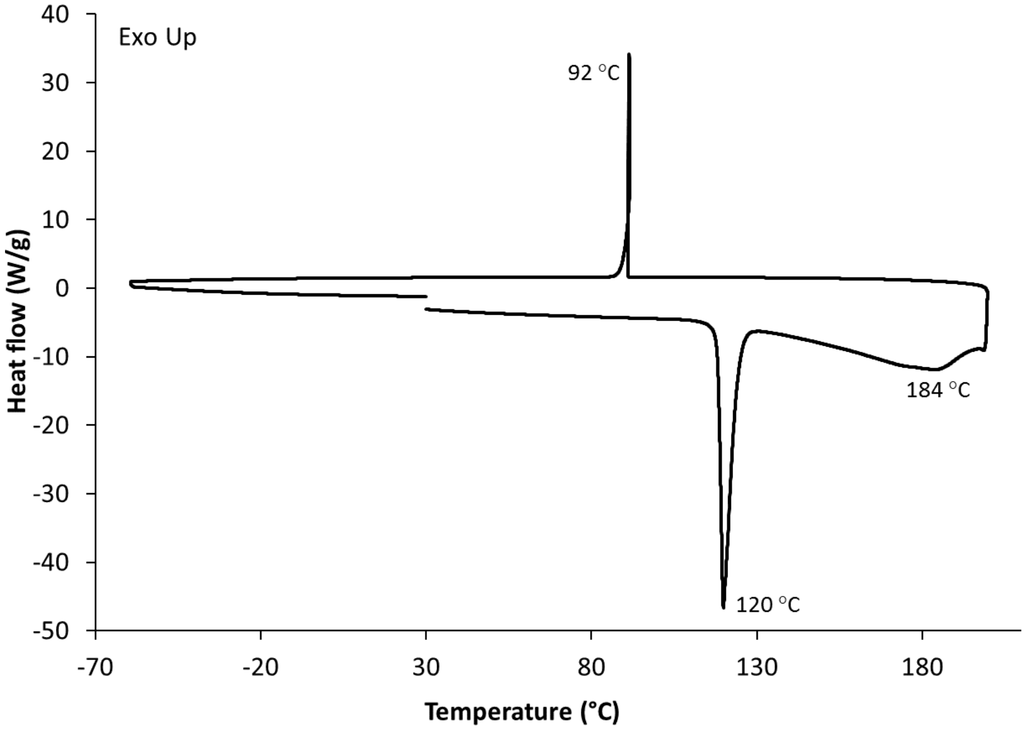

Figure S4. DSC thermogram of **13** obtained at a scan rate of 10 K min^{-1}.

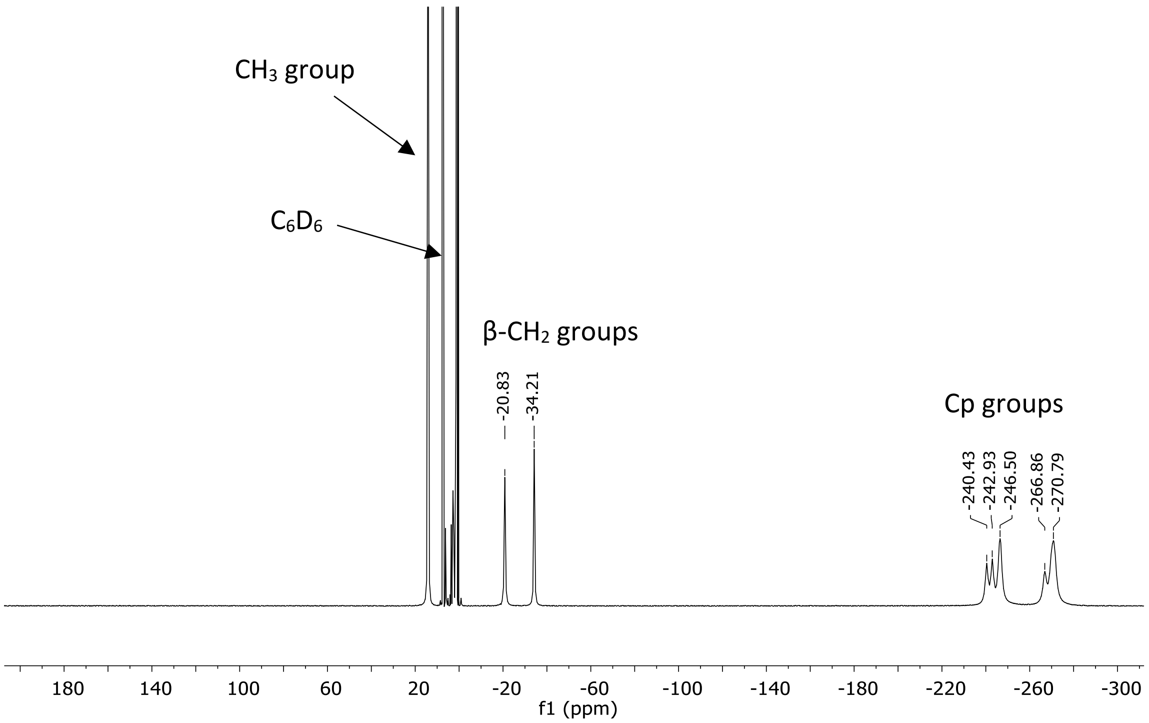

Figure S5. ^{1}H NMR spectrum (500 MHz, C_6D_6) of 1-methyltricarba[3]nickelocenophane, **15**.

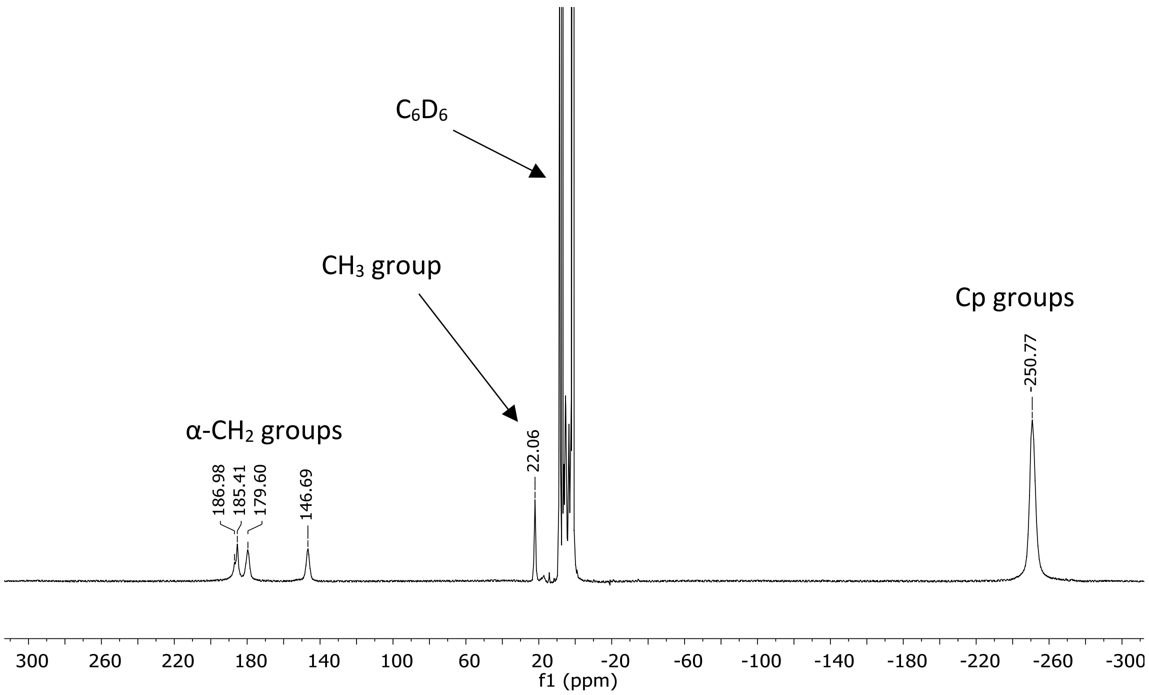

Figure S6. ^{1}H NMR spectrum (500 MHz, C_6D_6) of poly(nickelocenyl-1-methyl-propylene), **18**.

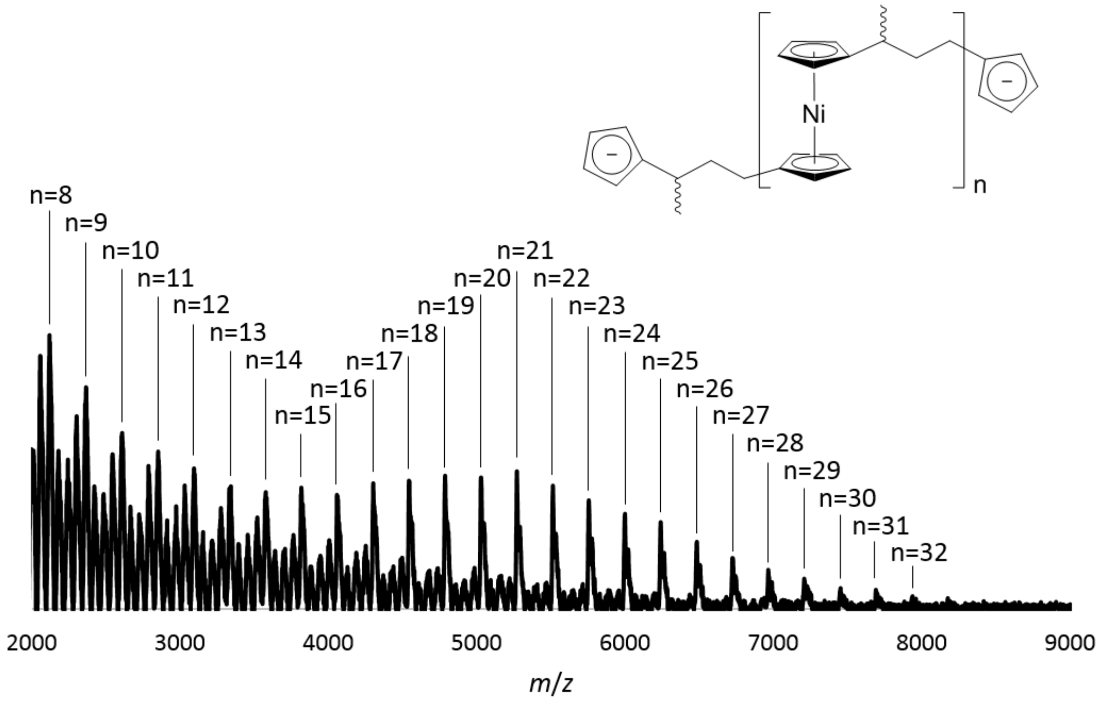

Figure S7. MALDI-TOF spectrum of polymer **18**.

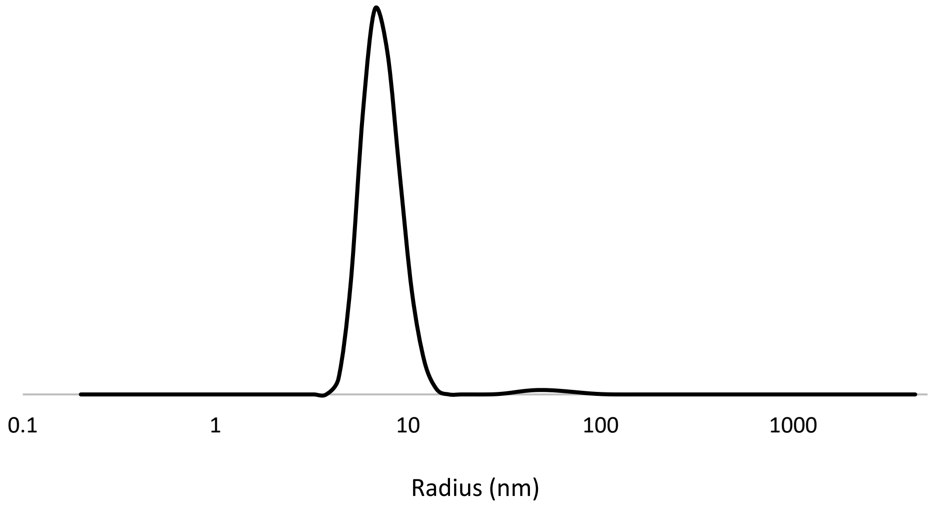

Figure S8. DLS size distribution by volume for **18a** (precipitation of reaction solution into hexanes at 20 °C) (toluene, 20 °C, 1 mg mL^{-1}); hydrodynamic radius (R_h) = 7.42 nm (standard deviation (σ) = 1.69 nm).

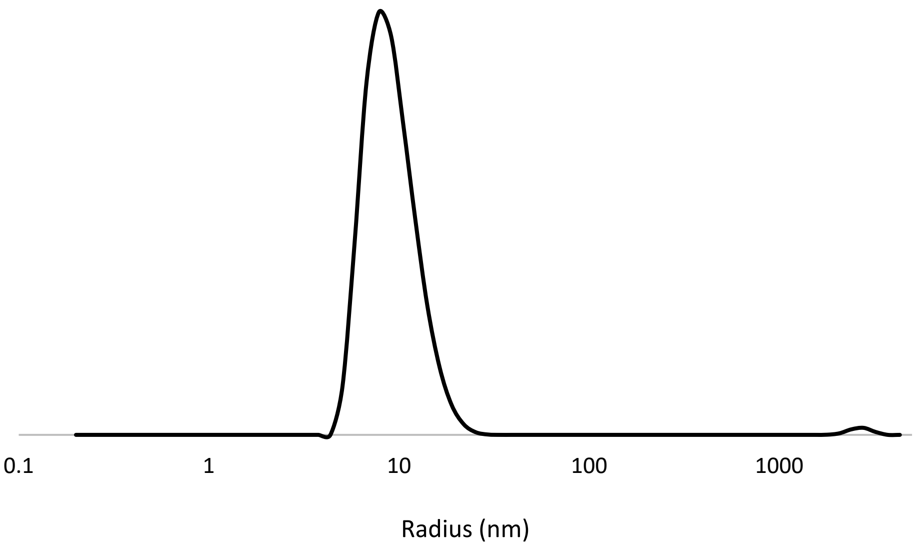

Figure S9. DLS size distribution by volume for **18_a** (precipitation of reaction solution into hexanes at −78 °C) (toluene, 20 °C, 1 mg mL^{-1}); R_h = 9.35 nm (σ = 3.04 nm).

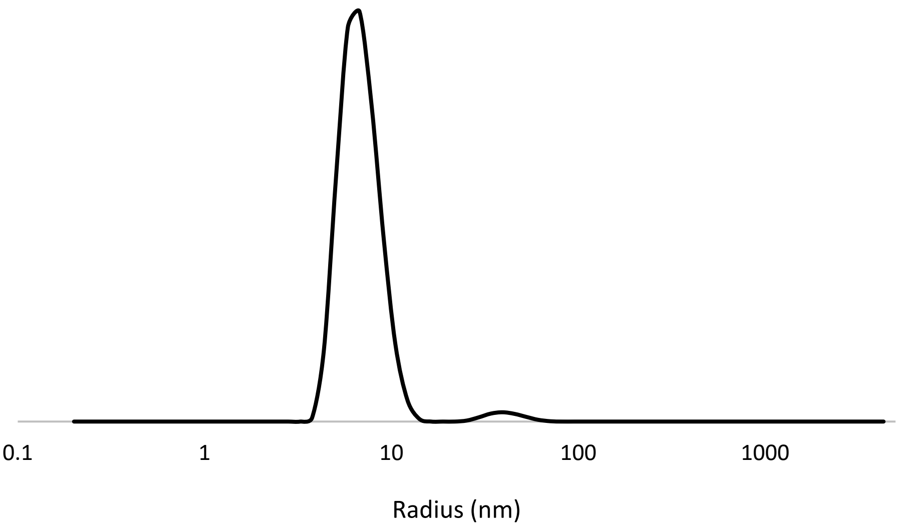

Figure S10. DLS size distribution by volume for **18_b** (precipitation of reaction solution into hexanes at −78 °C) (toluene, 20 °C, 1 mg mL^{-1}); R_h = 6.90 nm (σ = 1.65 nm).

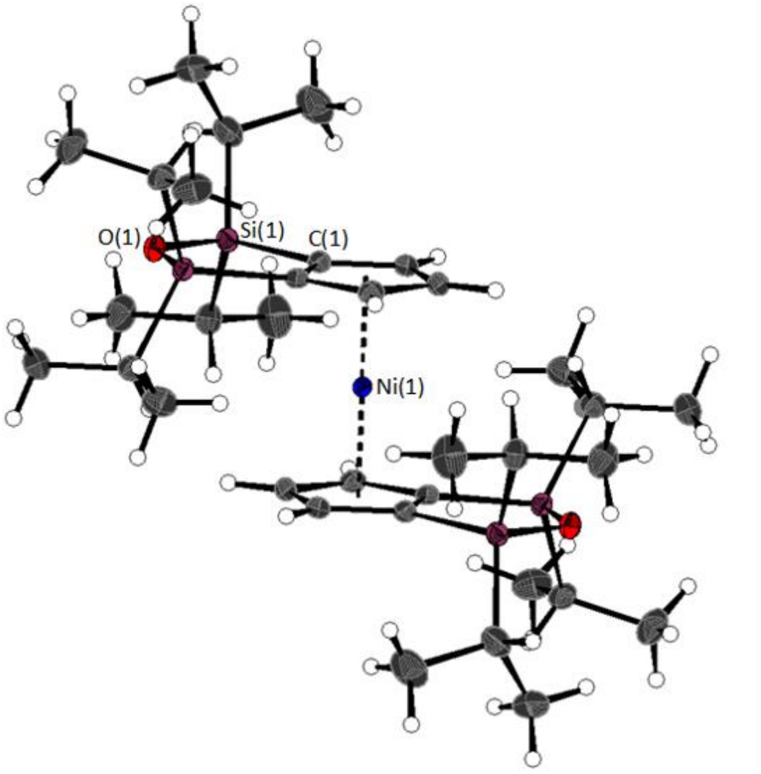

Figure S11. A view of the molecular structure of **17**. Hydrogen atoms are pictured as spheres of arbitrary radii. Thermal ellipsoids displayed at the 50% probability level. Selected distance (Å): Ni(1)–Cp_{cent} = 1.8239(11)/1.8239(11).

iv. Crystallographic Data

Compound	**13**	**14**	**15**	**17**
Empirical formula	$C_{14}H_{20}NiOSi_2$	$C_{24}H_{24}NiOSi_2$	$C_{14}H_{16}Ni$	$C_{34}H_{62}NiO_2Si_4$
Formula weight	319.19	443.32	242.98	673.9
Temperature/K	100.0	100.01	100.01	200(2)
Crystal system	orthorhombic	monoclinic	monoclinic	triclinic
Space group	$P2_12_12_1$	$P2_1/n$	$P2_1/c$	$P\bar{1}$
a/Å	8.3913(2)	9.9885(4)	7.9016(6)	10.1921(17)
b/Å	11.3601(3)	22.3509(9)	9.6856(7)	10.2027(14)
c/Å	15.8756(4)	10.9290(4)	14.8574(11)	10.3527(16)
α/°	90	90	90	83.268(10)
β/°	90	95.756(2)	101.465(5)	83.418(12)
γ/°	90	90	90	62.359(10)
Volume/Å^3	1513.36(7)	2427.62(16)	1114.38(14)	944.9(3)
Z	4	4	4	1
ρ_{calc}g/cm^3	1.401	1.213	1.448	1.184
μ/mm^{-1}	1.426	0.908	1.702	0.667
F(000)	672.0	928.0	512.0	366.0
Crystal size/mm^3	0.20×0.22× 0.17	0.22×0.20× 0.18	0.369×0.332× 0.101	0.527×0.22× 0.21
Radiation	MoKα (λ = 0.71073)	MoKα (λ = 0.71073)	MoKα (λ = 0.71073)	MoKα (λ = 0.71073)
2Θ range for data collection/°	4.408 to 53.448	4.486 to 54.962	5.052 to 56.052	3.97 to 51.356
Index ranges	−10 ≤ h ≤ 10 −14 ≤ k ≤ 14 −20 ≤ l ≤ 20	−9 ≤ h ≤ 12, −28 ≤ k ≤ 28, −14 ≤ l ≤ 14	−10 ≤ h ≤ 10, −9 ≤ k ≤ 12, −19 ≤ l ≤ 19	−12 ≤ h ≤ 12, −12 ≤ k ≤ 12, −12 ≤ l ≤ 12
Reflections collected	12207	22709	13870	13275
Independent reflections	3218 [R_{int} = 0.0335, R_{sigma} = 0.0319]	5546 [R_{int} = 0.0328, R_{sigma} = 0.0285]	2697 [R_{int} = 0.0518, R_{sigma} = 0.0412]	3546 [R_{int} = 0.0706, R_{sigma} = 0.0746]
Data/restraints/ parameters	3218/0/168	5546/0/255	2697/808/274	3546/0/196
Goodness-of-fit on F2	1.292	1.044	1.012	1.073
Final R indexes [I>=2σ (I)]	R_1 = 0.0694 wR_2 = 0.1337	R_1 = 0.0288, wR_2 = 0.0699	R_1 = 0.0269, wR_2 = 0.0522	R_1 = 0.0825, wR_2 = 0.2284
Final R indexes [all data]	R_1 = 0.0916 wR_2 = 0.1400	R_1 = 0.0371, wR_2 = 0.0727	R_1 = 0.0460, wR_2 = 0.0575	R_1 = 0.0983, wR_2 = 0.2546
Largest diff. peak/hole/e Å^{-3}	0.49/−1.10	0.41/−0.25	0.32/−0.32	0.88/−1.55
Flack parameter	N/A	N/A	N/A	N/A

Table S1. Selected distances (Å) and angles (°) in the *ansa* bridges of both **1** and **2**. (α = angle between the plane of each Cp ring, β = [180° − (Cp_{cent}–Cp_{ipso}–C_{bridge})] angle, δ = Cp_{cent}–Ni–Cp'_{cent} angle).[35, 52]

	Distances (Å)		Angles (°)	
Species **2**			α	1.0(3)
			β (C(5), C(10))	0.5(6), 1.6(5)
	Ni(1)–Cp_{cent}	1.813(3), 1.817(3)	δ	178.63(11)
	C(6)–C(7)	1.531(11)	C(5)–C(6)–C(7)	113.9(6)
	C(7)–C(8)	1.515(13)	C(6)–C(7)–C(8)	112.4(7)
	C(8)–C(9)	1.548(11)	C(7)–C(8)–C(9)	115.3(7)
	H(7B)···H(8B)	2.15906(8)	C(8)–C(9)–C(10)	114.6(6)
	C(6)–C(7A)	1.556(12)	H(7B)–C(7)–C(8)–H(8B)	10.2(8)
	C(7A)–C(8A)	1.51(2)	C(5)–C(6)–C(7A)	112.3(8)
	C(8A)–C(9)	1.543(15)	C(6)–C(7A)–C(8A)	116.2(10)
	H(7AA)···H(8AA)	2.1464(1)	C(7A)–C(8A)–C(9)	113.3(11)
			C(8A)–C(9)–C(10)	112.3(7)
			H(7AA)–C(7A)–C(8A)–H(8AA)	5.6(12)
Species **1**			α	16.64(13)
	Ni(1)–Cp_{cent}	1.8039(14), 1.8035(14)	β (both are identical)	4.2(3)
	H(12A)···H(13A)	2.26006(8)	δ	166.33(5)
	H(12A)···H(11B)	2.28442(8)	C(1)–C(11)–C(12)	115.8(3)
	C(11)–C(12)	1.529(5)	C(11)–C(12)–C(13)	115.0(2)
	C(12)–C(13)	1.533(3)	C(12)–C(13)–C(6)	115.28(19)
			H(11B)–C(11)–C(12)–H(12A)	47.6(3)
			H(12A)–C(12)–C(13)–H(13A)	41.3(4)

v. Computational Data

Table S2. Computed total energies for the different species **1**–**1d**, **2**–**2d**, **6**–**6c**, and **13**–**13c**. Total energies/enthalpies in atomic units, entropies in kJ mol^{-1}. E(BS1) is the B3LYP-D2/small basis total electronic energy (Ni: Stuttgart/Dresden ECP and associated basis set; C/H/Si/O: 6-31G(d) basis set). E(BS2) is the B3LYP-D2/large basis total electronic energy (Ni: Stuttgart/Dresden ECP and associated basis set; C/H/Si/O: 6-311G(d) basis set). G(BS1) is the computed free energy at 298.15 K (based on the zero-point energy and correction to 1 M standard state). SCRF is the single-point B3LYP-D2 energy including continuum solvent, and E(BS2)+SCRF+G(BS1) is equal to [E(BS2)+(SCRF–E(BS1))+(G(BS1)–E(BS1))].

	E(BS1)	G(BS1)	H(BS1)	S(BS1)	E(BS2)	SCRF	E(BS2)+SCRF +G(BS1)	H(BS2)
**1**	−674.748612	−674.554760	−674.5033	455.12	−674.857238	−674.752049	−674.666823	−674.6119
**1a**	−675.959080	−675.752951	−675.6923	536.12	−676.068898	−675.962223	−675.865912	−675.8021
**1b**	−1350.716019	−1350.300537	−1350.2019	871.90	−1350.933543	−1350.721821	−1350.523863	−1350.4294
**1c**	−2025.472086	−2024.842267	−2024.7107	1163.24	−2025.796581	−2025.480880	−2025.175555	−2025.0352
**1d**	−2700.227394	−2699.385846	−2699.2183	1480.65	−2700.659065	−2700.238839	−2699.828962	−2699.6500
**2**	−714.060833	−713.840522	−713.7854	486.94	−714.178003	−714.063998	−713.960857	−713.9026
**2a**	−715.271148	−715.038015	−714.9742	564.45	−715.389313	−715.274271	−715.159302	−715.0923
**2b**	−1429.340050	−1428.870193	−1428.7659	921.70	−1429.574782	−1429.346289	−1429.111163	−1429.0007
**2c**	−2143.409219	−2142.702841	−2142.5579	1281.67	−2143.760472	−2143.418492	−2143.063368	−2142.9091
**2d**	−2857.477942	−2856.532450	−2856.3491	1620.78	−2857.945468	−2857.490286	−2857.012320	−2856.8166
**6**	−1295.512986	−1295.264593	−1295.1940	624.23	−1295.683469	−1295.516897	−1295.438988	−1295.3645
**6a**	−1296.713506	−1296.454339	−1296.3768	685.13	−1296.884933	−1296.717266	−1296.629525	−1296.5483
**6b**	−2592.233966	−2591.709468	−2591.5761	1178.55	−2592.573931	−2592.240774	−2592.056241	−2591.9161
**6c**	−3887.755937	−3886.964451	−3886.7772	1655.51	−3888.264898	−3887.765783	−3887.483258	−3887.2861
**13**	−1370.842034	−1370.592044	−1370.5176	658.06	−1371.038278	−1370.846223	−1370.792478	−1370.7138
**13a**	−1372.026752	−1371.764481	−1371.6822	727.08	−1372.224668	−1372.031289	−1371.966933	−1371.8801
**13b**	−2742.871124	−2742.339774	−2742.2002	1234.00	−2743.263179	−2742.879085	−2742.739791	−2742.5922
**13c**	−4113.714798	−4112.913013	−4112.7175	1728.30	−4114.300991	−4113.726166	−4113.510574	−4113.3037

Table S3. Computed relative energies, free energies, enthalpies and entropies for the formation of species **1b**–**1d**, **2b**–**2d**, **6b**–**6c**, and **13b**–**13c**.

	Route	ΔG(BS1)	ΔH(BS1)	ΔS(BS1)	ΔE(BS2)	Δ[E(BS2)+ SCRF+G(BS1)]	ΔH(BS2)
**1b**	**1a** + **1**	18.8353	−16.61	−119.34	−19.4473	23.2931	−14.20
**1c**	**1b** + **1**	34.2103	−14.44	−163.79	−15.2284	39.7242	−10.10
	**1a** + 2(**1**)	26.5228	−15.53	−141.57	−17.3378	31.5087	−12.15
**1d**	**1c** + **1**	29.3557	−11.55	−137.71	−13.7703	35.2243	−7.74
	**1a** + 3(**1**)	27.4671	−14.2	−140.28	−16.1487	32.7472	−10.68
**2b**	**2a** + **2**	21.9072	−16.61	−129.68	−19.6011	23.6213	−15.03
**2c**	**2b** + **2**	20.6732	−17.04	−126.97	−20.1835	22.7186	−15.34
	**2a** + 2(**2**)	21.2902	−16.83	−128.33	−19.8923	23.1699	−15.18
**2d**	**2c** + **2**	28.6521	−15.26	−147.83	−18.3583	31.2559	−12.90
	**2a** + 3(**2**)	23.7441	−16.30	−134.83	−19.3810	25.8653	−14.42
**6b**	**6a** + **6**	24.8477	−14.00	−130.80	−14.5152	32.2189	−8.89
**6c**	**6b** + **6**	25.2311	−18.51	−147.27	−19.6871	31.4303	−14.61
	**6a** + 2(**6**)	25.0394	−16.26	−139.03	−17.1011	31.8246	−11.75
**13b**	**13a** + **13**	43.9798	−0.92	−151.15	−0.6139	51.5124	4.61
**13c**	**13b** + **13**	49.3725	0.73	−163.77	1.2239	56.9605	6.26
	**13a** + 2(**13**)	46.6761	−0.09	−157.46	0.3050	54.2364	5.44

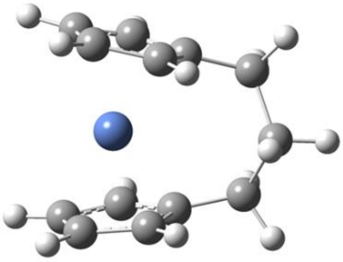

1 E(BS1) = −674.7486 a.u.

	**x**	**y**	**z**
Ni1	−0.000011	−0.719506	−0.019905
C1	−1.994159	−1.530262	−0.623529
C2	−1.789456	−0.028344	−1.110605
C3	−1.995042	−1.402111	−0.794642
C4	−1.994223	−1.530202	−0.623512
C5	−1.788597	−0.233332	−1.175762
C6	−1.645634	−0.706592	−0.108700
C7	−1.789456	−0.028263	−1.110603
C8	−1.995102	−1.402028	−0.794658
C9	−1.645663	−0.706538	−0.108686
C10	−1.788588	−0.233376	−1.175762
C11	−1.317946	−2.174830	−0.249877
C12	−0.000043	−2.650871	−0.411571
C13	−1.318035	−2.174791	−0.249843
H1	−2.132231	−2.447055	−1.182540
H2	−1.723721	−0.385835	−2.109554
H3	−2.133149	−2.204672	−1.508251
H4	−2.132330	−2.446997	−1.182511
H5	−1.718987	−0.005281	−2.230449
H6	−1.723704	−0.385926	−2.109547
H7	−2.133240	−2.204571	−1.508281
H8	−1.718986	−0.005255	−2.230445
H9	−1.290627	−2.430271	−1.317831
H10	−2.135490	−2.770033	−0.181848
H11	−0.000025	−2.363348	−1.471381
H12	−0.000061	−3.749064	−0.395017
H13	−2.135587	−2.769955	−0.181921
H14	−1.290760	−2.430256	−1.317793

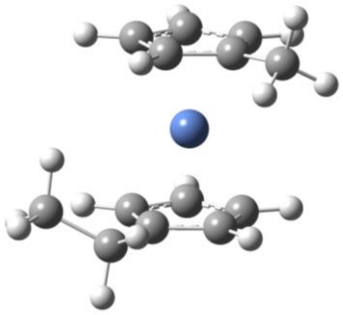

1a E(BS1) = −675.9591 a.u.

	**x**	**y**	**z**
Ni1	−0.238940	−0.370363	−0.180777
C1	−0.731180	−2.066410	−0.869310
C2	−1.201376	−0.850828	−1.445315
C3	−1.916188	−0.116243	−0.449758
C4	−1.866778	−0.888165	−0.750470
C5	−1.141631	−2.087604	−0.492624
C6	−2.113759	−0.668705	−0.519965
C7	−2.469439	−0.496043	−0.223544
C8	−1.986880	−0.352510	−1.556587
C9	−1.322519	−0.902460	−1.646307
C10	−1.397208	−1.527493	−0.367221
C11	−2.657092	−1.178299	−0.678366
C12	−2.732143	−2.110344	−0.537954
C13	−2.486924	−0.966987	−1.945521
H1	−0.149893	−2.828332	−1.373376
H2	−1.054833	−0.534234	−2.471383
H3	−2.303502	−0.609855	−1.701241
H4	−0.927464	−2.868008	−1.212121
H5	−3.014126	−1.347406	−0.167275
H6	−2.091113	−1.077877	−2.353591
H7	−0.833518	−1.304473	−2.524758
H8	−0.988460	−2.496669	−0.107016
H9	−2.192751	−1.710940	−1.518520
H10	−3.682217	−0.945406	−1.005719
H11	−1.731206	−2.400860	−0.875462
H12	−3.288334	−3.022027	−0.292392
H13	−3.242961	−1.629940	−1.380125
H14	−3.462453	−1.469407	−2.003209
H15	−1.753898	−1.625646	−2.424278
H16	−2.557206	−0.051607	−2.543520

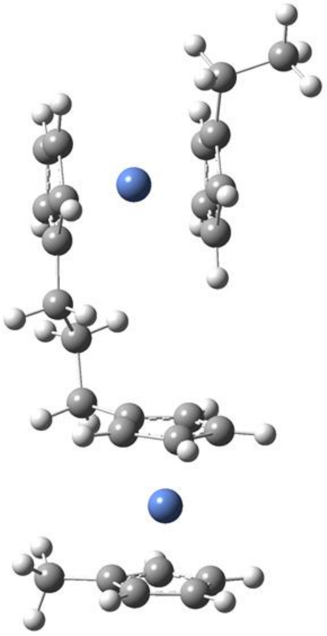

1b E(BS1) = −1350.7160 a.u.

	x	y	z
Ni1	−3.516804	−0.447591	−0.142872
Ni2	−4.010067	−0.655973	−0.175225
C1	−4.291803	−2.536815	−0.190527
C2	−2.920866	−2.567291	−0.577713
C3	−2.126269	−2.098791	−0.512802
C4	−3.027571	−1.772556	−1.572223
C5	−4.358625	−2.046346	−1.143628
C6	−4.742489	−1.444215	−0.173727
C7	−3.442977	−1.731869	−0.343420
C8	−2.475518	−1.446365	−0.662812
C9	−3.171521	−0.974662	−1.812247
C10	−4.563926	−0.971676	−1.509600
C11	−0.622055	−2.010716	−0.588580
C12	−0.085528	−1.805641	−0.758921
C13	−1.610229	−1.588147	−0.627337
C14	−1.971728	−0.306104	−0.079841
C15	−2.369741	−0.162886	−1.443682
C16	−2.531984	−1.223958	−1.731249
C17	−2.236372	−1.951128	−0.544409
C18	−1.897037	−1.011217	−0.471160
C19	−6.058234	1.497572	−0.397659
C20	−6.095870	0.122867	−0.769749
C21	−5.747690	−0.668490	−0.366227
C22	−5.484077	0.235571	−1.439387
C23	−5.678374	1.567973	−0.971815
C24	6.056198	1.680203	−0.525105
C25	−6.601289	−3.103480	−0.302197
C26	−5.737063	−2.170229	−0.439168
H1	−5.133453	−2.822619	−0.808971
H2	−2.547667	−2.886132	−1.542747
H3	−2.738282	−1.383232	−2.541295
H4	−5.259796	−1.894747	−1.724441
H5	−3.231517	−2.100633	−1.340286
H6	−1.402324	−1.552167	−0.561630
H7	−2.723246	−0.658179	−2.745742
H8	−5.359060	−0.660541	−2.176955
H9	−0.228934	−2.930569	−1.051262
H10	−0.347079	−1.194571	−1.268182
H11	−0.087755	−2.678456	1.402495
H12	−0.356048	−0.943222	1.273603
H13	−2.046927	−1.593785	1.634700
H14	−2.053447	−2.439653	0.093484
H15	−2.523152	−0.978870	−2.140070
H16	−2.838000	−1.646518	−2.679955
H17	−2.281910	−3.026613	−0.427103
H18	−1.635138	−1.250919	1.495123
H19	−6.261935	−2.339206	−1.047737
H20	−6.346170	−0.264055	−1.750481
H21	−5.189123	−0.049694	2.442299
H22	−5.543199	−2.472515	1.551258
H23	−6.798485	−0.952241	−0.172687
H24	−5.938566	−1.505818	−1.602576
H25	−6.762457	−3.298342	0.764109
H26	−7.556243	−3.244535	−0.822432
H27	−5.896407	−3.855286	−0.674797
H28	−5.497818	−2.618408	−0.531505
H29	−6.718654	−2.558753	0.743911
H30	−5.003543	−2.533901	1.167101

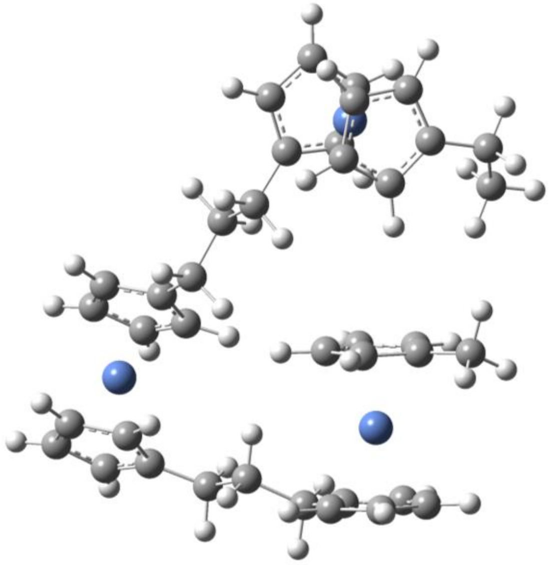

1c E(BS1) = −2025.4721 a.u.

	x	y	z
Ni1	−0.341218	−3.656328	0.019486
Ni2	−4.564241	−2.278690	0.061257
Ni3	−4.639858	−1.834329	0.248645
C1	1.258045	5.469420	0.902418
C2	2.246996	4.443222	0.906033
C3	2.525002	4.070768	−0.444734
C4	1.684142	4.870916	−1.276854
C5	0.909162	5.736295	−0.451485
C6	−1.392128	2.690273	−1.019240
C7	−0.451524	1.691266	−0.631804
C8	−0.366591	1.702009	0.790079
C9	−1.253828	2.707945	1.271895
C10	−1.898709	3.322085	0.156274
C11	3.582678	3.113960	−0.936609
C12	3.844666	1.890930	−0.043474
C13	4.999925	1.028135	−0.570885
C14	5.357620	−0.162973	0.289058
C15	6.437728	−1.064687	0.040722
C16	6.497492	−2.012822	1.102253
C17	5.444550	−1.713937	2.011384
C18	4.742994	−0.578379	1.508615
C19	2.453360	−2.937163	−0.319138
C20	3.212332	−4.063301	0.119936
C21	4.253631	−4.308308	−0.820632
C22	4.148542	−3.332980	−1.851359
C23	3.044604	−2.486955	−1.540678
C24	−2.954975	4.391133	0.213462
C25	1.220464	−2.375722	0.336983
C26	−0.094216	−2.926885	−0.252113
C27	−1.334078	−2.297117	0.413887
C28	−2.638115	−2.763247	−0.177142
C29	−3.221825	−2.309841	−1.399981
C30	−4.433754	−3.026219	−1.620076
C31	−4.611306	−3.926844	−0.531930
C32	−3.507041	−3.763761	0.352697
C33	−5.859870	0.076327	0.183185
C34	−4.678725	0.284879	0.960480
C35	−4.712802	−0.594907	2.081158
C36	−5.910147	−1.361043	2.002909
C37	−6.610590	−0.949778	0.832709
C38	−6.304628	0.856428	−1.029347
C39	−5.211622	1.128193	−2.072692
H1	0.835755	5.947605	1.777271
H2	2.710516	4.021812	1.789061
H3	1.653165	4.828569	−2.359247
H4	0.177536	6.457671	−0.793045
H5	−1.673621	2.938208	−2.035750
H6	0.105484	1.047515	−1.300779
H7	0.266661	1.068302	1.397581
H8	−1.413907	2.969186	2.311189
H9	4.528171	3.668579	−1.049103
H10	3.317817	2.775712	−1.947793
H11	4.083238	2.223653	0.974908
H12	2.928270	1.290920	0.030424
H13	5.892036	1.664874	−0.670413
H14	4.763014	0.695728	−1.592077
H15	7.103913	−1.025625	−0.813087
H16	7.210601	−2.822454	1.193993
H17	5.212427	−2.253394	2.920986
H18	3.887083	−0.110324	1.977440
H19	3.027375	−4.629304	1.025327
H20	5.002965	−5.086995	−0.753549
H21	4.802324	−3.238507	−2.709353
H22	2.702425	−1.645698	−2.132025
H23	−2.943886	5.015236	−0.686839
H24	−3.958991	3.952907	0.295441
H25	−2.816486	5.047009	1.079918
H26	1.214448	−1.281083	0.239394
H27	1.246306	−2.587895	1.414589
H28	−0.119269	−2.729735	−1.331776
H29	−0.122631	−4.018189	−0.137121
H30	−1.318042	−2.524810	1.488604
H31	−1.259332	−1.204422	0.329934
H32	−2.808320	−1.544736	−2.045590
H33	−5.105449	−2.900342	−2.460093

H34	−5.444484	−4.604538	−0.394074
H35	−3.353690	−4.297865	1.283135
H36	−3.885259	0.988439	0.738969
H37	−3.950478	−0.678098	2.845748
H38	−6.223526	−2.130565	2.697231
H39	−7.560173	−1.342778	0.488600
H40	−7.138184	0.325135	−1.506610
H41	−6.719210	1.820342	−0.695635
H42	−4.828266	0.191143	−2.490241
H43	−5.606489	1.733817	−2.896874
H44	−4.363434	1.668466	−1.637280

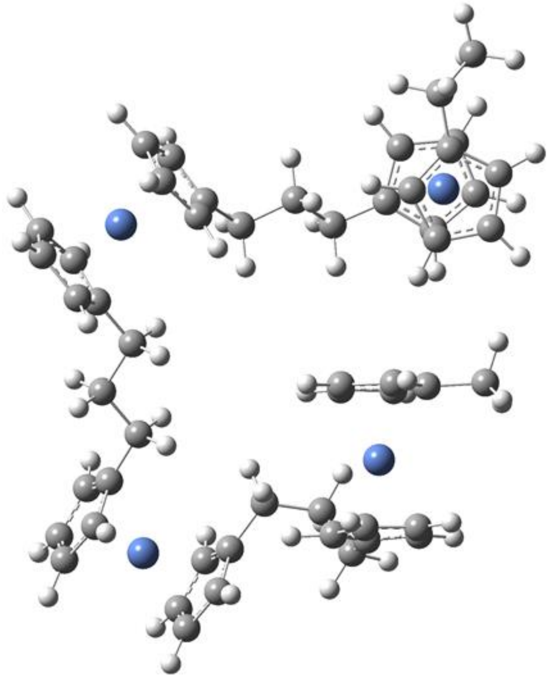

1d E(BS1) = −2700.2274 a.u.

	x	y	z
Ni1	0.872447	−3.569457	−1.124498
Ni2	6.677169	0.094423	0.570929
Ni3	−1.009033	5.249568	−0.563514
Ni4	−5.746786	−1.522541	0.663172
C1	2.087858	−3.924225	−2.931886
C2	2.978482	−3.480693	−1.912038
C3	2.974309	−4.435787	−0.847352
C4	2.055239	−5.462387	−1.224436
C5	1.513889	−5.155376	−2.505974
C6	−0.406903	−3.185448	0.660701
C7	0.141335	−1.948418	0.207604
C8	−0.355867	−1.707635	−1.104010
C9	−1.207391	−2.795096	−1.454138
C10	−1.249950	−3.713915	−0.362865
C11	3.842266	−4.469858	0.390487
C12	3.599479	−3.388158	1.464744
C13	4.056131	−1.957374	1.096768
C14	5.550982	−1.814026	0.965004
C15	6.464455	−1.573855	2.037790
C16	7.791427	−1.583655	1.521237
C17	7.712030	−1.824330	0.120844
C18	6.335735	−1.963868	−0.220528
C19	5.352931	1.878012	0.218409
C20	6.249238	1.730293	−0.884280
C21	7.584469	1.873957	−0.410032
C22	7.525847	2.107744	0.992931
C23	6.154630	2.107864	1.378400
C24	−2.079744	−4.965123	−0.280090
C25	3.848392	1.891681	0.152732
C26	3.270642	3.303854	−0.073158
C27	1.729978	3.311228	−0.149826
C28	1.163302	4.690929	−0.357131
C29	0.816005	5.627784	0.663668
C30	0.412452	6.846745	0.046452
C31	0.503880	6.672348	−1.363291
C32	0.962158	5.346668	−1.610163
C33	−2.470244	3.654129	0.038491
C34	−2.480090	3.788467	−1.383852
C35	−2.910319	5.105632	−1.715297
C36	−3.168251	5.798634	−0.499271
C37	−2.895249	4.907043	0.577489
C38	−2.164508	2.402409	0.818024
C39	−3.411626	1.527645	1.061859
C40	−3.086143	0.252406	1.854057
C41	−4.260847	−0.648544	2.143432
C42	−4.211915	−2.074057	2.196138
C43	−5.485765	−2.563050	2.604663
C44	−6.338757	−1.440149	2.798660
C45	−5.586856	−0.263539	2.510126
C46	−7.228435	−0.897066	−0.939460
C47	−5.899922	−0.564243	−1.344733
C48	−5.136966	−1.762792	−1.451307
C49	−5.988751	−2.848379	−1.102835
C50	−7.271799	−2.315755	−0.785357
C51	−8.373342	0.077131	−0.811804
C52	−9.399128	−0.257236	0.279308
H1	1.878465	−3.409626	−3.861354
H2	3.570673	−2.575666	−1.952603
H3	1.828483	−6.341224	−0.632077
H4	0.792513	−5.748097	−3.054060
H5	−0.219813	−3.650766	1.621202

H6	0.823687	−1.313567	0.757563
H7	−0.113763	−0.860647	−1.733666
H8	−1.724056	−2.919389	−2.398606
H9	4.898180	−4.430513	0.089072
H10	3.702911	−5.449228	0.865041
H11	2.530923	−3.364825	1.712936
H12	4.129792	−3.691360	2.377355
H13	3.561425	−1.641677	0.171078
H14	3.698986	−1.276916	1.881194
H15	6.186673	−1.407389	3.071998
H16	8.697811	−1.416190	2.089422
H17	8.547026	−1.876364	−0.566441
H18	5.946877	−2.151151	−1.214105
H19	5.956308	1.539164	−1.909964
H20	8.484524	1.799994	−1.007383
H21	8.373230	2.243718	1.653113
H22	5.776845	2.256700	2.383194
H23	−1.626904	−5.703677	0.390450
H24	−3.085208	−4.745619	0.104585
H25	−2.202520	−5.433689	−1.262827
H26	3.430081	1.481158	1.082213
H27	3.506050	1.232545	−0.657202
H28	3.598108	3.966302	0.738486
H29	3.683803	3.724190	−0.999174
H30	1.324389	2.876822	0.774290
H31	1.410905	2.650297	−0.967811
H32	0.854158	5.437181	1.729836
H33	0.079479	7.740694	0.558599
H34	0.251024	7.409396	−2.115120
H35	1.130238	4.903707	−2.584806
H36	−2.202349	3.014135	−2.089339
H37	−3.006914	5.512062	−2.714311
H38	−3.493807	6.827312	−0.407940
H39	−2.989010	5.136855	1.632402
H40	−1.411991	1.805510	0.285070
H41	−1.720025	2.667007	1.787393
H42	−3.858475	1.255806	0.097912
H43	−4.164295	2.122539	1.595525
H44	−2.321032	−0.322678	1.315768
H45	−2.621526	0.545822	2.809707
H46	−3.339982	−2.672481	1.959539
H47	−5.762396	−3.602749	2.727741
H48	−7.377762	−1.473321	3.101382
H49	−5.959188	0.751708	2.569337
H50	−5.542652	0.438513	−1.546810
H51	−4.090170	−1.832199	−1.718406
H52	−5.708800	−3.893792	−1.065133
H53	−8.133006	−2.894428	−0.475367
H54	−8.896640	0.135831	−1.778840
H55	−7.966848	1.081204	−0.634305
H56	−9.862086	−1.235906	0.109093
H57	−10.201812	0.488656	0.296457
H58	−8.929508	−0.276898	1.268805

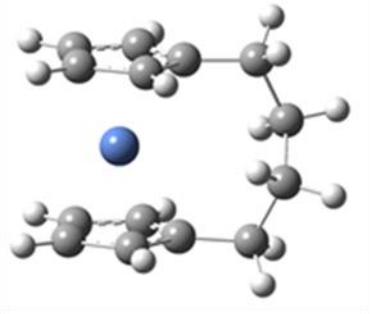

2 E(BS1) = −714.0608 a.u.

	**x**	**y**	**z**
Ni1	−0.026880	0.781974	0.000955
C1	1.882136	−0.422612	−0.004418
C2	1.895621	0.519507	−1.080079
C3	1.842632	1.835360	−0.536874
C4	1.787037	1.719429	0.881125
C5	1.810919	0.332082	1.204966
C6	−1.964774	1.705248	0.535653
C7	−1.899312	1.592976	−0.882233
C8	−1.827385	0.207121	−1.205496
C9	−1.848907	−0.550320	0.004135
C10	−1.928622	0.388954	1.079411
C11	−1.818589	−2.053876	0.140818
C12	−0.451185	−2.645732	0.554486
C13	0.631184	−2.608711	−0.554554
C14	1.954797	−1.924695	−0.140804
H1	1.947869	0.270206	−2.133467
H2	1.840830	2.758565	−1.102685
H3	1.734420	2.538458	1.587311
H4	1.781577	−0.086727	2.203938
H5	−2.027682	2.626586	1.101016
H6	−1.900542	2.413454	−1.588712
H7	−1.768236	−0.209191	−2.204231
H8	−1.964589	0.137060	2.132828
H9	−2.134875	−2.510630	−0.806273
H10	−2.566641	−2.352289	0.887749
H11	−0.098288	−2.106370	1.442818
H12	−0.607590	−3.684105	0.873835
H13	0.242179	−2.094473	−1.442715

H14	0.858045	−3.633969	−0.873993
H15	2.721623	−2.171643	−0.887467
H16	2.301240	−2.358574	0.806489

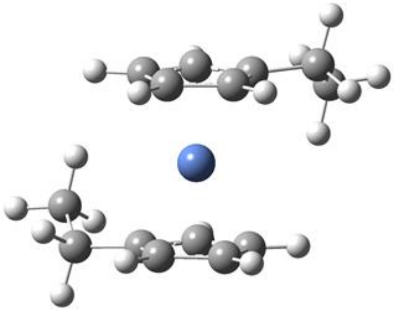

2a E(BS1) = −715.2712 a.u.

	**x**	**y**	**z**
Ni1	−0.000047	0.000096	0.423210
C1	−0.912214	−1.742386	1.455192
C2	−1.891809	−0.712267	1.371279
C3	−2.174414	−0.455730	−0.005250
C4	−1.345659	−1.332536	−0.769004
C5	−0.571954	−2.126112	0.127771
C6	1.345766	1.332442	−0.769099
C7	0.572132	2.126181	0.127580
C8	0.912311	1.742533	1.455037
C9	1.891778	0.712296	1.371238
C10	2.174391	0.455620	−0.005251
C11	−3.224451	0.496866	−0.521879
C12	−2.923296	1.119473	−1.891341
C13	3.224317	−0.497161	−0.521762
C14	2.923303	−1.119550	−1.891352
H1	−0.488314	−2.147128	2.365647
H2	−2.353647	−0.204116	2.209768
H3	−1.309690	−1.389353	−1.849595
H4	0.152680	−2.880067	−0.152748
H5	1.309833	1.389165	−1.849695
H6	−0.152384	2.880217	−0.153025
H7	0.488440	2.147415	2.365442
H8	2.353517	0.204156	2.209786
H9	−3.375781	1.294836	0.216729
H10	−4.185997	−0.036026	−0.582983
H11	−1.989288	1.691554	−1.868813
H12	−3.730976	1.795307	−2.194041
H13	−2.827269	0.352514	−2.668256
H14	4.185998	0.035518	−0.582606
H15	3.375318	−1.295245	0.216789
H16	2.827680	−0.352489	−2.668217
H17	3.730859	−1.795587	−2.193927
H18	1.989125	−1.691365	−1.869099

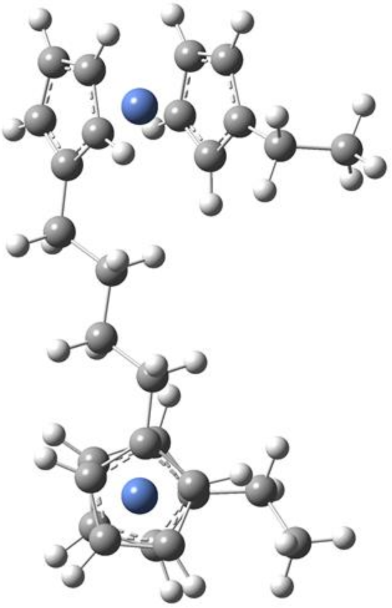

2b E(BS1) = −1429.3401 a.u.

	**x**	**y**	**z**
Ni1	4.232197	−0.406122	−0.133971
Ni2	−4.420792	−0.127734	−0.405009
C1	−5.475858	−2.033935	−0.836504
C2	−4.148021	−2.133558	−1.342267
C3	−3.231140	−2.043995	−0.250778
C4	−4.011745	−1.872083	0.933422
C5	−5.391269	−1.869983	0.574030
C6	−4.041004	1.886999	0.526597
C7	−3.202885	1.729799	−0.619849
C8	−4.028275	1.583486	−1.772249
C9	−5.384717	1.644733	−1.346857
C10	−5.391159	1.828082	0.065955
C11	−1.734311	−2.204989	−0.351551
C12	−0.918809	−1.474298	0.725717
C13	0.594227	−1.663516	0.558718
C14	1.406657	−0.972559	1.664530
C15	2.904398	−1.117957	1.554520
C16	3.635216	−2.228471	1.033689
C17	5.027464	−1.985628	1.216822
C18	5.170985	−0.719836	1.849715
C19	3.865737	−0.184221	2.050154
C20	5.064977	−0.470626	−2.196114
C21	5.501176	0.740440	−1.584718
C22	4.356630	1.538600	−1.281670
C23	3.210680	0.797878	−1.704974
C24	3.644861	−0.434874	−2.273303

C25	4.336498	2.936718	−0.714707
C26	5.443428	3.244350	0.302521
C27	−3.588685	2.163512	1.937140
C28	−3.443768	3.668673	2.229842
H1	−6.385238	−2.057886	−1.423706
H2	−3.871999	−2.262515	−2.382233
H3	−3.623822	−1.766278	1.938689
H4	−6.224840	−1.747819	1.254277
H5	−2.119315	1.728347	−0.610139
H6	−3.684300	1.436624	−2.788423
H7	−6.257064	1.547606	−1.980928
H8	−6.271807	1.905020	0.692731
H9	−1.403730	−1.870942	−1.344716
H10	−1.489221	−3.278158	−0.304194
H11	−1.163350	−0.403525	0.699559
H12	−1.218381	−1.834603	1.719776
H13	0.910681	−1.275996	−0.419341
H14	0.823146	−2.738347	0.552999
H15	1.151209	0.095962	1.687203
H16	1.081351	−1.374867	2.637294
H17	3.206213	−3.108327	0.571205
H18	5.832769	−2.641518	0.910843
H19	6.104904	−0.240085	2.113838
H20	3.632149	0.767621	2.512655
H21	5.702377	−1.281240	−2.526429
H22	6.530906	1.005853	−1.380569
H23	2.183472	1.131952	−1.617247
H24	3.007321	−1.211421	−2.676970
H25	3.356967	3.119264	−0.253847
H26	4.412678	3.656376	−1.544403
H27	5.381230	2.575394	1.167892
H28	5.362220	4.276604	0.661249
H29	6.439023	3.127173	−0.140254
H30	−4.302936	1.727319	2.647574
H31	−2.627001	1.667689	2.122254
H32	−4.397897	4.188557	2.087947
H33	−3.113886	3.837931	3.261782
H34	−2.710874	4.129750	1.558086

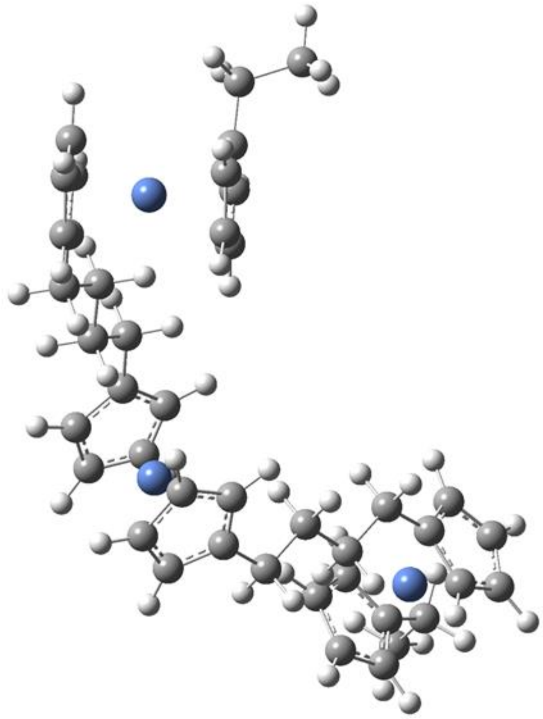

2c E(BS1) = −2143.4092 a.u.

	**x**	**y**	**z**
Ni1	−0.002877	−2.508744	0.346278
Ni2	−8.010691	0.822152	0.051379
Ni3	7.782878	1.243394	0.008802
C1	7.977716	3.016236	1.331489
C2	6.811907	2.301760	1.732470
C3	5.881481	2.299635	0.648458
C4	6.495538	3.010910	−0.426746
C5	7.781949	3.457443	−0.007364
C6	9.539344	0.336704	−1.076827
C7	8.335673	0.012728	−1.772875
C8	7.504387	−0.761308	−0.912394
C9	8.187573	−0.918752	0.326943
C10	9.436527	−0.241263	0.225055
C11	10.72732	1.073734	−1.638160
C12	11.73371	0.138213	−2.333840
C13	4.476187	1.751134	0.644084
C14	4.271508	0.447022	1.431094
C15	2.821426	−0.051298	1.389167
C16	2.612692	−1.350738	2.182331
C17	1.207934	−1.900498	2.163028
C18	0.856473	−3.282170	2.252986
C19	−0.562699	−3.393614	2.299969
C20	−1.102546	−2.078545	2.229957
C21	−0.014410	−1.162320	2.142375
C22	1.126288	−2.483201	−1.558785
C23	0.123671	−1.472241	−1.617976

C24	−1.161045	−2.093886	−1.555840
C25	−0.936188	−3.499616	−1.442600
C26	0.467991	−3.739876	−1.447068
C27	−2.483673	−1.379631	−1.689352
C28	−3.678050	−2.069126	−1.013087
C29	−4.988215	−1.288461	−1.177961
C30	−6.186121	−1.989551	−0.518566
C31	−7.507831	−1.272321	−0.641480
C32	−7.984486	−0.507029	−1.749283
C33	−9.324220	−0.100929	−1.480573
C34	−9.686785	−0.608501	−0.201451
C35	−8.567631	−1.321949	0.315027
C36	−7.609205	1.916297	1.948259
C37	−8.561326	2.660720	1.194770
C38	−7.955249	3.071692	−0.031505
C39	−6.620039	2.565726	−0.026021
C40	−6.405011	1.856700	1.191458
C41	−8.574499	3.949846	−1.087742
C42	−8.366087	5.450527	−0.810381
H1	8.861772	3.179694	1.935096
H2	6.660550	1.839101	2.699654
H3	6.047153	3.185215	−1.397775
H4	8.487852	4.021102	−0.604316
H5	8.096936	0.311435	−2.786865
H6	6.523916	−1.150461	−1.156037
H7	7.818048	−1.445595	1.197694
H8	10.18474	−0.167475	1.005390
H9	11.23858	1.616994	−0.832875
H10	10.38632	1.831815	−2.355231
H11	12.12157	−0.610350	−1.633718
H12	12.58396	0.703815	−2.733133
H13	11.26059	−0.397173	−3.164719
H14	3.795684	2.514780	1.053576
H15	4.157541	1.598600	−0.396162
H16	4.570716	0.597754	2.477748
H17	4.940704	−0.325533	1.028671
H18	2.160311	0.731748	1.786110
H19	2.514730	−0.212348	0.346625
H20	2.910781	−1.172850	3.227938
H21	3.301866	−2.119028	1.805552
H22	1.560172	−4.105728	2.283866
H23	−1.128638	−4.314575	2.362617
H24	−2.153349	−1.818028	2.233801
H25	−0.103816	−0.085666	2.071000
H26	2.196822	−2.322885	−1.582664
H27	0.299166	−0.406493	−1.706150
H28	−1.702887	−4.259514	−1.360347
H29	0.947400	−4.707110	−1.363012
H30	−2.382028	−0.360160	−1.292265
H31	−2.713311	−1.258043	−2.760086
H32	−3.460626	−2.204517	0.055168
H33	−3.805011	−3.077424	−1.431364
H34	−4.876588	−0.283327	−0.748989
H35	−5.189858	−1.145488	−2.248750
H36	−5.967694	−2.153864	0.545541
H37	−6.285638	−2.994731	−0.958616
H38	−7.424603	−0.271262	−2.645446
H39	−9.949424	0.497695	−2.131216
H40	−10.636404	−0.463187	0.298043
H41	−8.521071	−1.825039	1.273705
H42	−7.777391	1.461860	2.916587
H43	−9.580368	2.875315	1.494617
H44	−5.894986	2.702174	−0.819800
H45	−5.491716	1.354267	1.484380
H46	−8.147970	3.703132	−2.068682
H47	−9.650490	3.743730	−1.157463
H48	−7.299025	5.696616	−0.768531
H49	−8.826252	6.062415	−1.595341
H50	−8.811291	5.736915	0.149093

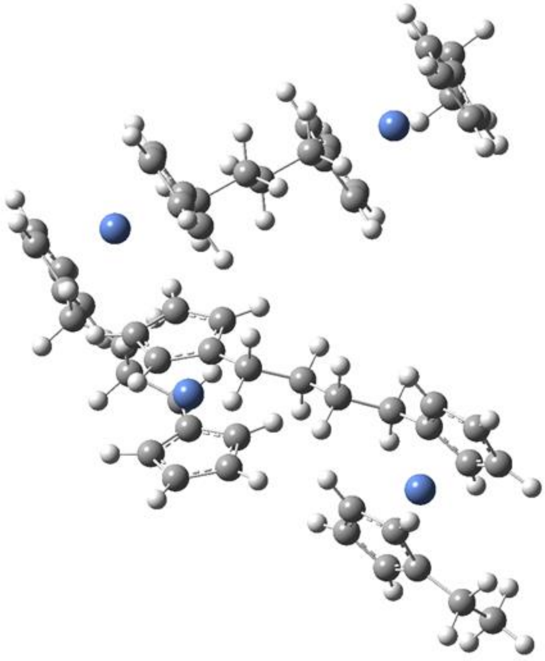

2d E(BS1) = −2857.4779 a.u.

	x	y	z
Ni1	4.524297	2.667765	0.170898
Ni2	11.05295	−3.116328	−0.251227
Ni3	−3.978948	4.242365	−0.470313

Ni4	−11.341484	−3.390411	0.186045	C46	−11.458503	−2.425518	2.185767
C1	10.77666	−4.578026	−1.901988	C47	−10.118560	−2.316327	1.715355
C2	9.885846	−3.491416	−2.135963	C48	−10.697650	−4.546422	−1.588842
C3	8.951590	−3.424593	−1.057453	C49	−12.095644	−4.295362	−1.703828
C4	9.288616	−4.475495	−0.151256	C50	−12.770687	−4.946340	−0.626805
C5	10.40657	−5.190168	−0.671679	C51	−11.769247	−5.591629	0.161116
C6	12.80718	−2.990789	1.175896	C52	−10.496138	−5.349691	−0.431323
C7	11.70995	−2.251053	1.711041	C53	−8.294158	0.222768	0.247498
C8	11.39057	−1.192823	0.811175	C54	−8.902559	−1.100546	−0.261705
C9	12.28544	−1.272528	−0.292722	C55	−14.266348	−5.005469	−0.436605
C10	13.15038	−2.381733	−0.069994	C56	−14.734618	−5.082611	1.022498
C11	13.54064	−4.141276	1.820796	H1	11.60059	−4.872712	−2.539822
C12	12.69646	−5.001314	2.770141	H2	9.914820	−2.828424	−2.991537
C13	7.759915	−2.508365	−0.930728	H3	8.767990	−4.694777	0.773547
C14	7.968138	−1.074342	−1.443052	H4	10.89246	−6.040331	−0.209959
C15	6.711133	−0.205364	−1.308303	H5	11.20263	−2.456658	2.644975
C16	6.932289	1.239189	−1.784003	H6	10.60451	−0.460941	0.946277
C17	5.733437	2.149355	−1.682754	H7	12.29574	−0.619227	−1.156080
C18	5.780984	3.556908	−1.444431	H8	13.94208	−2.713135	−0.731659
C19	4.459919	4.082541	−1.529375	H9	14.40113	−3.742983	2.380332
C20	3.580664	2.998915	−1.810242	H10	13.96875	−4.777277	1.034933
C21	4.363457	1.811339	−1.900198	H11	12.30782	−4.410740	3.607522
C22	5.163121	1.604446	2.008185	H12	13.29802	−5.814170	3.191872
C23	3.778789	1.340860	1.795639	H13	11.84123	−5.444411	2.248029
C24	3.056653	2.568989	1.893587	H14	6.912467	−2.953225	−1.476623
C25	4.015545	3.594646	2.155426	H15	7.447410	−2.473506	0.121837
C26	5.309066	3.002671	2.228501	H16	8.277269	−1.100083	−2.497249
C27	1.555426	2.707300	1.830158	H17	8.797996	−0.612516	−0.891502
C28	1.036735	4.043024	1.275356	H18	5.893825	−0.662809	−1.883102
C29	−0.495120	4.116038	1.232796	H19	6.380226	−0.193059	−0.261076
C30	−1.010720	5.452695	0.676305	H20	7.269173	1.212207	−2.832700
C31	−2.510986	5.592657	0.602587	H21	7.764196	1.681043	−1.218568
C32	−3.477954	5.105214	1.534156	H22	6.680161	4.126316	−1.240703
C33	−4.768327	5.557507	1.131565	H23	4.175358	5.117891	−1.390704
C34	−4.611123	6.326043	−0.056129	H24	2.506227	3.061266	−1.928247
C35	−3.224857	6.341103	−0.382483	H25	3.978746	0.819529	−2.101642
C36	−3.897954	3.312469	−2.491850	H26	5.961220	0.873214	1.997606
C37	−5.234312	3.214930	−2.009580	H27	3.337266	0.370606	1.600787
C38	−5.238732	2.410568	−0.829403	H28	3.799618	4.648854	2.275155
C39	−3.887091	2.017930	−0.588270	H29	6.239460	3.527890	2.404802
C40	−3.061759	2.570119	−1.610716	H30	1.146047	1.883128	1.229882
C41	−6.449096	1.983604	−0.041501	H31	1.143348	2.568223	2.842501
C42	−7.059440	0.660703	−0.549395	H32	1.441025	4.197317	0.265715
C43	−10.114104	−1.530336	0.522791	H33	1.419695	4.870104	1.889336
C44	−11.468649	−1.161922	0.261836	H34	−0.887409	3.293661	0.619274
C45	−12.296567	−1.709861	1.284040	H35	−0.892412	3.960311	2.245497

H36	−0.588778	5.612945	−0.325322
H37	−0.608640	6.266824	1.300390
H38	−3.269236	4.494973	2.403962
H39	−5.702533	5.342208	1.635028
H40	−5.404470	6.799587	−0.620736
H41	−2.776935	6.840559	−1.233584
H42	−3.573452	3.868094	−3.362713
H43	−6.105985	3.679392	−2.455632
H44	−3.550731	1.403849	0.238793
H45	−1.988950	2.453350	−1.697414
H46	−6.182379	1.869051	1.017988
H47	−7.215924	2.768911	−0.084037
H48	−6.293248	−0.126206	−0.507216
H49	−7.323817	0.772859	−1.610317
H50	−11.806296	−0.567943	−0.579357
H51	−13.372092	−1.607834	1.355592
H52	−11.782565	−2.969727	3.063928
H53	−9.244982	−2.760069	2.178319
H54	−9.926373	−4.179506	−2.254318
H55	−12.575347	−3.712098	−2.481038
H56	−11.944905	−6.168790	1.060277
H57	−9.543128	−5.701325	−0.056174
H58	−8.030036	0.110641	1.308483
H59	−9.060918	1.009076	0.204976
H60	−8.134160	−1.884511	−0.219503
H61	−9.168294	−0.985052	−1.321234
H62	−14.721565	−4.131524	−0.920505
H63	−14.657730	−5.880940	−0.977539
H64	−14.395061	−4.210685	1.592256
H65	−15.828410	−5.122026	1.075930
H66	−14.348188	−5.978846	1.520885

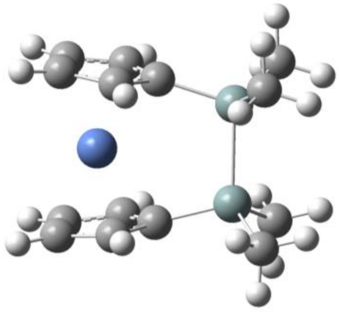

6 E(BS1) = −1295.5130 a.u.

	**x**	**y**	**z**
Ni	1.528636	0.000002	0.000036
Si	−1.678730	1.199556	0.000018
Si	−1.678723	−1.199558	−0.000023
C	0.999105	1.818343	1.146033
C	0.137464	1.719530	0.000198
C	0.998751	1.817900	−1.145960
C	2.345451	1.967099	−0.712700
C	2.345663	1.967380	0.712286
C	0.998754	−1.817950	1.145925
C	2.345665	−1.967350	−0.712330
C	0.999109	−1.818290	−1.146070
C	0.137467	−1.719530	−0.000230
C	2.345456	−1.967130	0.712662
C	−2.531400	1.903606	1.547709
C	−2.530770	1.903333	−1.548150
C	−2.530730	−1.903330	1.548165
C	−2.531420	−1.903610	−1.547700
H	0.678577	1.767835	2.179896
H	0.677805	1.767050	−2.179670
H	3.214590	2.067406	−1.351140
H	3.214996	2.067949	1.350418
H	0.677808	−1.767150	2.179643
H	3.214999	−2.067880	−1.350460
H	0.678581	−1.767730	−2.179930
H	3.214594	−2.067470	1.351096
H	−2.065110	1.542355	2.471844
H	−2.482480	2.999414	1.555114
H	−3.588690	1.613962	1.580685
H	−2.064890	1.541032	−2.472080
H	−3.588300	1.614532	−1.580840
H	−2.480970	2.999097	−1.556380
H	−2.064840	−1.541030	2.472085
H	−3.588270	−1.614530	1.580873
H	−2.480940	−2.999100	1.556394
H	−2.065160	−1.542361	−2.471840
H	−2.482493	−2.999422	−1.555101
H	−3.588718	−1.613978	−1.580651

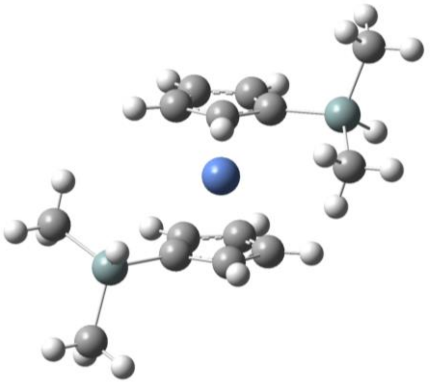

6a E(BS1) = −1296.7135 a.u.

	x	y	z
Ni	−0.011912	0.252517	0.500013
Si	−3.503848	−0.617947	−0.163812
Si	3.322925	−0.141096	−0.972788
C	−0.724140	2.373342	0.564781
C	−1.782425	1.513612	0.962902
C	−2.123947	0.646305	−0.130077
C	−1.240838	0.999754	−1.206128
C	−0.387546	2.053768	−0.782818
C	2.034684	−0.632919	0.292390
C	1.051770	−1.670580	0.152020
C	0.317545	−1.786095	1.362908
C	0.829700	−0.820068	2.276780
C	1.877198	−0.116929	1.623790
C	−3.082500	−2.036127	−1.343285
C	−5.142642	0.182873	−0.673916
C	3.862709	1.654392	−0.716812
C	4.827828	−1.287379	−0.882560
H	−0.245292	3.124338	1.181203
H	−2.249237	1.502736	1.940934
H	−1.216481	0.529399	−2.182024
H	0.390859	2.519699	−1.374333
H	0.888161	−2.261730	−0.741381
H	−0.499842	−2.470935	1.552485
H	0.469639	−0.640370	3.282329
H	2.454412	0.691056	2.057807
H	−3.662695	−1.155564	1.221821
H	−2.941805	−1.671720	−2.368295
H	−3.888057	−2.779672	−1.365993
H	−2.160953	−2.545938	−1.041206
H	−5.082817	0.595275	−1.688365
H	−5.409413	1.003293	0.002194
H	−5.961483	−0.546877	−0.655435
H	2.713195	−0.286982	−2.329521
H	4.308051	1.797716	0.275353
H	4.614244	1.946255	−1.459943
H	3.016082	2.344295	−0.805101
H	5.319293	−1.220191	0.095574
H	4.538120	−2.333271	−1.036309
H	5.568109	−1.028386	−1.649579

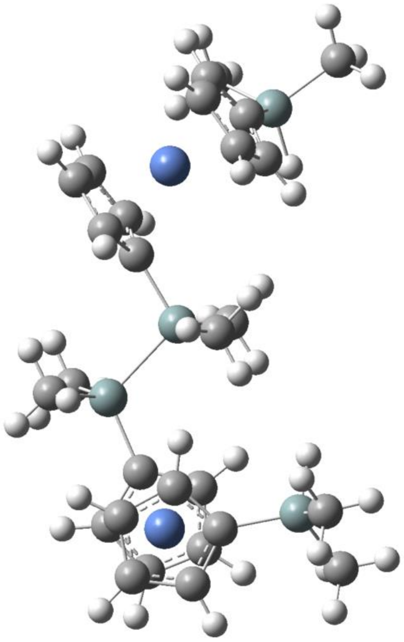

6b E(BS1) = −2592.2340 a.u.

	x	y	z
Ni	3.982668	−0.003282	−1.058505
Ni	−4.412247	−0.585792	0.153514
Si	0.561234	−0.499246	0.308805
Si	−1.047692	−2.236956	0.169229
Si	−4.487447	3.088712	0.100910
Si	5.997879	0.986568	1.854717
C	4.097280	−2.231022	−1.043393
C	3.211917	−1.817898	−0.010173
C	2.091016	−1.132014	−0.590044
C	2.319401	−1.138127	−2.008478
C	3.541640	−1.808992	−2.285712
C	5.341188	1.324883	0.135570
C	5.968430	1.009834	−1.117442
C	5.176431	1.523365	−2.178973
C	4.041855	2.166399	−1.605146
C	4.142150	2.044084	−0.193190
C	−2.691147	−1.794781	0.984805
C	−3.883944	−2.596902	0.952192
C	−4.867231	−2.014519	1.796571
C	−4.307111	−0.834235	2.365053
C	−2.981478	−0.701177	1.868194

C	−4.710139	−0.444151	−2.056590
C	−4.025626	0.716507	−1.605616
C	−4.870477	1.440398	−0.696659
C	−6.088754	0.685813	−0.604570
C	−5.991350	−0.463083	−1.433679
C	−0.033763	1.102246	−0.526691
C	1.020372	−0.139990	2.119280
C	−1.339683	−2.711614	−1.650882
C	−0.348090	−3.768979	1.060897
C	−5.433512	3.293733	1.726884
C	−4.902702	4.516930	−1.071753
C	7.144409	2.382857	2.423278
C	6.916882	−0.666499	1.919817
H	5.036197	−2.754282	−0.909675
H	3.362562	−1.987127	1.049195
H	1.669001	−0.692399	−2.75174
H	3.983048	−1.953477	−3.264287
H	6.892317	0.456414	−1.238298
H	5.385592	1.423773	−3.237084
H	3.237020	2.644528	−2.149739
H	3.424947	2.426622	0.523467
H	−4.016272	−3.503972	0.373894
H	−5.869717	−2.389600	1.963291
H	−4.807782	−0.153783	3.042713
H	−2.302759	0.105829	2.116161
H	−4.320746	−1.193041	−2.734903
H	−3.022672	1.007145	−1.894238
H	−6.942629	0.941317	0.011877
H	−6.747052	−1.229839	−1.553012
H	−3.019721	3.133576	0.379065
H	−0.223998	0.950042	−1.596082
H	0.722955	1.890860	−0.433971
H	−0.962544	1.468844	−0.074744
H	1.373398	−1.042000	2.632922
H	0.157870	0.237071	2.681486
H	1.817407	0.611051	2.181907
H	−1.761142	−1.877760	−2.223299
H	−2.036071	−3.555956	−1.731291
H	−0.398412	−3.008735	−2.128437
H	−0.205752	−3.580311	2.131340
H	0.620586	−4.062040	0.638033
H	−1.029872	−4.623261	0.964189
H	−6.517810	3.254119	1.566144
H	−5.204904	4.261124	2.189792
H	−5.172547	2.507903	2.444455
H	−5.972879	4.534182	−1.311070
H	−4.353770	4.425864	−2.016119
H	−4.642318	5.484008	−0.624180
H	4.826637	0.940105	2.781496
H	8.022021	2.465452	1.770666
H	6.628182	3.349608	2.407480
H	7.501380	2.209331	3.445924
H	7.765486	−0.676988	1.224695
H	7.312199	−0.855531	2.925003
H	6.257383	−1.500095	1.654154

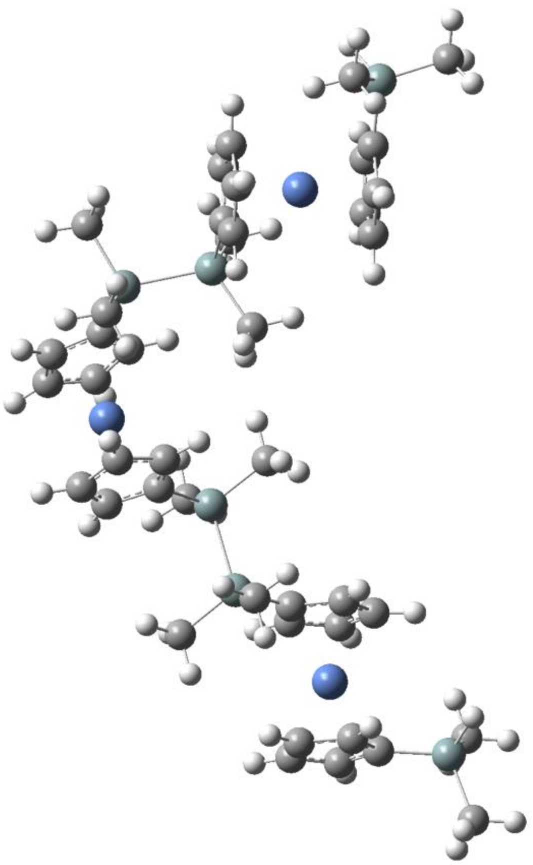

6c E(BS1) = −3887.7559 a.u.

	**x**	**y**	**z**
Ni	6.626435	−1.912374	0.241785
Ni	0.402267	3.945541	0.075255
Ni	−7.408850	−1.127905	−0.142965
Si	4.240086	0.807378	−0.538250
Si	4.073364	3.106229	0.021218
Si	−2.423021	1.568769	−0.578084
Si	−4.430898	0.980270	0.536618
Si	−9.499943	−4.120500	0.207583
Si	9.391798	−4.265773	−0.252330
C	8.104679	−0.270630	0.571917
C	7.144632	0.165601	−0.381077
C	5.845630	0.193923	0.231598

C	6.042437	−0.238580	1.588023	H	7.163471	−3.209405	−2.347745
C	7.419580	−0.521787	1.796217	H	2.568542	5.945120	0.334571
C	7.558219	−3.891213	−0.246453	H	0.486514	6.570383	−1.240452
C	6.635019	−4.062458	0.840781	H	−0.064083	4.425730	−2.785125
C	5.326255	−3.732576	0.399111	H	1.682964	2.485381	−2.163664
C	5.412669	−3.350755	−0.971473	H	0.858003	3.432709	2.933103
C	6.774447	−3.446046	−1.364345	H	0.138254	1.330621	1.420200
C	2.539397	3.945012	−0.687550	H	−2.450819	4.682275	0.390842
C	2.119956	5.290021	−0.403106	H	−0.745800	5.507238	2.292769
C	1.021610	5.628511	−1.238291	H	−4.712723	−2.254171	0.278679
C	0.730393	4.496296	−2.052892	H	−6.184251	−3.321631	−1.695078
C	1.655008	3.470520	−1.713920	H	−7.306534	−1.295090	−3.088816
C	0.054358	3.416123	2.207634	H	−6.522676	1.014158	−1.967541
C	−0.327365	2.308282	1.401181	H	−9.361738	1.020239	0.445620
C	−1.419686	2.692873	0.551427	H	−10.269557	−1.137160	−0.868601
C	−1.690249	4.069636	0.861047	H	−7.774868	−2.522172	2.428209
C	−0.793503	4.510514	1.871333	H	−7.821817	0.163812	2.492468
C	−5.369225	−0.261778	−0.522617	H	−8.327479	−4.946709	0.627824
C	−5.275697	−1.693780	−0.457990	H	2.738209	−0.123305	1.258417
C	−6.051597	−2.263887	−1.503800	H	2.909062	−1.269080	−0.079883
C	−6.643533	−1.195456	−2.238111	H	1.833237	0.133195	−0.239099
C	−6.227481	0.024551	−1.639147	H	5.175445	1.099476	−2.861940
C	−9.158114	−0.012150	0.702063	H	3.421079	0.913563	−2.917995
C	−9.633857	−1.156731	0.008825	H	4.453846	−0.505744	−2.668890
C	−9.123398	−2.340010	0.643454	H	3.251108	2.797683	2.386828
C	−8.320780	−1.883859	1.743398	H	4.071007	4.356590	2.206378
C	−8.343325	−0.464144	1.780765	H	5.021810	2.866875	2.330484
C	2.793235	−0.206546	0.166370	H	5.621492	3.975578	−1.770454
C	4.331051	0.555998	−2.422117	H	6.529367	3.555024	−0.306119
C	4.103655	3.299061	1.914863	H	5.599573	5.063703	−0.376644
C	5.600997	4.007774	−0.674978	H	−3.329029	3.426796	−2.029807
C	−2.838364	2.462231	−2.206333	H	−3.513127	1.853881	−2.820750
C	−1.412237	0.007367	−0.970616	H	−1.930521	2.653571	−2.791218
C	−5.502330	2.530635	0.798400	H	−1.113296	−0.525478	−0.060332
C	−4.035758	0.194603	2.222266	H	−0.501202	0.255609	−1.529291
C	−11.022330	−4.739047	1.149573	H	−2.001374	−0.688103	−1.580004
C	−9.771362	−4.321555	−1.654821	H	−5.803473	2.982543	−0.154186
C	10.163291	−3.899839	1.436722	H	−4.953973	3.291275	1.367052
C	9.712211	−6.070847	−0.728069	H	−6.416304	2.286669	1.353480
H	9.164208	−0.405787	0.392814	H	−3.455824	−0.72972	2.114792
H	7.360298	0.423687	−1.410961	H	−4.953329	−0.05101	2.771006
H	5.263114	−0.343722	2.333518	H	−3.445491	0.882197	2.839327
H	7.865154	−0.883202	2.714968	H	−11.916433	−4.164955	0.877900
H	6.89512	−4.379539	1.843942	H	−10.883585	−4.643107	2.232586
H	4.426251	−3.748343	1.001430	H	−11.221195	−5.795079	0.928989
H	4.588692	−3.030355	−1.596887	H	−10.613570	−3.710861	−2.002941

H	−9.995666	−5.364994	−1.906616
H	−8.884121	−4.021371	−2.223320
H	10.026268	−3.393627	−1.286870
H	9.701764	−4.507537	2.224909
H	11.236479	−4.124857	1.433766
H	10.040311	−2.846677	1.712592
H	9.268487	−6.758326	0.002254
H	9.280361	−6.304724	−1.707964
H	10.787719	−6.281421	−0.776775

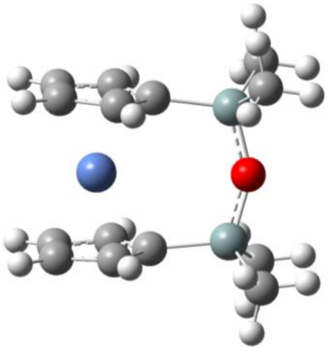

13 E(BS1) = −1370.8420 a.u.

	x	y	z
Ni	−1.533838	−0.000622	−0.000077
Si	1.621090	−1.611669	0.000126
Si	1.619727	1.612856	0.000035
O	2.040169	0.000762	0.000573
C	−2.459844	−1.912738	−0.713117
C	−1.108496	−1.854225	−1.146767
C	−0.243342	−1.812170	0.000073
C	−1.108760	−1.854105	1.146739
C	−2.460005	−1.912686	0.712782
C	−2.461420	1.910931	0.713138
C	−1.110046	1.853041	1.146772
C	−0.244863	1.811776	−0.000126
C	−1.110320	1.853573	−1.146737
C	−2.461585	1.911264	−0.712745
C	2.347428	−2.398679	1.550367
C	2.347403	−2.397743	−1.550615
C	2.345591	2.399537	−1.550603
C	2.345331	2.400383	1.550363
H	−3.335072	−1.946917	−1.350321
H	−0.783429	−1.829757	−2.180078
H	−0.783879	−1.829640	2.180105
H	−3.335376	−1.946747	1.349797
H	−3.336660	1.944469	1.350365
H	−0.784950	1.828676	2.180073
H	−0.785470	1.829488	−2.180127
H	−3.336974	1.944999	−1.349750
H	1.930866	−1.953452	2.461872
H	3.434093	−2.256235	1.583133
H	2.146615	−3.476392	1.582444
H	1.931823	−1.950976	−2.461816
H	2.145571	−3.475216	−1.584111
H	3.434229	−2.256311	−1.582594
H	1.930631	1.952318	−2.461864
H	3.432563	2.259188	−1.582350
H	2.142694	3.476809	−1.584193
H	1.929803	1.954114	2.461828
H	2.142939	3.477781	1.582996
H	3.432223	2.259537	1.582640

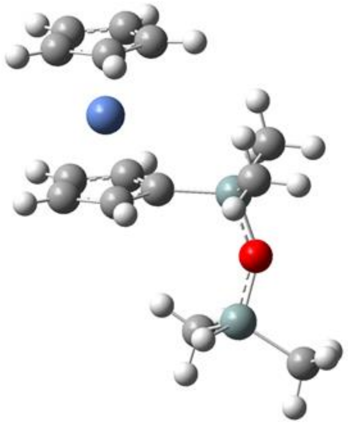

13a E(BS1) = −1372.0268 a.u.

	x	y	z
Ni	2.059119	−0.394624	−0.054182
Si	−1.255040	1.153390	0.120316
Si	−3.964922	−0.449264	−0.342284
O	−2.865195	0.718477	0.092442
C	1.012240	−2.305241	0.466675
C	0.287949	−1.216098	1.019191
C	−0.179319	−0.368890	−0.044457
C	0.281908	−0.977265	−1.262493
C	1.008394	−2.156689	−0.951222
C	3.036630	1.613574	0.071433
C	3.428056	0.844312	1.204631
C	4.143885	−0.294360	0.736586
C	4.194155	−0.229150	−0.685338
C	3.509422	0.949824	−1.096801
C	−0.944693	2.001000	1.773180
C	−0.948255	2.332552	−1.317968
C	−5.670899	0.344816	−0.365510
C	−3.901552	−1.895860	0.862293
H	1.497963	−3.097483	1.023118

H	0.124878	−1.044845	2.076541
H	0.110682	−0.593050	−2.260865
H	1.489329	−2.817108	−1.662357
H	2.476001	2.539411	0.094210
H	3.213908	1.080282	2.239414
H	4.568181	−1.076940	1.352929
H	4.662750	−0.954000	−1.339055
H	3.368414	1.280433	−2.118109
H	−3.640398	−0.949320	−1.713651
H	−1.122489	1.316862	2.611373
H	−1.617778	2.857182	1.899892
H	0.086226	2.366136	1.851168
H	−1.119762	1.839819	−2.282561
H	0.079046	2.715832	−1.316561
H	−1.629376	3.190006	−1.261615
H	−5.942123	0.726851	0.625933
H	−6.438120	−0.378637	−0.668548
H	−5.705975	1.185288	−1.068013
H	−4.172577	−1.580252	1.877167
H	−2.894641	−2.327353	0.902340
H	−4.595942	−2.690384	0.561158

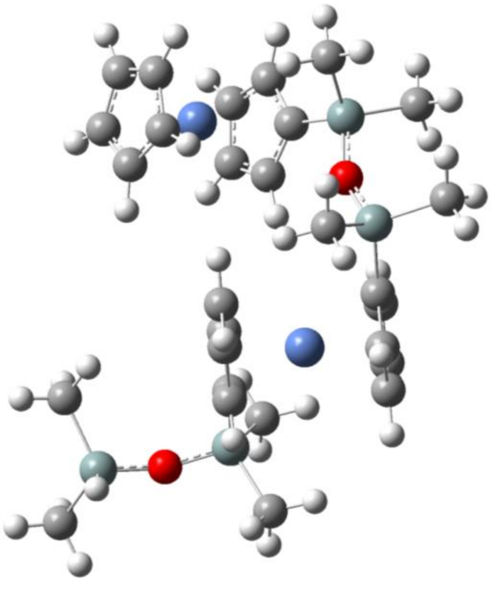

13b E(BS1) = −2742.8711 a.u.

	**x**	**y**	**z**
Ni	2.747313	2.627062	−0.181677
Ni	−1.352765	−1.915395	0.302333
Si	4.019967	−0.809961	−0.659730
Si	2.129550	−2.944181	0.846024
Si	−4.434099	−0.300350	−0.908590
Si	−5.804902	2.172436	0.560334
O	2.781979	−1.600539	0.119304
O	−5.376171	1.034109	−0.571616
C	3.071720	2.842865	−2.379269
C	4.007837	1.901774	−1.871905
C	3.298244	0.774587	−1.333748
C	1.903764	1.054939	−1.524991
C	1.764475	2.317421	−2.161583
C	2.650545	2.454128	2.041034
C	3.820322	3.190166	1.693921
C	3.409322	4.383809	1.035326
C	1.986601	4.385253	0.974286
C	1.517014	3.193590	1.596581
C	0.444944	−3.234371	0.089705
C	−0.639383	−3.992715	0.650453
C	−1.707192	−4.043689	−0.284933
C	−1.305047	−3.316729	−1.443582
C	0.007416	−2.824160	−1.214583
C	−1.687737	−0.740556	2.172257
C	−2.946083	−0.994667	1.565772
C	−2.984308	−0.369365	0.272439
C	−1.710339	0.275886	0.110418
C	−0.918127	0.049167	1.267530
C	−7.419235	2.962890	0.003739
C	5.429872	−0.458674	0.543764
C	4.660680	−1.876115	−2.081402
C	1.982344	−2.639322	2.700748
C	3.237746	−4.443746	0.542240
C	−3.832581	−0.106867	−2.682790
C	−5.508717	−1.837309	−0.718160
C	−4.440090	3.457208	0.746266
H	3.308147	3.798996	−2.829991
H	5.084615	2.025141	−1.883689
H	1.095232	0.405670	−1.213734
H	0.832477	2.805482	−2.419459
H	2.627925	1.496613	2.546010
H	4.842356	2.891065	1.889357
H	4.064114	5.150617	0.640749
H	1.369759	5.153685	0.525402
H	0.481412	2.897617	1.704550
H	−0.651223	−4.444877	1.635333
H	−2.662044	−4.533330	−0.137073
H	−1.899710	−3.158047	−2.334795
H	0.584967	−2.223911	−1.906988
H	−1.362575	−1.101525	3.140213
H	−3.745428	−1.579338	2.005104
H	−1.398094	0.838709	−0.760858
H	0.099524	0.387424	1.415958

H	−6.019869	1.502515	1.879746
H	−7.302216	3.460567	−0.966324
H	−8.211065	2.212253	−0.099909
H	−7.760496	3.713198	0.727898
H	5.074745	0.105712	1.413248
H	5.888074	−1.387875	0.904413
H	6.220287	0.132476	0.063655
H	3.854352	−2.130371	−2.779407
H	5.432939	−1.343926	−2.650991
H	5.100917	−2.813106	−1.718825
H	1.390527	−1.740686	2.907357
H	1.495764	−3.483010	3.206582
H	2.970357	−2.505949	3.157861
H	3.344277	−4.650393	−0.52892
H	4.241491	−4.293992	0.959741
H	2.814623	−5.341198	1.010187
H	−3.187681	0.773112	−2.792057
H	−3.262550	−0.982621	−3.014828
H	−4.682227	0.022017	−3.363691
H	−5.866902	−1.950573	0.312150
H	−6.390327	−1.771416	−1.366820
H	−4.960756	−2.749174	−0.984163
H	−4.280678	4.005719	−0.190125
H	−4.687808	4.188227	1.526172
H	−3.492323	2.979855	1.021353

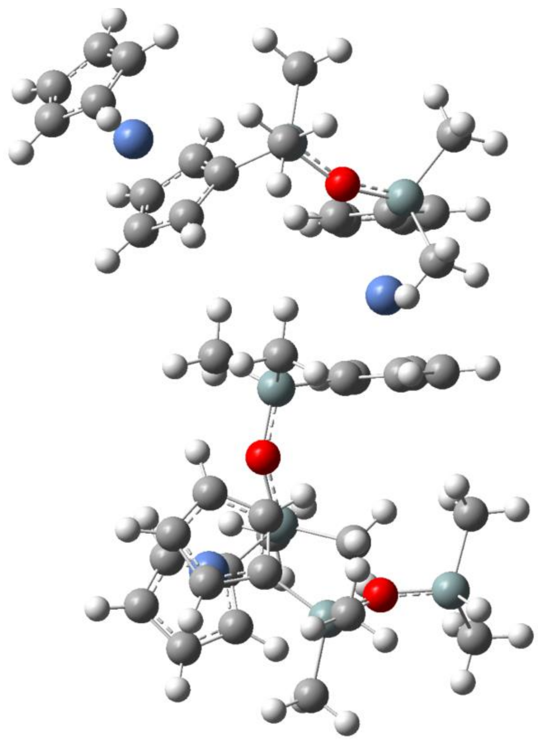

13c E(BS1) = −4113.7148 a.u.

	**x**	**y**	**z**
Ni	5.672344	−3.052044	0.245975
Ni	1.720405	3.738925	1.094981
Ni	−4.618248	−2.444873	0.817209
Si	4.984547	−0.169824	−1.909407
Si	3.468986	2.690946	−2.063408
Si	−0.234917	0.655436	1.584844
Si	−3.350986	0.636561	2.341082
Si	−6.445068	−1.298377	−2.195587
Si	−6.072732	1.791292	−2.860898
O	3.900815	1.089628	−1.998115
O	−1.806264	0.199977	1.903305
O	−5.951835	0.293673	−2.151722
C	4.575843	−1.917535	1.833808
C	5.173556	−0.991179	0.937914
C	4.615197	−1.173506	−0.374666
C	3.660848	−2.240580	−0.250944
C	3.636296	−2.694263	1.094543
C	7.159597	−4.500221	1.067917
C	6.249293	−5.209598	0.233166
C	6.375651	−4.699263	−1.090113
C	7.365086	−3.674411	−1.073228
C	7.849192	−3.550977	0.260795
C	3.465707	3.358664	−0.311834
C	3.630630	2.595302	0.892953
C	3.726110	3.482273	1.999673
C	3.618236	4.813894	1.501500
C	3.456003	4.736888	0.091764
C	−0.005362	4.771227	2.083055
C	0.071385	3.439536	2.569438
C	−0.126810	2.522819	1.480344
C	−0.326064	3.338732	0.313661
C	−0.251333	4.709217	0.680362
C	−4.349164	−0.938578	2.453049
C	−5.750019	−1.094911	2.175366
C	−6.136653	−2.432546	2.460187
C	−4.981278	−3.129987	2.918947
C	−3.892301	−2.218300	2.914393
C	−3.907349	−4.097934	−0.497747
C	−5.184240	−3.674490	−0.954783
C	−5.102489	−2.304136	−1.379353
C	−3.741174	−1.903818	−1.165659
C	−3.011630	−2.996579	−0.626509
C	4.743867	−1.226037	−3.451504
C	6.740741	0.515898	−1.821527

C	4.722818	3.673572	−3.079310	H	5.429265	−2.081642	−3.466617
C	1.784007	2.778870	−2.901705	H	3.720239	−1.614420	−3.510899
C	0.250126	−0.126411	−0.057605	H	4.922751	−0.633869	−4.357090
C	0.846162	0.012673	2.988423	H	7.482410	−0.289982	−1.768439
C	−4.113446	1.792963	1.061082	H	6.969121	1.123202	−2.705582
C	−3.293275	1.498353	4.021349	H	6.877575	1.153188	−0.939472
C	−6.663730	−1.842000	−3.991082	H	4.446122	4.734587	−3.127271
C	−8.085651	−1.487229	−1.283433	H	5.726242	3.614997	−2.642300
C	−4.342333	2.453705	−3.192722	H	4.778200	3.300892	−4.109588
C	−7.040698	2.949633	−1.735907	H	1.383898	3.800326	−2.896376
H	4.807297	−2.029078	2.886009	H	1.852743	2.449796	−3.945866
H	5.937984	−0.269777	1.201107	H	1.062853	2.130602	−2.392870
H	3.058951	−2.641131	−1.057711	H	1.300914	0.059445	−0.308291
H	3.026364	−3.498858	1.486504	H	0.105660	−1.212816	−0.014077
H	7.296534	−4.650316	2.131439	H	−0.368560	0.253134	−0.879969
H	5.572416	−5.992483	0.551361	H	1.910846	0.173231	2.782132
H	5.812730	−5.026525	−1.955047	H	0.606408	0.501666	3.940379
H	7.689456	−3.089588	−1.924559	H	0.691799	−1.064602	3.123702
H	8.604185	−2.854335	0.602700	H	−5.082886	2.176318	1.405024
H	3.679746	1.514654	0.944335	H	−3.460330	2.655787	0.882534
H	3.846636	3.198666	3.038057	H	−4.278741	1.279180	0.10753
H	3.639033	5.720966	2.093200	H	−4.300271	1.767638	4.363311
H	3.344176	5.588468	−0.569554	H	−2.847601	0.847756	4.783213
H	0.125480	5.673688	2.667681	H	−2.697950	2.418580	3.970645
H	0.266630	3.160240	3.597974	H	−6.966332	−2.895271	−4.044940
H	−0.500788	2.969396	−0.689059	H	−7.431403	−1.247698	−4.501653
H	−0.346955	5.556291	0.012044	H	−5.728541	−1.738644	−4.553720
H	−6.405929	−0.318617	1.799542	H	−8.383892	−2.541705	−1.222129
H	−7.126788	−2.852776	2.332844	H	−8.015560	−1.102181	−0.260008
H	−4.937562	−4.176156	3.196790	H	−8.891215	−0.946524	−1.795572
H	−2.872212	−2.452243	3.193956	H	−4.384989	3.434629	−3.682564
H	−3.664984	−5.075357	−0.098603	H	−3.778820	2.568720	−2.259125
H	−6.076974	−4.289072	−0.970247	H	−3.778100	1.777974	−3.845868
H	−3.342164	−0.918303	−1.369108	H	−6.535972	3.072662	−0.770474
H	−1.967539	−2.985259	−0.339918	H	−7.145168	3.942713	−2.190835
H	−6.801561	1.660270	−4.160807	H	−8.048369	2.564265	−1.541252

vi. References

1. Frisch, M. J.; Trucks, G. W.; Schlegel, H. B.; Scuseria, G. E.; Robb, M. A.; Cheeseman, J. R.; Scalmani, G.; Barone, V.; Mennucci, B.; Petersson, G. A.; Nakatsuji, H.; Caricato, M.; Li, X.; Hratchian, H. P.; Izmaylov, A. F.; Bloino, J.; Zheng, G.; Sonnenburg, J. L.; Hada, M.; Ehara, M.; Toyota, K.; Fukuda, R.; Hasegawa, J.; Ishida, M.; Nakajima, T.; Honda, Y.; Kitao, O.; Nakai, H.; Vreven, T.; Montgomery Jr., J. A.; Peralta, J. E.; Ogliaro,

F.; Bearpark, M.; Heyd, J. J.; Brothers, E.; Kudin, K. N.; Staroverov, V. N.; Keith, T.; Kobayashi, R.; Normand, J.; Raghavachari, K.; Rendell, A.; Burant, J. C.; Iyengar, S. S.; Tomasi, J.; Cossi, M.; Rega, N.; Millam, J. M.; Klene, M.; Knox, J. E.; Cross, J. B.; Bakken, V.; Adamo, C.; Jaramillo, J.; Gomperts, R.; Stratmann, R. E.; Yazyey, O.; Austin, A. J.; Cammi, R.; Pomelli, C.; Ochterski, J. W.; Martin, R. L.; Morokuma, K.; Zakrzewski, V. G.; Voth, G. A.; Salvador, P.; Dannenberg, J. J.; Dapprich, S.; Daniels, A. D.; Farkas, O.; Foresman, J. B.; Ortiz, J. V.; Cioslowski, J.; Fox, D. L., *Gaussian 09, Revision B.01*. Wallingford CT, 2010.
2. Vosko, S. H.; Wilk, L.; Nusair, M., *Canad J. Phys.* **1980,** *58* (8), 1200-1211.
3. Becke, A. D., *Phys. Rev. A* **1988,** *38* (6), 3098-3100.
4. Slater, J. C., *Quantum Theory of Molecules and Solids*. McGraw-Hill: 1974; Vol. 4.
5. Miehlich, B.; Savin, A.; Stoll, H.; Preuss, H., *Chem. Phys. Lett.* **1989,** *157* (3), 200-206.
6. Lee, C.; Yang, W.; Parr, R. G., *Phys. Rev. B* **1988,** *37* (2), 785-789.
7. Becke, A. D., *J. Chem. Phys.* **1993,** *98* (7), 5648-5652.
8. Grimme, S.; Ehrlich, S.; Goerigk, L., *J. Comput. Chem.* **2011,** *32* (7), 1456-1465.
9. Bergner, A.; Dolg, M.; Küchle, W.; Stoll, H.; Preuß, H., *Mol. Phys.* **1993,** *80* (6), 1431-1441.
10. Frisch, M. J.; Pople, J. A.; Binkley, J. S., *J. Chem. Phys.* **1984,** *80* (7), 3265-3269.
11. Hariharan, P. C.; Pople, J. A., *Theor. Chem. Acc.* **1973,** *28* (3), 213-222.
12. Cramer, C. J., *Faraday Discuss.* **2010,** *145* (0), 523-556.
13. Marten, B.; Kim, K.; Cortis, C.; Friesner, R. A.; Murphy, R. B.; Ringnalda, M. N.; Sitkoff, D.; Honig, B., *J. Phys. Chem* **1996,** *100* (28), 11775-11788.
14. Tannor, D. J.; Marten, B.; Murphy, R.; Friesner, R. A.; Sitkoff, D.; Nicholls, A.; Honig, B.; Ringnalda, M.; Goddard III, W. A., *J. Am. Chem. Soc.* **1994,** *116* (26), 11875-11882.
15. PCModel 9.0, Serena Software, Bloomington IN, USA, 2004.
